# One-pot synthesis of *E*-chalcones using a multifunctional catalyst comprised of ruthenium nanoparticles and palladium N-heterocyclic carbene complexes immobilized on silica†

**DOI:** 10.1039/d4sc07773c

**Published:** 2025-03-12

**Authors:** Manisha Durai, Yufei Wu, Jacob Johny, Walid Hetaba, Thomas Wiegand, Walter Leitner, Alexis Bordet

**Affiliations:** a Max Planck Institute for Chemical Energy Conversion Stiftstr. 34-36 Mülheim an der Ruhr 45470 Germany alexis.bordet@cec.mpg.de walter.leitner@cec.mpg.de; b Institut für Technische und Makromolekulare Chemie, RWTH Aachen University Worringerweg 2 Aachen 52074 Germany

## Abstract

The integrated one-pot synthesis of valuable *E*-chalcones from aryl iodides, phenylacetylenes, CO and H_2_ is achieved using a single catalyst material. The multifunctional catalytic system is composed of a silica support on which ruthenium nanoparticles and covalently functionalized palladium N-heterocyclic carbene (NHC) complexes are jointly assembled. The resulting Ru@SiO_2_-[Pd–NHC] catalyst was characterized by a variety of techniques including N_2_ physisorption, solid-state nuclear magnetic resonance, electron microscopy, and X-ray photoelectron spectroscopy to collect information on its textural, structural, morphological, and electronic properties. Ru@SiO_2_-[Pd–NHC] was found to be active and selective for the one-pot synthesis of a wide variety of *E*-chalcones, valuable products with widespread applications in the fine chemical, agrochemical, and pharmaceutical industries. The heterogenized Pd–NHC complex catalyzed the carbonylative Sonogashira coupling step, while CO-covered Ru NPs were found to be responsible for the highly selective hydrogenation of the ynones intermediates to *E*-chalcones. This study outlines the potential of hybrid multifunctional catalytic systems combining molecular and nanoparticle sites to open up new and more sustainable complex reaction sequences toward valuable compounds.

## Abstract 2

The one-pot synthesis of valuable *E*-chalcones is achieved using a multifunctional Ru@SiO_2_-[Pd–NHC] catalytic system combining molecular and nanoparticle sites for efficient carbonylative Sonogashira coupling and selective hydrogenation.

## Graphical abstract

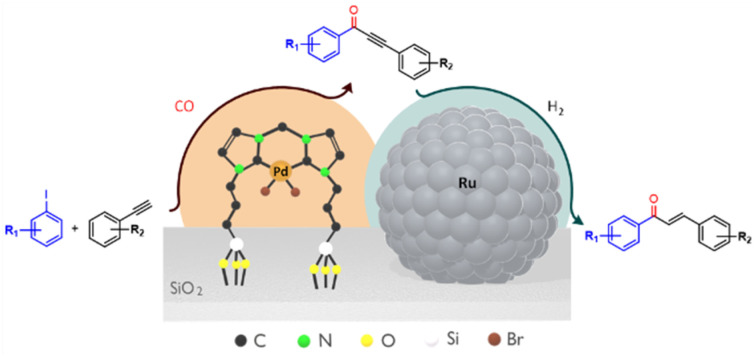

## Introduction

Chalcones are highly valuable α,β-unsaturated ketones from the flavonoid family and are key compounds for the preparation of pharmaceuticals (*e.g.* anticancer, antimicrobial, anti-inflammatory, antidiabetic, antipyretic, antiallergic, analgesic and antimalarial agents),^1–4^ fluorescent probes,^5^ anti-corrosion agents,^6^ pesticides,^7^ liquid crystals,^8,9^ and various organic molecules (*e.g.* flavones, isoxazole, pyrazole, pyrazoline, pyrimidine, thiazine and imidazoline derivatives).^10–15^ Chalcones are typically prepared by Claisen–Schmidt condensation of aromatic ketones and aldehydes catalyzed by strong bases or acids.^16^ Alternative synthetic routes include Suzuki–Miyaura coupling,^17^ Stille coupling,^18^ decarboxylative cross coupling,^19^ Sonogashira isomerization coupling,^20^ Photo-fries rearrangement,^21^ and carbonylative Heck coupling.^22–24^ However, these methods suffer from various limitations including low functional group tolerance, low availability of substrates, low reaction rates, unwanted side reactions, and use of excess reagents, ligands and additives.^25^

In this context, we have designed a new one-pot synthetic route providing access to *E*-chalcones *via* carbonylative Sonogashira coupling of aryl iodides, phenylacetylenes, and CO integrated with subsequent selective hydrogenation of the corresponding ynones. The combined immobilization of heterogeneous metal nanoparticles (NPs) and homogeneous molecular catalysts onto a multifunctional catalyst material was envisaged as attractive strategy to enable the sequential catalytic processes into one synthetic operation and eliminate energy-consuming steps such as separation and purification.^26–30^ Prieto, Vorholt, and Leitner *et al.* reported a one-pot Fischer–Tropsch synthesis/hydroformylation sequence to produce long-chain alcohols from syngas using a combination of heterogeneous (NaPr-CoRu/AOmM) and homogeneous ([HCo(CO)_3_(PCy_3_)]) Co catalysts.^31^ Qihua Yang *et al.* observed synergistic interactions between jointly supported Ru NPs and Rh-bipyridine complexes in the regeneration of nicotinamide adenine dinucleotide (NADH).^32^ Individual components of the catalytic system were found to be inactive owing to the weak interaction of NAD^+^ at the Ru NPs surface and the limited ability of the cationic Rh complex to dissociate H_2_. Recently, our group reported a multifunctional catalytic system where Ru NPs and Cu–NHC-complexes (NHC = N-heterocyclic carbene ligands) were immobilized on the same silica support and used for the one-pot synthesis of allyl- and alkylamines.^33^ This hybrid catalyst proved highly active and selective, enabling the synthesis of a wide range of allylamines and alkylamines, including those used in pharmaceuticals, while minimizing side reactions and simplifying purification compared to traditional multistep methods.

**Fig. 1:**
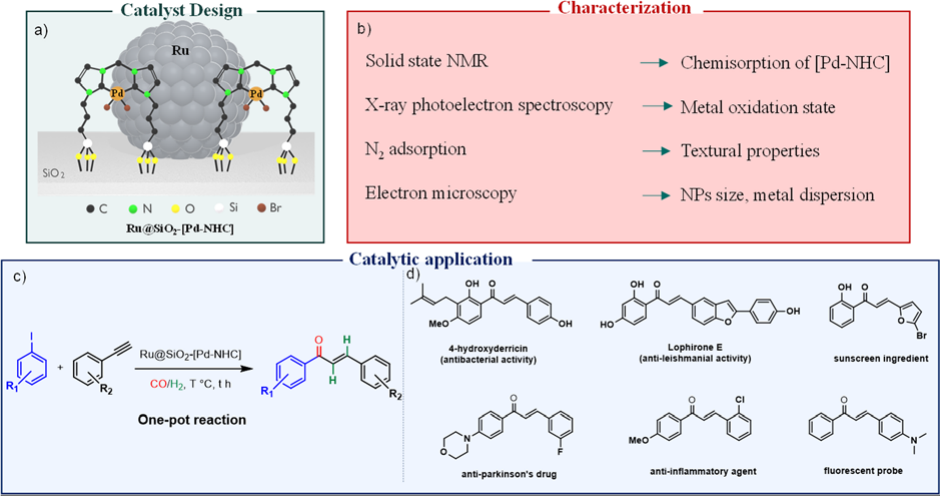
Approach followed in this work. (a) Ideal catalyst design, (b) characterization techniques, (c) catalytic application in the synthesis of *E*-chalcones, and (d) selected examples of valuable bio-active *E*-chalcone products.^2^

The first successful example of combining metal NPs and NHC-complexes in a multifunctional catalyst opens the door to explore the large variety of catalytic reactions enabled by the NHC-ligand scaffold as a broadly applicable toolbox for multistep reaction cascades. Herein, we further explore the potential and versatility of this strategy and report a new catalyst comprising the joint assembly of Ru NPs and covalently functionalized Pd–NHC complexes on a silica support for the integrated one-pot synthesis of *E*-chalcones from aryl iodides, phenylacetylenes, CO and H_2_ (Fig. 1). The molecular complex enables the carbonylative Sonogashira coupling, while the Ru NPs are responsible for the hydrogenation step to *E*-chalcones.

## Results and discussion

### Catalyst design and synthesis

**Fig. 2:**
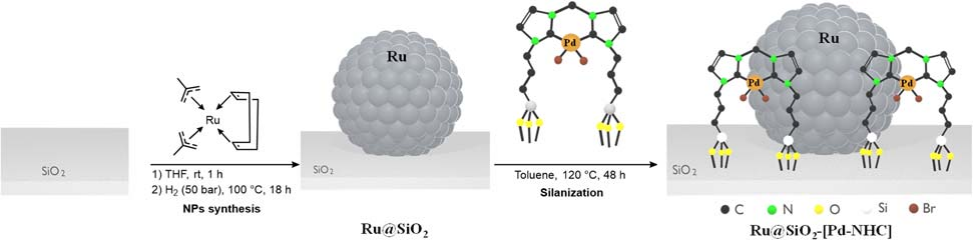
Synthetic approach for the immobilization of Ru NPs and [Pd–NHC] jointly on silica to form idealized Ru@SiO_2_-[Pd–NHC].

The multifunctional Ru@SiO_2_-[Pd–NHC] catalyst was prepared through a two-step synthetic approach (Fig. 2). First, the immobilization of Ru NPs on SiO_2_ was carried out by following a previously reported procedure.^23–34^ This involved the wet impregnation of SiO_2_ (amorphous, 332 m^2^ g^−1^) with a solution of [Ru (2-methylallyl)_2_(cod)] in THF. After evaporation of the solvent under vacuum, the impregnated SiO_2_ was subjected to an atmosphere of H_2_ (50 bar) at 100 °C for 18 h, yielding a black powder with a theoretical Ru loading of 0.2 mmol g^−1^ (2 wt%). The selected ethoxysilane-functionalized [Pd–NHC] complex [(3,3′-methylene bis(1-(3-triethoxysilyl)propyl)-2,2′-3,3′-tetrahydro-1*H*-imidazole-2-yl)palladium(ii)dibromide] has a structure inspired from the imidazolium based ionic liquid-like molecular modifiers used to prepare supported ionic liquid phases,^35^ and was synthesized by adapting protocols from previous reports.^36^ In brief, NHC.Br [(3,3′-methylene bis(1-(3-triethoxysilyl)propyl)-1*H*-imidazole-3-ium)dibromide] (1 equiv.) was added to a solution of [Pd(acac)_2_] (1 equiv.) in THF and heated sequentially at 60 °C for 5 h and 110 °C for 1 h (see ESI† for characterization data). In the second step, the resulting [Pd–NHC] complex was covalently grafted on Ru@SiO_2_ by silanization. The complex [Pd–NHC] (0.2 mmol) was added to a suspension of Ru@SiO_2_ (1 g, 0.2 mmol of Ru) in toluene and was heated at 120 °C for 48 h under argon atmosphere to generate a bimetallic bifunctional Ru@SiO_2_-[Pd–NHC] catalyst with a theoretical Pd loading of 0.2 mmol g^−1^ (2.1 wt%) (see ESI† for detailed synthetic procedures).

### Catalyst characterization

**Fig. 3:**
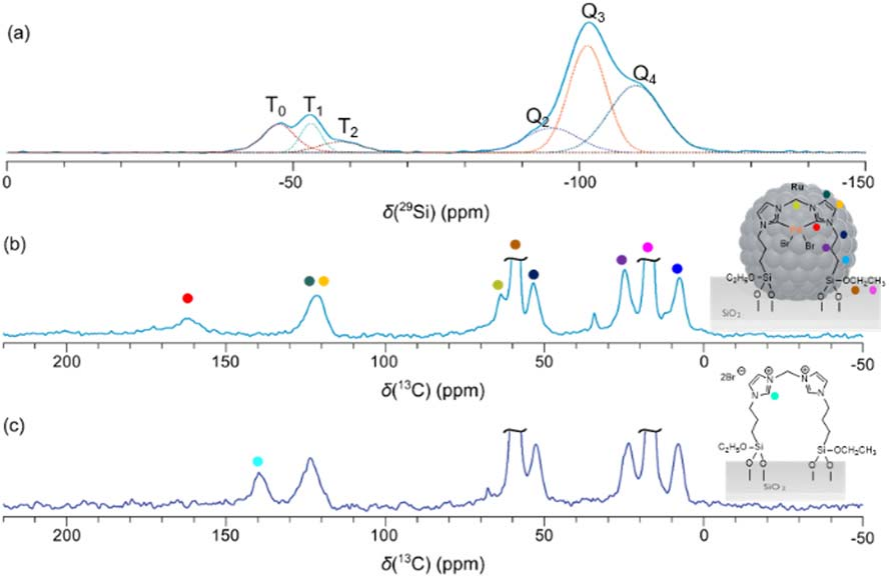
(a) ^1^H–^29^Si CP-MAS NMR spectrum of Ru@SiO_2_-[Pd–NHC] before catalysis recorded at 11.7 T and 17.0 kHz MAS. Dashed lines show the decomposition of the spectrum to individual resonances. (b) and (c) ^1^H–^13^C CP-MAS NMR spectra recorded at 11.7 T and 17.0 kHz MAS (b).

Ru and Pd loadings (Table S1†) were determined by inductively coupled plasma-optical emission spectrometry (ICP-OES) to 0.19 mmol g^−1^ (1.86 wt%) and 0.19 mmol g^−1^ (1.97 wt%) for Ru and Pd respectively, which is well in agreement with theoretical values (2 wt%). The Brunauer–Emmett–Teller (BET) surface area of the support (SiO_2_, 332 m^2^ g^−1^) obtained from N_2_ physisorption experiments did not change upon Ru loading (Ru@SiO_2_, 325 m^2^ g^−1^). A substantial decrease was observed after silanization of the [Pd–NHC] complex (Ru@SiO_2_-[Pd–NHC], 289 m^2^ g^−1^), as expected (Table S1†). ^1^H–^29^Si cross-polarization (CP) spectra recorded under magic-angle spinning (MAS) conditions (Tables S2 and S3†) of Ru@SiO_2_-[Pd–NHC] (Fig. 3a) show Si species which can be assigned to different chemical environments according to their chemical-shift values.^37,38^ Firstly, silanol groups on the surface of the silica support are detected, which can be described by using the “Q_*n*_-nomenclature” (with Q referring to [SiO_4_] tetrahedra and *n* being the number of bridging Si–O–Si groups). Spectral deconvolution of the ^29^Si CP-MAS NMR spectrum as given in Fig. 3a reveals resonances at −110 ppm (Q_4_), −101 ppm (Q_3_) and −95 ppm (Q_2_). Secondly, silicon atoms of the ethoxysilane-functionalized [Pd–NHC] complex covalently grafted on the silica support are present that can be described by using the “T_*n*_-nomenclature” (with T referring to [RSiO_3_] tetrahedra and *n* being the number of C–Si–O^surface^ groups). ^29^Si resonances at −58 ppm (T_2_), −53 ppm (T_1_) and −47 ppm (T_0_) have been detected (Table S4†). The T_2_ and T_1_ signals correspond to the Si atoms of [Pd–NHC] covalently bound to the SiO_2_ surface and thus provide clear evidence for the successful grafting of the complex to the silica support.^35,39,40^ The assignment of the resonance at −47 ppm to T_0_ units is further supported by comparing the spectrum to one of the non-grafted NHC.Br where one of the two ^29^Si resonances is observed at −46 ppm (Fig. S1†).^41^ This suggests that part of the triethoxysilane functionalities did however not react with the SiO_2_ surface during the silanization reaction and the [Pd–NHC] catalyst is only immobilized *via* one of the two organic linkers. Note that the discussed ^29^Si spectra are not quantitative due to the CP-polarization transfer employed. ^1^H–^13^C CP-MAS NMR spectra revealed the Pd-C resonance at a chemical-shift value of ∼162 ppm characteristic for the Pd-coordinated NHC carbene,^42^ indicating that the structure of the Pd^II^ complex was preserved upon grafting (Fig. 3b).

This is further supported by comparing the ^13^C CP-MAS spectra of the immobilized [Pd–NHC] catalyst with the one of SiO_2_-[NHC.Br] in the absence of Pd^2+^, where the same ^13^C resonance is more shielded (140 ppm, Fig. 3c). Pd-coordination to the immobilized NHC.Br thus leads to a ^13^C high-frequency shift of about 22 ppm, which is of similar magnitude than the chemical-shift change observed for the non-immobilized NHC.Br upon [Pd–NHC] complex-formation (see Fig. S2b and c†). The ^13^C NMR chemical-shift values between the “free” [Pd–NHC] complex and the immobilized one are very similar, both in the presence and absence of Ru nanoparticles (Fig. 3b, S2a and b†).

Transmission Fourier transform infrared spectroscopy (FT-IR) of the [Pd–NHC] complex (Fig. S3†) showed signals characteristic of C–H stretching in imidazole cycles and *N*-alkyl chains (2884–3092 cm^−1^ region), as well as C=N stretches (1714 cm^−1^) and symmetric ring stretches (1563 and 1521 cm^−1^), in agreement with literature reports.^43–45^ The same signals can be observed in the spectrum of Ru@SiO_2_-[Pd–NHC] (Fig. S3†), confirming that the structure of the [Pd–NHC] complex was preserved upon chemisorption on Ru@SiO_2_.

**Fig. 4:**
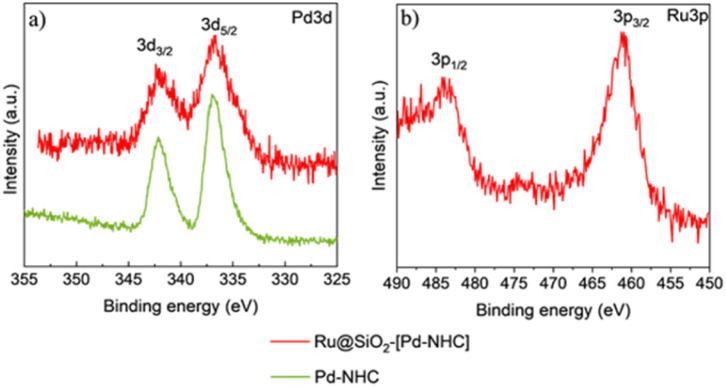
Characterization of Ru@SiO_2_-[Pd–NHC] and [Pd–NHC] by X-ray photoelectron spectroscopy. High resolution XPS spectra of (a) Pd3d and (b) Ru3p.

X-ray photoelectron spectroscopy (XPS) measurements were carried out to study the electronic structure of Ru@SiO_2_-[Pd–NHC] using the “free” [Pd–NHC] complex as a reference. The high resolution XPS spectra of Pd3d (Fig. 4a) showed that Pd species in [Pd–NHC] and Ru@SiO_2_-[Pd–NHC] are in (+2) oxidation state, with binding energy values (3d_5/2_ at ∼337.1 eV and 3d_3/2_ at ∼342.5 eV) and at N1s ∼400.0 eV (Fig. S4a†) matching well with literature data on Pd–NHC species.^46^ Interestingly, prolonged exposure to the X-ray beam led to a partial reduction of the Pd species, giving a mixture of Pd^II^ and Pd^0^, evidencing the beam-sensitivity of the chemisorbed [Pd–NHC] complex (Fig. S5†). The Ru3d_5/2_ (BE ∼280.0 eV) (Fig. S4b†) and Ru3p_3/2_ (BE ∼461.0 eV) (Fig. 4b) signals revealed the presence of Ru^0^ on Ru@SiO_2_-[Pd–NHC].^47^

**Fig. 5:**
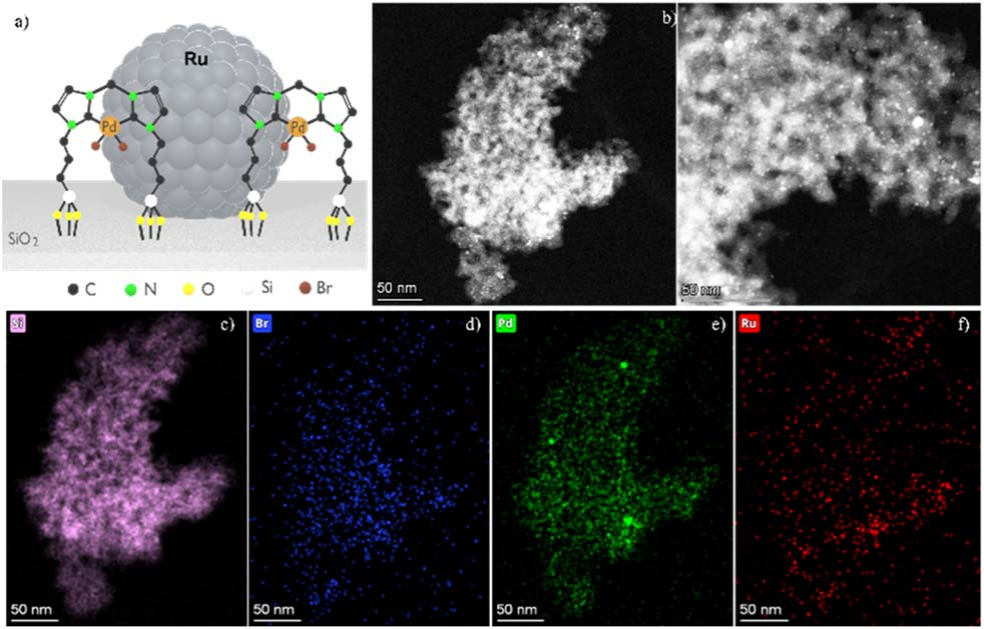
Schematic depiction (a), and electron microscopy (b)–(f) of Ru@SiO_2_-[Pd–NHC]. (b) STEM-HAADF, and (c)–(f) EDX elemental mapping of (c) Si-Kα; (d) Br-Kα; (e) Pd-Lα; (f) Ru-Kα.

Characterization of Ru@SiO_2_-[Pd–NHC] by scanning transmission electron microscopy using the high-angle annular dark-field detector (STEM-HAADF) showed small and well dispersed Ru NPs (1.8 ± 0.4 nm, Fig. 5 and S6†) on the support. Elemental mapping using energy-dispersive X-ray spectroscopy (EDX) confirmed that Ru NPs and [Pd–NHC] are homogeneously present on the SiO_2_ support (Fig. 5c–f). Notably, exposure to the electron beam also damaged the [Pd–NHC] complex, with the formation of Pd NPs over time, as shown for a Pd–NHC@SiO_2_ reference (1.3 ± 0.4 nm, Fig. S7†). Taken together, these results demonstrate the co-existence of Ru NPs and [Pd–NHC] species at the surface of the same SiO_2_ support material.

### Catalytic study

**Scheme 1:**
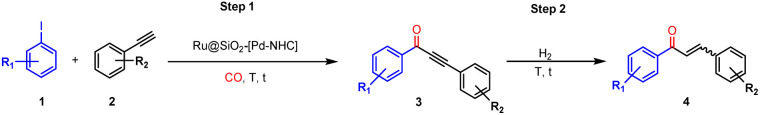
One-pot synthetic approach to chalcones through carbonylative Sonogashira coupling and selective hydrogenation.

Ru@SiO_2_-[Pd–NHC] was applied to the one-pot synthesis of chalcones (Scheme 1). The reaction sequence consists of two consecutive steps: (a) carbonylative Sonogashira coupling of aryl iodides and phenylacetylenes in presence of CO to form ynones (3), followed by (b) selective hydrogenation of 3 to chalcones using molecular H_2_. The one-pot two step synthesis of 4 from iodobenzene and phenylacetylene was selected as a model reaction.

### Carbonylative sonogashira coupling

Step 1 was first investigated separately to identify suitable reaction conditions. The carbonylative Sonogashira coupling of phenyl iodide (0.3 mmol) and phenyl acetylene (1 equiv.) with Ru@SiO_2_-[Pd–NHC] (2 mol% Pd) and reference catalysts was tested using Fisher-Porter bottles under conditions adapted from literature:^48^ triethyl amine as a base, toluene as a solvent, at 100 °C, with 4 bar CO, for 4 h (Table 1). The reaction mechanism typically proceeds through the oxidative addition of aryl iodide to a Pd(0) complex to form an aryl-Pd(ii) intermediate, followed by CO insertion to generate the corresponding acyl–Pd complex. This complex reacts with an alkyne in the presence of a base to finally afford the carbonylative coupling product *via* reductive elimination.^49,50^ The free [Pd–NHC] complex was tested first, delivering promising activity and selectivity (72% of 3, entry 1). In contrast, Pd/C (5 wt% Pd, 3.5 nm Pd NPs) and Ru@SiO_2_ (2 wt% Ru, 1.6 ± 0.4 nm Ru NPs) gave low yields of 3 (11% and 2%, respectively, entry 2–3), indicating that Pd and Ru NPs are poorly active under these conditions. Using Ru@SiO_2_-[Pd–NHC], moderate conversion (29%) and yield of 3 (27%) were observed (entry 4), demonstrating that the immobilized [Pd–NHC] complex is still active, although its performance is reduced as compared to the free complex in toluene. The detailed testing/optimization of the different reaction parameters can be found in the ESI (Tables S5–S11†). Interestingly, changing the solvent from toluene to dimethylformamide (DMF) or propylene carbonate (PC) enhanced catalytic performances drastically (Table 1, entries 5–6), with yields reaching 79% and 78%, respectively. This effect is rationalized by a better dispersion of the Ru@SiO_2_-[Pd–NHC] catalyst in these more polar solvents. In accordance with principles of “green chemistry”, PC was selected as the reaction solvent in the following.

**Table 1:** Testing of catalysts and reaction parameters for the carbonylative Sonogashira coupling of iodobenzene (1) and phenylacetylene (2) in presence of CO^a^

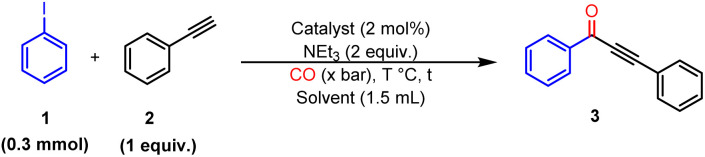							
Entry	Catalyst	Solvent	CO (bar)	Temp. (°C)	Time (h)	Conv.^b^ (%)	Yield 3^b^ (%)
1	[Pd–NHC]	Toluene	4	100	4	74	72
2	5% Pd/C	Toluene	4	100	4	12	11
3	Ru@SiO_2_	Toluene	4	100	4	2	2
4	Ru@SiO_2_-[Pd–NHC]	Toluene	4	100	4	29	27
5	Ru@SiO_2_-[Pd–NHC]	DMF	4	100	4	86	79
6	Ru@SiO_2_-[Pd–NHC]	PC	4	100	4	81	78
7	Ru@SiO_2_-[Pd–NHC]	PC	10	100	4	98	87
8	Ru@SiO_2_-[Pd–NHC]	PC	2	100	4	83	80
9	Ru@SiO_2_-[Pd–NHC] 1 mol% Pd	PC	2	100	4	70	68
10	Ru@SiO_2_-[Pd–NHC] 3 mol% Pd	PC	2	100	4	84	80
11	Ru@SiO_2_-[Pd–NHC]	PC	2	80	4	50	47
12	Ru@SiO_2_-[Pd–NHC]	PC	2	120	4	87	81
13	Ru@SiO_2_-[Pd–NHC]	PC	2	100	12	87	83
14	Ru@SiO_2_-[Pd–NHC]	PC	2	100	16	93	88

a 2 mol% Pd catalyst was used unless otherwise mentioned.

b Conv. and yields were determined by GC-FID using mesitylene as internal standard. Byproducts are 1,2-diphenylethyne, 1,4-diphenylbuta-1,3-diyne and benzene.

Raising the CO pressure to 10 bars in a steel autoclave resulted in higher conversion (98%), albeit at slight expense of selectivity (87% yield of 3, 89% selectivity, entry 7). Good yield of 3 (83%) at excellent selectivity (96%) was observed when using lower CO pressure (2 bar, entry 8), which was thus used as standard pressure for the rest of the study as it allowed using simple glass reactors (Fisher-Porter bottles) instead of steel autoclaves.

**Fig. 6:**
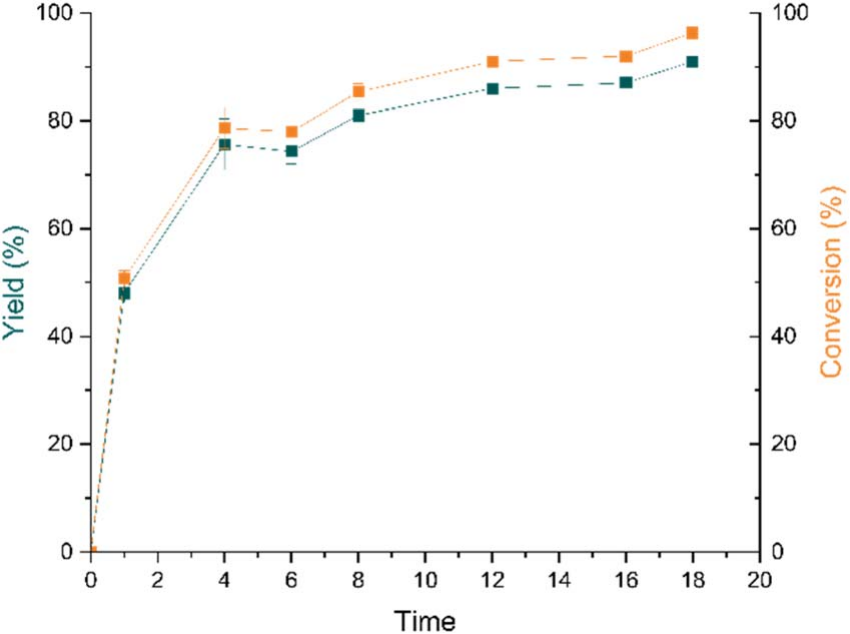
Time profile of carbonylative sonogashira coupling under optimized reaction conditions: Ru@SiO_2_-[Pd–NHC] (30 mg; 0.012 mmol total metal loading), iodobenzene (0.3 mmol; 50 equiv. w.r.t. to [Pd–NHC]), phenylacetylene (0.3 mmol), NEt_3_ (0.6 mmol), PC (1.5 mL), CO (2 bar), 100 °C; yield was determined by GC-FID using mesitylene as internal standard. Byproducts are 1,2-diphenylethyne, 1,4-diphenylbuta-1,3-diyne and benzene. Data points are average of three experiments and error bars represent standard deviations.

Lowering the catalyst loading and temperature from the standard conditions gave lower conversions, while an increase in these parameters did not result in better performance (entries 9–12). Replacing NEt_3_ by other bases such as K_2_CO_3_ or DIPEA resulted in low conversions (Table S6†). Recording a time profile under optimized conditions (Table 1, entry 14) revealed an expected apparent first order kinetic with a yield of 3 reaching 90% after 16 h (Fig. 6). A hot filtration test was performed separating the Ru@SiO_2_-[Pd–NHC] catalyst under optimized conditions after 30 min from the reaction mixture by cannula filtration (Table S12†). The negligible change in yield of 3 after filtration of the solid catalyst demonstrates that the reaction is catalyzed by the surface-bound Pd-complex and not due to any leached Pd-species.

### Hydrogenation

Once the optimal conditions for the carbonylative coupling reaction were identified, we proceeded to investigate the hydrogenation step in a one-pot two-step sequence. This involved releasing the CO pressure after 16 h and pressurizing with H_2_ without any intermediate work-up or isolation (see ESI† for detailed procedure). Treating the reaction mixture containing the intermediate ynone-under 2 bar of H_2_ at 100 °C for 3 h gave 71% yield of *E*-1,3-diphenylprop-2-en-1-one (4), along with 20% of the intermediate 3 remaining unreacted (Table 2, entry 1). Extending the reaction time to 8 h (entry 2) and raising the H_2_ pressure to 3 bar (entry 3) allowed reaching 95% yield of 4. The same reaction using the reference SiO_2_-[Pd–NHC] catalyst without Ru NPs afforded poor hydrogenation of 3 to 4 (25%, entry 6), indicating that the hydrogenation activity of Ru@SiO_2_-[Pd–NHC] is dominated by Ru NPs. Increasing the scale of the reaction (1 mmol, x3) and substrates concentrations resulted in a faster reaction (Table S11†) and 4 was obtained in 96% yield within 6 h instead of 24 h (entries 7 and 8).

**Table 2:** One-pot two-step synthesis of chalcone 4 – study of the hydrogenation step^a^

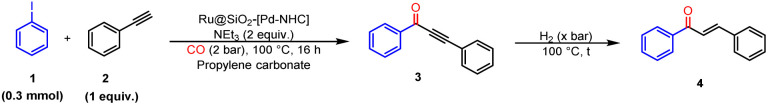					
Entry	P(H_2_) bar	Time (h)	Conv. (%)^b^	Yield 3 (%)^b^	Yield 4 (%)^b^
1	2	3	98	20	71
2	2	8	99	9	86
3	3	8	>99	0	95
4	4	8	>99	0	93
5	5	8	>99	0	94
6^c^	3	2	98	69	25
7^d^	3	3	>99	0	96
8^d^	3	2	>99	0	96

a Reaction conditions: step 1: Ru@SiO_2_-[Pd–NHC] (30 mg, 0.012 mmol total metal loading), iodobenzene (0.3 mmol, 50 equiv. w.r.t. Pd–NHC), phenylacetylene (0.3 mmol), NEt_3_ (0.6 mmol), PC (1.5 mL), CO (2 bar), 100 °C, 16 h. Step 2: H_2_ (2–3 bar), 100 °C, 2–8 h.

b Conversion and yield were determined through GC-FID using mesitylene as internal standard.

c Pd–NHC@SiO_2_ was used as reference catalyst.

d Step 1: Ru@SiO_2_-[Pd–NHC] (100 mg, 0.04 mmol total metal loading), iodobenzene (1 mmol, 50 equiv. w.r.t. Pd–NHC), phenylacetylene (1 mmol), NEt_3_ (2 mmol), PC (1.3 mL), CO (2 bar), 100 °C, 4 h. Step 2: H_2_ (3 bar), 100 °C, 2 h.

**Scheme 2:**
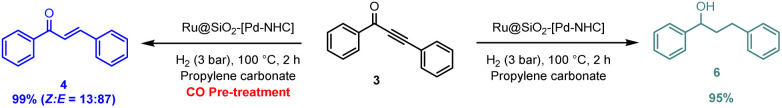
Impact of CO exposure on the reactivity of Ru@SiO_2_-[Pd–NHC].

Interestingly, the hydrogenation of 3 was chemo- and stereoselective towards the *E*-chalcone 4 even after 8 h and increasing H_2_ pressures (entries 4–5). To gain more insights into this surprising selectivity for Ru NPs under these conditions, control experiments for the direct hydrogenation of 3 were performed (Scheme 2).

Pristine Ru@SiO_2_-[Pd–NHC] under previously optimized hydrogenation conditions led to a quantitative yield (95%) of the saturated alcohol 1,3-diphenylpropan-1-ol (6), evidencing the expected C=C and C=O hydrogenation activity of the immobilized Ru NPs. In contrast, pre-treating Ru@SiO_2_-[Pd–NHC] under an atmosphere of CO prior to hydrogenation resulted in a selectivity switch, with 4 being obtained as the product in 99% yield. Time profiles corresponding to these two experiments were recorded under adapted conditions (60 °C, 2 bar H_2_) to slow down the reactions and facilitate the observation of intermediates (see ESI† for details). The pristine catalyst rapidly hydrogenated 3 to 5, followed by a slower ketone hydrogenation to 6 (Fig. S8a†). 4 was not observed as an intermediate, indicating very fast alkyne and alkene hydrogenation. Pre-treating Ru@SiO_2_-[Pd–NHC] with CO before hydrogenation reduced the overall hydrogenation activity and led to excellent selectivities toward 4, even at extended reaction times (Fig. S8b†). Conducting the hydrogenation of 3 with a pristine catalyst directly under a mixture of CO and H_2_ also gave 4 as the only product, but with a much lower reaction rate (Fig. S8c†). These results indicate that CO partially poisons the catalyst, leading to slower reactions rates and enhancing the selectivity toward 4 by suppressing over-hydrogenation steps.

The role of CO in modulating hydrogenation activity was investigated by transmission FT-IR characterization of Ru@SiO_2_-[Pd–NHC] and reference materials using CO as a molecular probe (Fig. S9†). The FT-IR spectrum of Ru@SiO_2_ exhibited bands at 2064, 1998, and 1877 cm^−1^ corresponding to terminal Ru–(CO)_2_ and bridged Ru_*n*_–CO bands,^51,52^ while Pd@SiO_2_ showed bands at 1947 and 1697 cm^−1^ characteristic of bridged Pd_2_–CO and Pd_4_–CO species.^53,54^ The FT-IR spectrum of SiO_2_-[Pd–NHC] was found similar to that of pristine SiO_2_, suggesting minimal interactions with CO. Interestingly, the band corresponding to terminal Ru-(CO)_2_ species shifted to 2064 cm^−1^ for the multifunctional Ru@SiO_2_-[Pd–NHC] catalyst, while a new band characteristic of Pd^+^-CO species was observed at 2122 cm^−1^. These two bands were not detected in a physical mixture of Ru@SiO_2_ and SiO_2_-[Pd–NHC]. These results indicate a distinct binding mode of CO at the surface of Ru@SiO_2_-[Pd–NHC] as compared to individual components of the catalysis system, presumably due to electronic interactions between Ru NPs and chemisorbed [Pd–NHC] complex that are in close proximity at the SiO_2_ surface. We hypothesize that these interactions modulate the adsorption mode and strength of CO at the Ru NPs surface, preventing the typical complete poisoning of Ru NPs by CO and enabling the selective hydrogenation of alkynes to alkenes by suppressing more difficult over-hydrogenation steps. Unravelling the nature of these interactions and proposing a detailed mechanism will require dedicated in-depth investigations.

**Scheme 3:**
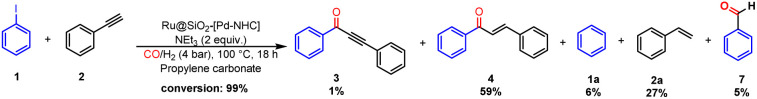
Tandem one-pot synthesis of chalcones using syngas and Ru@SiO_2_-[Pd–NHC].

Since the hydrogenation activity of the Ru NPs were modulated significantly upon exposure to CO, the direct one-step conversion of the aryl iodide 1 and the alkyne 2 in simultaneous presence of CO and H_2_ to form the *E*-chalcone 4 was attempted (Scheme 3). In addition to integrating the two reactions into one single operation, the use of the 1 : 1 mixture of the two gases known as synthesis gas would be significantly more cost effective than the two gases individually.

Interestingly, the one-pot/one-step tandem reaction performed by pressurizing the reactor directly with 4 bar of syngas gave a promising yield of 4 reaching 59% after 18 h. The competing hydrogenation of phenylacetylene to unreactive styrene was found to be the major limitation in the reaction yield. Besides, benzene and benzaldehyde were observed in low yields. While this preliminary result opens promising perspective for continuous-flow operation of the concept, the one-pot/two-step method was evaluated further in this study.

### Recycling experiments

**Fig. 7:**
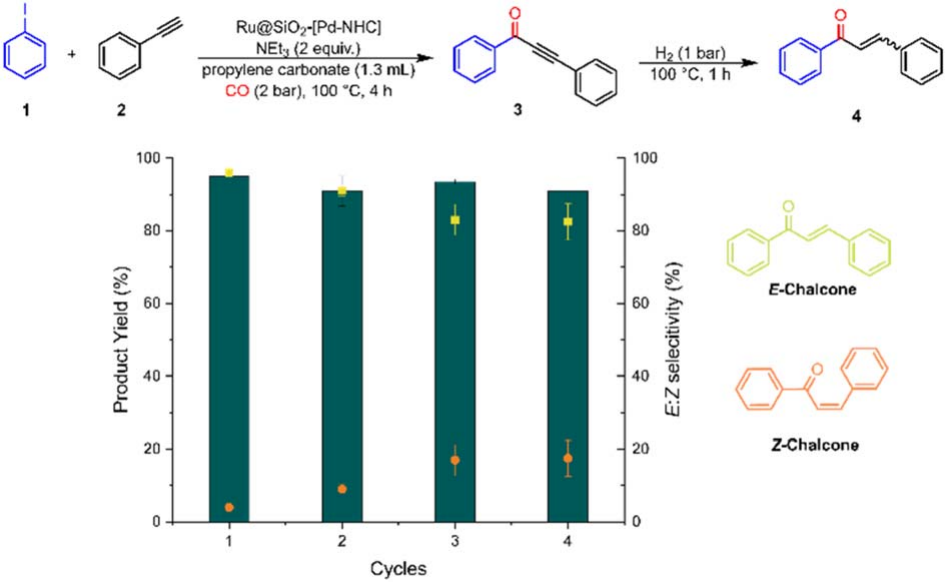
Recycling experiments for the one-pot synthesis of chalcones 4 using Ru@SiO_2_-[Pd–NHC].

The stability and reusability of Ru@SiO_2_-[Pd–NHC] was explored through recycling experiments (Fig. 7) for the one pot synthesis of 4 under adapted conditions (2 bar CO, 100 °C, 4 h followed by 1 bar H_2_, 100 °C, 1 h). Satisfyingly, catalytic performances remained fairly constant for 4 cycles, with only slight decreases in yield for the hydrogenated coupling product from 95% to 91%. While the chemo-selectivity of the hydrogenation of the triple bond to the double bond remained almost perfect, the *E* : *Z* selectivity was found to be somewhat less robust changing from 96 : 4 to 86 : 14 ratio in favor of the *E*-isomer.

After four cycles, the used catalyst was washed with propylene carbonate and acetonitrile, dried and characterized. Elemental analysis by ICP-OES revealed minimal metal leaching with a total loss of 4% Pd and 7% Ru detected after four cycles (Table S1†). ^1^H–^29^Si CP-MAS NMR spectra confirmed the robustness of the [Pd–NHC] covalent linkage on SiO_2_, and even revealed an improved grafting under catalytic conditions, as shown by a more prominent T_2_-signal for the material after catalysis (Fig. S10†). The ^1^H–^13^C CP-MAS NMR spectrum of the used catalyst reveals broader resonances pointing to a more heterogenous sample after catalysis. The Pd-C resonance (∼170 ppm) is slightly high-frequency shifted compared to the initial catalyst suggesting a change in the Pd coordination environment.

Several additional resonances appear in the spectrum of the used catalyst compared to the catalyst before reaction, for instance the resonance at ∼140 ppm (Fig. S11b†), which has also been observed in the spectrum of SiO_2_@NHC.Br (Fig. S2c†) and assigned to the ^N^C^N^ imidazolium carbon atom. This suggests a partial degradation of the complex. FT-IR spectra of the used Ru@SiO_2_-[Pd–NHC] catalyst did not show any significant changes (Fig. S12†). XPS measurements showed no significant change in the oxidation state of Pd and Ru (Fig. S13†), although detection of the potential presence of free NHC and Pd^0^ in the spent catalyst was challenging due to the relatively high noise levels and X-ray beam damage. A slight increase in Ru NPs size (2.4 nm) was observed using STEM-HAADF-EDX and STEM bright-field (STEM-BF) images, although without any noticeable agglomeration (Fig. S14 and S15†). Recycling experiments were also performed under optimized, harsher conditions, delivering excellent yields of the *E*-chalcone 4 (92–96%) for 5 cycles accounting for 30 h of total reaction time without catalyst make-up or regeneration (Fig. S16†). The catalytic activity – mainly the hydrogenation of 3 to 4 – gradually declined after the fifth cycle, presumably due to the progressive accumulation of iodide salt and/or CO at the surface of the catalyst. This hypothesis is consistent with the regeneration of most of the initial activity (80% yield of 4) after the 8th catalytic cycle by washing and drying the used Ru@SiO_2_-[Pd–NHC] catalyst (see ESI† for details). These recycling experiments highlight the robustness of the developed catalyst material as well as its simple regeneration, making it a potentially promising candidate for practical application.

### Synthetic scope

**Fig. 8:**
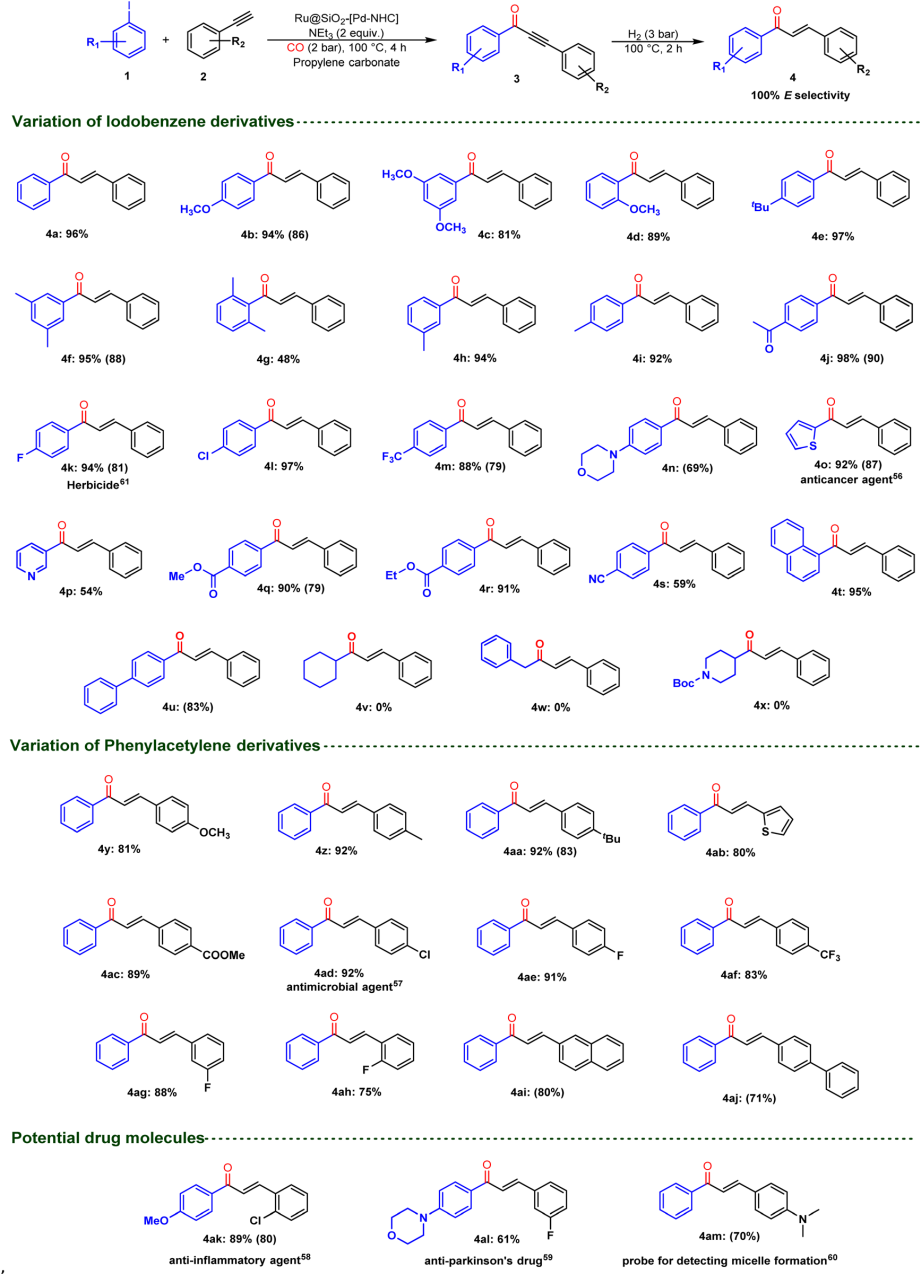
Substrate scope for the synthesis of *E*-chalcones. Reaction conditions. Step 1: Ru@SiO_2_-[Pd–NHC] (100 mg, 0.04 mmol total metal loading), iodobenzene (1 mmol, 50 equiv. w.r.t. Pd–NHC), phenylacetylene (1 mmol), NEt_3_ (2 mmol), PC (1.3 mL), CO (2 bar), 100 °C, 4 h. Step 2: H_2_ (3 bar), 100 °C, 2 h. Yields were determined through GC-FID using mesitylene as internal standard. Isolated product yield in parentheses.

The optimized conditions were then applied to assess the synthetic applicability of this tandem catalytic approach to synthesize a variety of stereo-defined *E*-chalcones (Fig. 8). Hence, iodobenzene derivatives with diverse functionalities reacted effectively with phenylacetylene in presence of Ru@SiO_2_-[Pd–NHC] to give the corresponding *E*-chalcones in moderate to excellent yields (4a–4u, 48–98%). Poor conversions were observed when using bromobenzene and styrene as substrates (Table S13†). Moderate yield was observed for *o*-dimethyl-iodobenzene (4g, 48%), presumably due to steric hindrance. 4p was also obtained in moderate yield (54%) due to the reaction of 3-iodopyridine in the competing non-carbonylative Sonogashira coupling (35% yield of 3-(phenylethynyl)pyridine). Very little competing formation of non-carbonylative Sonogashira products was observed in most other cases, indicating that Ru@SiO_2_-[Pd–NHC] is very selective toward the formation of intermediate ynones. Alkyl iodide substrates (4v–4x) were found unreactive, consistent with the known limitations of most Pd-based catalysts in carbonylative Sonogashira coupling.^55^ Notably, functionalities like halo (4k–4m), morpholine (4n) and cyano (4s) were well tolerated in this protocol. Furthermore, electron-rich and electron-deficient phenylacetylene derivatives were examined under optimized conditions and yielded the corresponding *E*-chalcones (4y–4aj) in high to excellent yields (71–92%).

Satisfyingly, valuable *E*-chalcones finding application in the treatment of cancers (4o, 87%),^56^ infections (4ad, 92%),^57^ inflammations (4ak, 80%),^58^ Parkinson's disease (4al, 61%),^59^ as well as in bioimaging (4am, 70%)^60^ and herbicides (4k, 81%)^61^ were isolated in high yields. To further outline the practicability of this approach, the potential anti-inflammatory compound 4ak (market value of approximately 620 € g^−1^) was prepared and isolated in analytically pure form on gram-scale (1.13 g, 69% isolated yield, see ESI† for Experimental details).

Interestingly, systematic comparison of this new one-pot tandem synthetic approach to standard state-of-the-art carbonylative Heck coupling^24^ method for the synthesis of (*E*)-3-phenyl-1-(thien-2-yl)prop-2-en-1-one (4o, anti-cancer properties)^56^ showed the superiority of our proposed alternative route, especially in terms of atom economy, safety, and yield (Fig. S17 and see ESI† for detailed description).

## Conclusions

A novel one-pot two step integrated approach to the synthesis of *E*-chalcones is introduced. This involves the use of a bifunctional catalytic system possessing nanoparticles and molecular active sites on a single support to achieve the carbonylative Sonogashira coupling of aryl iodides, phenylacetylenes, and CO, followed by the selective hydrogenation of the resulting ynones. In particular, [Pd–NHC] complexes heterogenized on SiO_2_ are responsible for the coupling step to produce ynones, while Ru NPs immobilized on the same support catalyze the selective hydrogenation of yones to *E*-chalcones. The Ru@SiO_2_-[Pd–NHC] catalyst proved very active, selective, and versatile, providing access to a large variety of valuable *E*-chalcones. The practicability, scalability, and improved sustainability of this strategy as compared to state-of-the-art synthetic approaches was demonstrated for a real world product, (*E*)-3-phenyl-1-(thien-2-yl)prop-2-en-1-one that finds application as an anti-cancer agent. This study further demonstrates the potential of the modular NPs@SiO_2_-[M] design to prepare multifunctional catalytic systems capable of executing complex multistep catalytic processes.

## Data availability

The data supporting this article have been included as part of the ESI.†

## Author contributions

M. D. designed and performed experiments, analysed and interpreted catalytic results, wrote the original draft. Y. W. and T. W. performed, analysed solid-state NMR experiments. J. J. and W. H. performed, analysed XPS measurements. W. L. and A. B. conceptualized, supervised, wrote and edited the manuscript. All authors contributed in reviewing and editing the manuscript.

## Conflicts of interest

There are no conflicts to declare.

## Supplementary Material

SC-OLF-D4SC07773C-s001
