# Supplementary material for: One-pot synthesis of E-chalcones using a multifunctional catalyst comprised of ruthenium nanoparticles and palladium N-heterocyclic carbene complexes immobilized on silica
Source: Chem Sci. 2025 Mar 12;16(14):5776–85. doi: 10.1039/d4sc07773c (PMC11898272; doi:10.1039/d4sc07773c)
Supplement: SC-016-D4SC07773C-s001 [file SC-016-D4SC07773C-s001.pdf]

Supplementary Information (SI) for Chemical Science.
This journal is © The Royal Society of Chemistry 2025

Supporting Information

One-Pot Synthesis of *E*-Chalcones Using a Multifunctional Catalyst Comprised of Ruthenium Nanoparticles and Palladium *N*-Heterocyclic Carbene Complexes Immobilized on Silica

Manisha Durai,[a,b] Yufei Wu,[a] Jacob Johny,[a] Thomas Wiegand,[a,b] Walter Leitner,[a,b] Alexis Bordet[a]**

[a] Max-Planck Institute for Chemical Energy Conversion, Stiftstr. 34-36, 45470 Mülheim an der Ruhr, Germany. E-Mail: alexis.bordet@cec.mpg.de, walter.leitner@cec.mpg.de

[b] Institut für Technische und Makromolekulare Chemie, RWTH Aachen University, Worringerweg 2, 52074 Aachen, Germany.

Contents

1. Safety Warning 2
2. General Methods 2
3. Analytics 2
4. Synthesis of Ru@SiO$_2$-[Pd-NHC] 5
   4.1. Synthesis of Ru@SiO$_2$ 5
   4.2. Synthesis of [Pd-NHC] complex [(3,3′-methylene bis(1-(3-triethoxysilyl)propyl)-2,2′-3,3′-tetrahydro-1H-imidazol-2-yl)palladium (II)dibromide] 6
   4.3. Synthesis of Ru@SiO$_2$[Pd-NHC] 8
5. General Procedure for the Synthesis of Chalcones 8
6. Time profiles for hydrogenation of 3 9
7. Gram-scale Synthesis 10
8. Catalyst Recycling 10
9. Supplementary Tables and Figures 12
   9.1. Catalyst Characterization 12
   9.2. Catalytic Study 17
10. Synthetic Approach Evaluation 25
11. Analytical Data for Isolated Chalcones 27
12. ^{1}H and ^{13}C NMR Spectra of Isolated Products 33
References 55

1. Safety Warning

High-pressure experiments with compressed CO or H_2 were carried out with 10 mL stainless steel autoclaves following safety precautions. To protect the autoclaves and avoid cross-contaminations, all the reactions were performed in glass inlets using a magnetic stirrer and an aluminum-heating block. Low pressure reactions (< 5 bar) were carried out in Fisher-Porter bottles placed behind a protective blast shield. The catalytic tests were repeated at least two times to ensure reproducibility.

2. General Methods

If not otherwise stated, the synthesis of ionic liquids (ILs), [Pd-NHC], Ru@SiO_2, and Ru@SiO_2-[Pd-NHC] were carried out under an argon atmosphere using standard Schlenk techniques or in a glovebox, as previously reported or with modified protocols. Solvents for air- and moisture-sensitive experiments were used from a solvent purification system (MBraun-SPS-7) and stored directly in the glovebox over molecular sieves (3 and 4 Å). All synthesized ILs, complexes, and catalysts were stored under an argon atmosphere. Silica from Merck (Grade 10184, pore size 100 Å, 63–200 µm) was dehydroxylated under vacuum at 500 °C for 16 h. [Ru(2-Me-allyl)$_2$(cod)] was obtained from Alfa Aesar. All other chemicals and solvents were purchased from commercial sources and used without purification.

3. Analytics

Solution-state NMR

All solution-state NMR spectra were recorded on a Bruker Ascend 400 MHz and 500 MHz spectrometer at room temperature. The coupling constants (J) are given in Hertz (Hz), and the chemical shifts (δ) are expressed in ppm, relative to TMS at 25 °C. The peak multiplicities were designated as follows: s = singlet; d = doublet; t = triplet, dd = doublet of doublet, ddd = doublet of doublets of doublets, dt = doublet of triplet, m = multiplet.

Solid-state NMR

^{1}H-^{13}C solid-state cross-polarization magic-angle spinning (CP-MAS) NMR spectra were recorded on a wide-bore Bruker 500 MHz (11.7 T) spectrometer (^{13}C Larmor frequency of 125.7 MHz). ^{1}H-^{29}Si CPMAS spectra were recorded on wide-bore Bruker 500 MHz (11.7 T) or 700 MHz (16.4 T) spectrometers (^{29}Si Larmor frequency of 99.3 and 139.1 MHz, respectively). All spectra

were recorded with Bruker 3.2 mm triple-resonance probes in double-resonance mode at 17.0 kHz magic-angle spinning (MAS). The probe was cooled with an active cooling gas to 270 or 280 K to maintain the sample temperature at around 290 K. The spectra were processed with the software Topspin version 4.1.4 (Bruker Biospin). ^{1}H-^{13}C cross-polarization parameters were optimized on a ^{13}C-labelled glycine ethyl ester standard sample. ^{1}H-^{29}Si cross-polarization parameters were optimized on a ^{29}Si-labelled octakis(trimethylsiloxy)silsesquioxane standard sample. All CP-MAS NMR spectra were acquired using a ^{1}H 90° pulse length of 2.5 μs, a CP contact time of 4.5 or 5.0 ms, and a relaxation delay between 1.0 and 12.5 s. More experimental parameters are summarized in Table S2-S3. Adiabatic CP transfer under the Hartmann-Hahn condition was achieved by a ramped-amplitude CP pulse[1], with $\nu_{RF}(^{1}H)$ being swept from 48 to 72 kHz. All spectra were acquired under 90 kHz SPINAL-64[2] proton decoupling using a pulse length of 5.56 μs during data acquisition. Chemical shifts were referenced indirectly to $(CH_3)_4Si$ using secondary standards adamantane for ^{13}C (**C**H_2 δ = 38.56 ppm) and octakis(trimethylsiloxy)silsesquioxane for ^{29}Si (O**Si**$(CH_3)_3$ δ = 11.5 ppm). The spectrum decomposition analysis were performed with the software DMFit.[3]

Gas Chromatography

Gas chromatography (GC) was performed on a Shimadzu GC-2030 equipped with an FID-detector and a Rtx-1701 column from Restek.

Scanning Transmission Electron Microscopy with Energy Dispersive X- ray Spectroscopy

STEM/EDX was performed on a Thermo Scientific Talos F200X operated at 200 kV acceleration voltage. STEM imaging was performed using a camera length of 98 mm and a beam current of 99 pA (measured on the screen). EDX data was acquired using the SuperX EDX system, incorporating four SDDs with a total of 0.9sr collection angle. Samples for electron microscopy were prepared by depositing the colloidal powder onto a copper TEM grid with an amorphous carbon support film. All samples were handled under inert conditions from preparation to insertion into the TEM. To determine the NP size, the particles were analyzed using ImageJ with a count of at least 150–200 NPs.

High Resolution Mass Spectrometry

HRMS were carried out using Bruker APEX III FT-MS (7 T magnet), MAT 95 (Finnigan), Thermo Scientific LTQ-FT or Thermo Scientific Exactive.

N_2 Adsorption and Brunauer-Emmett-Teller Surface Area Determination

N_2 adsorption measurements were performed on a Quadrasorb SI from Quantachrom Instruments. BET surface areas were determined using a QuadraSorb station (7.01).

Transmission Fourier Transform Infrared Spectroscopy

FT-IR was performed using a Thermo Scientific Nicolet™ iS5 Spectrometer equipped with a transmission cell. Sample preparation was performed in a glovebox, where a small amount of [Pd-NHC] complex/catalyst was sandwiched between two layers of dry KBr and pressed into a thin disc. For the CO adsorption measurement, 15 mg of each catalyst was taken in a fisher porter bottle and pressurized with CO (3 bar) for 18 h. Then, CO was evacuated under vacuum and FTIR was recorded by preparing KBr pellets of the samples inside the glovebox (iD1 transmission mode).

X-ray Fluorescence

XRF was performed using a spectro Xepos C with a prolene foil of 12 µm at 3keV to 19 keV.

X-ray Photoelectron Spectroscopy

XPS measurements were performed on Ru@SiO_2-[Pd-NHC] to identify the electronic structure of the material. [Pd-NHC] and Ru@SiO_2-[Pd-NHC] after catalysis were also analyzed in a similar manner for comparison. In all cases, the corresponding powder samples were spread onto a carbon tape on the sample holder in a compact manner. XPS measurements were conducted employing a near ambient pressure (NAP) XPS (SPECS GmbH) at ultra-high vacuum conditions (~10^{-8} mbar). The system was equipped with an Al-Kα source which produces monochromated X-rays of energy 1486.6 eV, and a NAP hemispherical energy analyzer with an inbuilt double delay line detector. All high-resolution spectra were recorded using a pass energy of 20 eV and a resolution of 0.05 eV whereas the survey scans were recorded using 100 eV pass energy. In each case, at least 10 consecutive scans were performed. However, a greater number of scans in the Ru@SiO_2-[Pd-NHC] led to the decomposition of the complex and thus detecting metallic Pd. Hence, only the first 3 scans were considered to obtain the high-resolution spectra of Pd3d, N1s and C1s & Ru3d, and the integrated data is presented. Data analysis was performed using the CasaXPS software

after a binding energy calibration using the C1s at 484.6 eV. Inductively Coupled Plasma Optical Emission Spectroscopy (ICP-OES) was carried out by Mikroanalytisches Labor Kolbe on an ICP-OES Spectro Arcos from Spectro. The sample preparation was performed using a CEM-Mars 6 microwave.

4. Synthesis of Ru@SiO_2-[Pd-NHC]

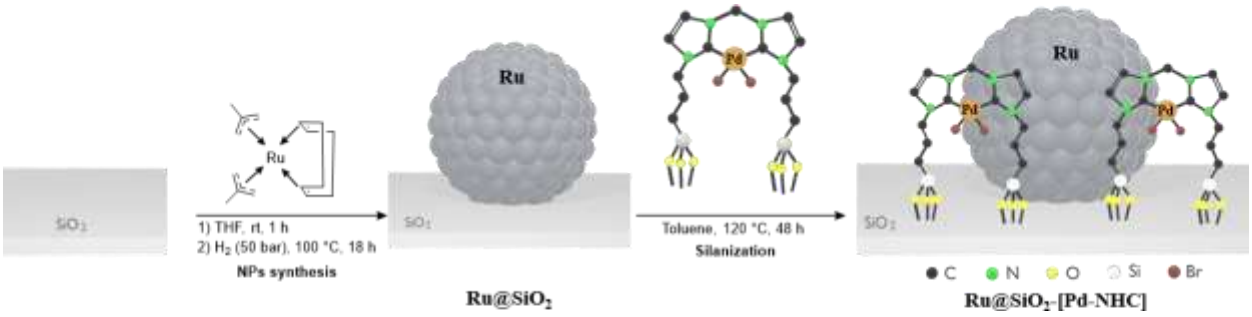

The synthesis of Ru@SiO_2-[Pd-NHC] catalyst was accomplished in three main steps, detailed as follows.

4.1. Synthesis of Ru@SiO_2

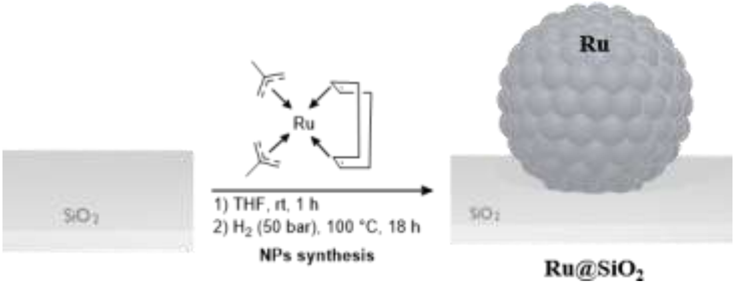

[Ru(2-methylallyl)$_2$(cod)] (64 mg, 0.2 mmol) in THF (5 mL) was added to a suspension of partially dehydroxylated (500 °C under vacuum for 16 h) SiO_2 (1.0 g) in THF (10 mL) and stirred for 1 h at room temperature.[4] After removal of solvent under vacuum, the impregnated SiO_2 was transferred to a 10 mL high-pressure autoclave containing a glass inlet and heated at 100 °C under an atmosphere of H_2 (50 bar) for 18 h. The autoclave was then cooled and Ru@SiO_2 was obtained as a black powder with a theoretical Ru loading of 0.2 mmol.g^{-1} (2 wt%).

4.2. Synthesis of [Pd-NHC] complex [(3,3′-methylene bis(1-(3-triethoxysilyl)propyl)-2,2′-3,3′-tetrahydro-1H-imidazol-2-yl)palladium (II)dibromide]

- Synthesis of NHC.Br ligand [(3,3′-methylene bis(1-(3-triethoxysilyl)propyl)-1H-imidazol-3-ium)dibromide] (**9**)

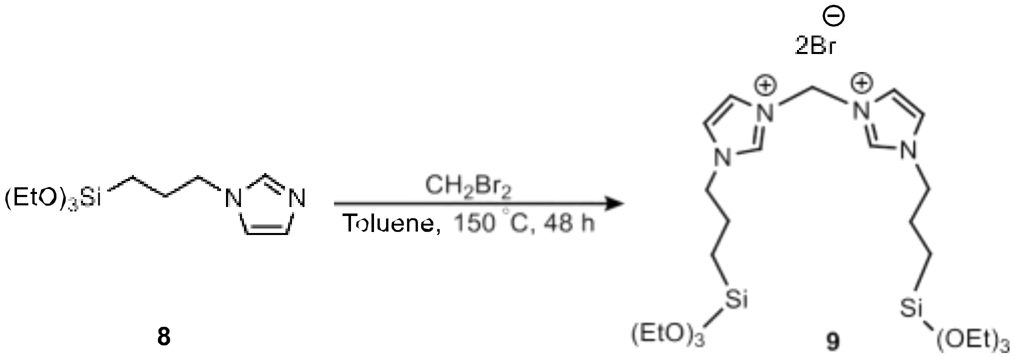

8 **9**

In a schlenk flask, a mixture of **7** [1-(3-triethoxysilylpropyl)-*1H*-imidazole][5] (2.179 g, 8 mmol) and dibromomethane (730 mg, 4.2 mmol) in toluene (10 mL) was refluxed at 150 °C for 48 h. The resulting white precipitate was washed with THF/Toluene (1:10) and dried under vacuum to yield NHC.Br (**9**) as a white powder (4.99 g, 87% isolated yield).

^{1}H NMR (500 MHz, THF-*d*$_8$): δ (ppm) = 11.06 (s, 2H), 9.11 (s, 2H), 8.07 (s, 2H), 7.54 (s, 2H), 4.40 (t, *J* = 7.3 Hz, 4H), 3.83 (q, *J* = 7.0 Hz, 12H), 2.08 – 2.02 (m, 4H), 1.18 (t, *J* = 7.0 Hz, 18H), 0.71 – 0.67 (m, 4H).

^{13}C NMR (126 MHz, THF-*d*$_8$): δ (ppm) = 140.13 (s, 2C), 124.02 (s, 2C), 123.58 (s, 2C), 59.32 (s, 6C), 58.20 (s, 1C), 53.26 (s, 2C), 24.83 (s, 2C), 19.07 (s, 6C), 8.25 (s, 2C).

^{29}Si NMR (79 MHz, THF-*d*$_8$): δ (ppm) = -46.90 (s, 2Si).

- Synthesis of [Pd-NHC] (**9**)

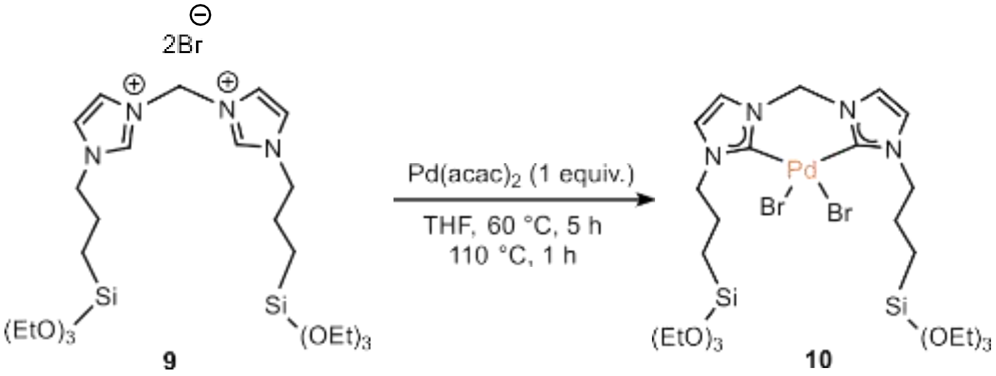

[Pd-NHC] was prepared by adapting a reported procedure.[6] In the glovebox, a schlenk tube was loaded with NHC.Br (**9**, 1.44 g, 2 mmol) and [Pd(acac)$_2$] (0.61 g, 2 mmol) in toluene (10 mL). The clear solution was heated at 60 °C for 5 h, and then 110 °C for 1 h. After completion, the resulting solution was filtered through a pad of celite and concentrated under vacuum. To the crude product, pentane (3*10 mL) was added in a dropwise manner while stirring to obtain the complex **9** as a pale-yellow solid (1.38 g, 83% isolated yield). Notably, diastereotopic protons are generated in complex **10** upon coordination of Pd to the symmetrical ligand **9** as shown in Figure S23. HRMS results showed that **10** exist as an ionic complex with one bromine ligand in the outer coordination sphere.

^{1}H NMR (500 MHz, THF-*d*$_8$) δ (ppm) = 7.74 (d, *J* = 2.0 Hz, 2H), 7.08 (d, *J* = 2.0 Hz, 2H), 6.70 (d, *J* = 13.2 Hz, 1H), 6.33 (d, *J* = 13.1 Hz, 1H), 4.58 (ddd, *J* = 13.0, 9.5, 6.0 Hz, 2H), 4.37 (ddd, *J* = 13.0, 9.7, 5.7 Hz, 2H), 3.81 (q, *J* = 7.0 Hz, 12H), 2.07 – 1.92 (m, 4H), 1.18 (t, *J* = 7.0 Hz, 18H), 0.67 (ddd, *J* = 14.9, 11.0, 5.3 Hz, 2H), 0.54 (ddd, *J* = 14.9, 11.2, 5.6 Hz, 2H).

^{13}C NMR (126 MHz, THF-*d*$_8$) δ (ppm) = 162.48 (s, 2C), 122.36 (s, 2C), 121.67 (s, 2C), 64.38 (s, 1C), 59.16 (s, 6C), 54.31 (s, 2C), 25.98 (s, 2C), 19.00 (s, 6C), 8.12 (s, 2C).

^{29}Si NMR (79 MHz, THF-*d*$_8$): δ (ppm) = -46.56 (s, 2Si).

HRMS/ESI(+) (CH_3CN): m/z = [$C_{25}H_{48}BrN_4O_6PdSi_2$]$^+$ = 741.1327 and [Br]$^-$ = 80

4.3. Synthesis of Ru@SiO_2[Pd-NHC]

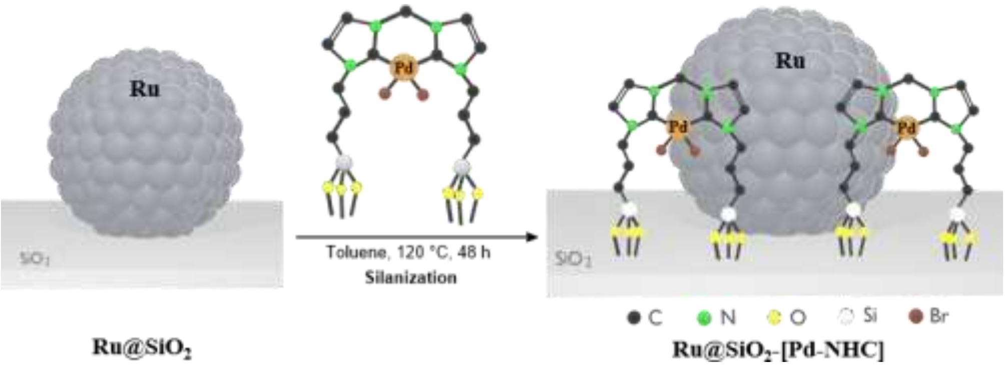

In a 250 mL schlenk flask, a solution of [Pd-NHC] (**10**, 165 mg, 0.2 mmol) was added to a suspension of Ru@SiO_2 (1 g) in THF (10 mL) and refluxed at 120 °C under argon atmosphere for 48 h. After cooling down, the clear supernatant was removed and the solid catalyst was washed with THF (3*10 mL) under vigorous stirring (700 rpm) to remove the loosely bound or physisorbed complex from the surface. Finally, the catalyst was dried and collected as a black powder with a theoretical Pd loading of 0.2 mmol.g^{-1} (2 wt%).

5. General Procedure for the Synthesis of Chalcones

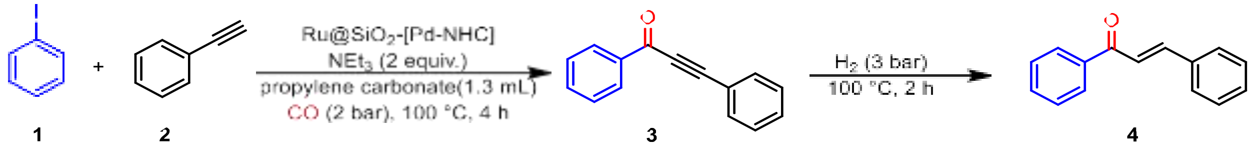

In a glovebox, Ru@SiO_2-[Pd-NHC] (100 mg, 0.04 mmol total metal loading), aryl iodide (1 mmol, 50 equiv. w.r.t. Pd), arylalkyne (1 mmol), NEt_3 (2 mmol), propylene carbonate (1.3 mL) were added to a Fisher-Porter (FP) bottle equipped with a stirring bar. The reaction vessel was sealed and taken out of the glovebox. The reaction mixture was cooled using liquid nitrogen, evacuated and pressurized with 2 bar of CO gas. The FP bottle was then placed behind a protective blast shield and heated at 100 °C for 4 h. Upon cooling to room temperature, the excess pressure was vented under quick vacuum. In case of volatile compounds, the reaction mixture was frozen using liquid nitrogen before depressurizing under vacuum. The FP bottle was then pressurized with 3 bar of H_2 gas and heated at 100 °C for 2 h while kept behind a protective blast shield. After reaction completion, the bottle was cooled down and excess pressure was released carefully. The reaction

mixture was separated by filtration, rinsed with ethyl acetate (EtOAc) and washed with brine. The organic layer was collected, dried over $MgSO_4$ and concentrated by rotary evaporation. The resulting crude product was purified by flash column chromatography on silica gel using EtOAc/pentane as eluent to obtain pure chalcones **4a-4ak.**

6. Time profiles for hydrogenation of 3

Condition (a) using pristine catalyst under H_2: In a glovebox, Ru@SiO_2-[Pd-NHC] (10 mg, 0.004 mmol total metal loading), 1,3-Diphenylprop-2-yn-1-one **3** (103 mg, 0.5 mmol, 125 eq. w.r.t total metal loading), propylene carbonate (1.3 mL) were added to a Fisher-Porter (FP) bottle equipped with a stirring bar. The reaction vessel was sealed and taken out of the glovebox. The bottle was evacuated and pressurized with 2 bar of H_2 gas and heated at 60 °C while kept behind a protective blast shield. After reaction completion, the bottle was cooled down, excess pressure was released carefully and analyzed through GC-FID using mesitylene as internal standard.

Condition (b) using pre-poisoned catalyst under H_2: In a glovebox, Ru@SiO_2-[Pd-NHC] (10 mg, 0.004 mmol total metal loading), 1,3-Diphenylprop-2-yn-1-one **3** (103 mg, 0.5 mmol, 125 eq. w.r.t total metal loading), propylene carbonate (1.3 mL) were added to a Fisher-Porter (FP) bottle equipped with a stirring bar. The reaction vessel was sealed and taken out of the glovebox. The bottle was evacuated and pressurized with 2 bar of CO, placed behind a protective blast shield and heated at 100 °C for 2 h. Upon cooling to room temperature, the excess pressure was vented under quick vacuum. The FP bottle was then pressurized with 2 bar of H_2 gas and heated at 60 °C while kept behind a protective blast shield. After reaction completion, the bottle was cooled down, excess pressure was released carefully and analyzed through GC-FID using mesitylene as internal standard.

Condition (c) using pristine catalyst under H_2/CO: In a glovebox, Ru@SiO_2-[Pd-NHC] (10 mg, 0.004 mmol total metal loading), 1,3-Diphenylprop-2-yn-1-one **3** (103 mg, 0.5 mmol, 125 eq. w.r.t total metal loading), propylene carbonate (1.3 mL) were added to a Fisher-Porter (FP) bottle equipped with a stirring bar. The reaction vessel was sealed and taken out of the glovebox. The bottle was evacuated and pressurized with 0.5 bar of CO and 2 bar of H_2 gas and heated at 60 °C while kept behind a protective blast shield. After reaction completion, the bottle was cooled down, excess pressure was released carefully and analyzed through GC-FID using mesitylene as internal standard.

7. Gram-scale Synthesis

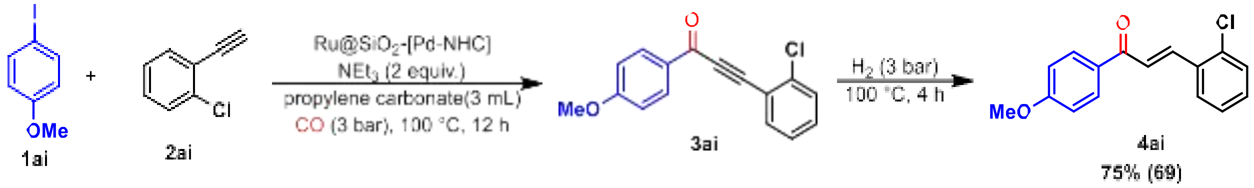

In a glovebox, Ru@SiO_2-[Pd-NHC] (150 mg, 0.06 mmol total metal loading), 4-iodoanisole (6 mmol, 200 equiv. w.r.t. Pd), 1-chloro-2-ethynylbenzene (6 mmol), NEt_3 (12 mmol), propylene carbonate (3 mL) were added to a Fisher-Porter (FP) bottle with a stirring bar. The reaction vessel was sealed and taken out of the glovebox. The reaction mixture was cooled using liquid nitrogen, evacuated and pressurized with 2 bar of CO gas. The FP bottle was then placed behind a protective blast shield and heated at 100 °C for 12 h. After 1 h, there was a pressure drop from 2 to 1 bar, so re-pressurized the bottle to 2 bar. Upon cooling to room temperature, the excess pressure was vented under quick vacuum. The reactor was then pressurized with 3 bar of H_2 gas and heated at 100 °C for 6 h kept behind a protective blast shield. After reaction completion, the bottle was cooled down and excess pressure was released carefully. Conversion and yield were determined by GC-FID using mesitylene as internal standard. The reaction mixture was separated by filtration, rinsed with ethyl acetate (EtOAc) and washed with brine. The organic layer was collected, dried over $MgSO_4$ and concentrated by rotary evaporation. The resulting crude product was purified by flash column chromatography on silica gel using EtOAc/pentane as eluent to obtain pure target compound **4ai** in 69% isolated yield (1.13 g).

^{1}H NMR (400 MHz, Chloroform-*d*) δ (ppm) = 8.16 (d, *J* = 15.7 Hz, 1H), 8.05 – 8.02 (m, 2H), 7.75 – 7.73 (m, 1H), 7.49 (d, *J* = 15.7 Hz, 1H), 7.45 – 7.42 (m, 1H), 7.34 – 7.30 (m, 2H), 7.00 – 6.97 (m, 2H), 3.91 (s, 3H).

^{13}C NMR (101 MHz, Chloroform-*d*) δ (ppm) = 188.72, 163.65, 139.88, 135.49, 133.56, 131.09, 131.07, 130.92, 130.38, 127.87, 127.16, 124.80, 114.00, 55.62.

XRF analysis: I (53 ppm), Ru (< 0.2 ppm), Pd (< 1.4 ppm).

8. Catalyst Recycling

i) Recycling under adapted conditions: In a glovebox, Ru@SiO_2-[Pd-NHC] (100 mg, 0.04 mmol total metal loading), aryl iodide (1 mmol, 50 equiv. w.r.t. Pd), arylalkyne (1 mmol), NEt_3 (2 mmol),

propylene carbonate (1.3 mL) were added to a Fisher-Porter (FP) bottle with a stirring bar. The reaction vessel was sealed and taken out of the glovebox. The reaction mixture was cooled using liquid nitrogen, evacuated and pressurized with 2 bar of CO gas. The FP bottle was then placed behind a protective blast shield and heated at 100 °C for 4 h. Upon cooling to room temperature, the excess pressure was vented through quick vacuum. The FP bottle was then pressurized with 1 bar of H_2 gas and heated at 100 °C for 1 h kept behind a protective blast shield. After reaction completion, the bottle was cooled down and excess pressure was released under vacuum. The bottle was taken inside the glovebox and the supernatant was removed to prepare a GC sample. The solid catalyst was washed with propylene carbonate (1.5 mL). New substrates and solvent were added to the washed catalyst to proceed the next cycle.

ii) Recycling under standard optimized conditions: In a glovebox, Ru@SiO_2-[Pd-NHC] (100 mg, 0.04 mmol total metal loading), aryl iodide (1 mmol, 50 equiv. w.r.t. Pd), arylalkyne (1 mmol), NEt_3 (2 mmol), propylene carbonate (1.3 mL) were added to a Fisher-Porter (FP) bottle with a stirring bar. The reaction vessel was sealed and taken out of the glovebox. The reaction mixture was cooled using liquid nitrogen, evacuated and pressurized with 2 bar of CO gas. The FP bottle was then placed behind a protective blast shield and heated at 100 °C for 4 h. Upon cooling to room temperature, the excess pressure was vented through quick vacuum. The FP bottle was then pressurized with 3 bar of H_2 gas and heated at 100 °C for 2 h kept behind a protective blast shield. After reaction completion, the bottle was cooled down and excess pressure was released under vacuum. The bottle was taken inside the glovebox and the supernatant was removed to prepare a GC sample. The solid catalyst was washed with propylene carbonate (1.5 mL). New substrates and solvent were added to the washed catalyst to proceed the next cycle. For catalyst regeneration: After 8th cycle, catalyst was washed with acetonitrile (1 ml x3) and heated at 60 °C for 2 h under high vacuum.

9. Supplementary Tables and Figures

9.1. Catalyst Characterization

Table S1. Characterization of different materials by N_2 physisorption and ICP-OES.

Entry	Material	BET surface area ($m^2.g^{-1}$)	Metal loading (ICP-OES) (wt%)	
			Ru	Pd
1	SiO_2	332	-	-
2	Ru@SiO_2	325	1.89	-
3	Ru@SiO_2-[Pd-NHC]	289	1.86	1.97
4	After catalysis	297	1.72	1.89

Table S2. Overview of representative experimental parameters of ^1H-^{13}C CP MAS NMR measurements.

	Ru@SiO_2-[Pd-NHC] before catalysis	Ru@SiO_2-[Pd-NHC] after catalysis	SiO_2-[Pd-NHC]	Pd-NHC
MAS frequency / kHz	17.0	17.0	17.0	17.0
B_0 / T	11.7	11.7	11.7	11.7
$\nu_1(^1H)$ excitation / kHz	100	100	100	100
CP contact power $\nu_1(^1H)$ / kHz	60	60	60	60
CP contact power $\nu_1(^{13}C)$ / kHz	43	43	43	43
CP contact time / ms	5.0	5.0	5.0	5.0
FID acquisition time / ms	19.7	14.7	15.4	15.4
Interscan delay / s	1.95	2.50	1.95	12.5
Number of scans	27000	27000	30720	2048
Probe target temperature / K	280	270	270	270
Total acquisition time/ h	14.8	18.9	16.9	7.2

Table S3. Overview of experimental parameters of ^{1}H-^{29}Si CP-MAS NMR measurements.

	Ru@SiO_2-[Pd-NHC] before catalysis	Ru@SiO_2-[Pd-NHC] after catalysis	NHC.Br
MAS frequency / kHz	17.0	17.0	17.0
B_0 / T	16.4	11.7	11.7
$\nu_1(^{1}H)$ excitation / kHz	100	100	100
CP contact power ν_1 (^{1}H) / kHz	60	60	60
CP contact power $\nu_1(^{29}Si)$ / kHz	43	43	43
CP contact time / ms	5.0	4.5	4.5
FID acquisition time / ms	14.7	15.4	41.5
Interscan delay / s	1.69	1.04	3.25
Number of scans	40000	72000	256
Probe target temperature / K	280	270	270
Total acquisition time/ h	19.0	21.3	0.25

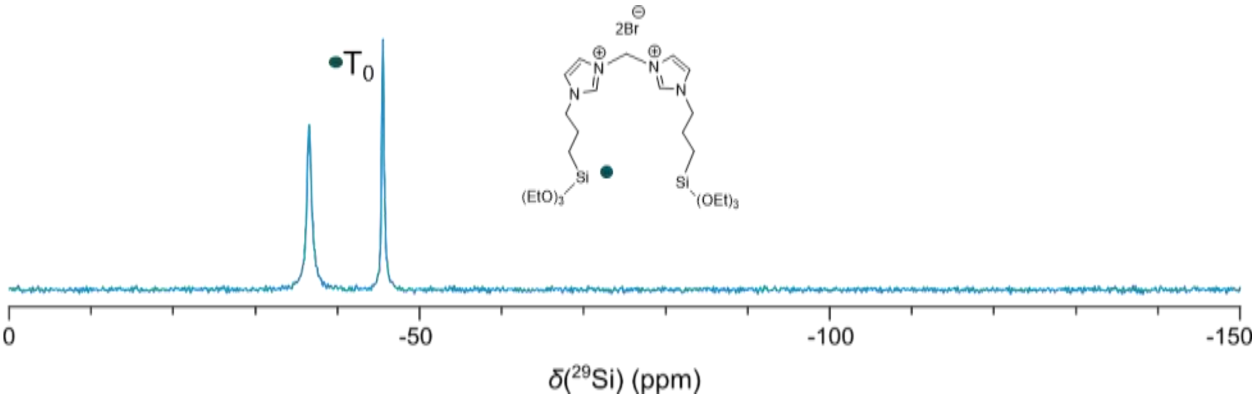

Figure S1 ^{1}H-^{29}Si CP-MAS NMR spectrum of non-grafted NHC.Br recorded at 16.4 T and 17.0 kHz MAS.

Table S4. Assignment of decomposed signals in Figure 3a and S1 to different silicate species.

δ_{iso} (ppm)	Assignment	Structure
-47	T_0	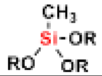
-53	T_1	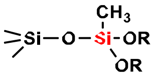
-58	T_2	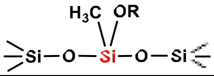
-95	Q_2	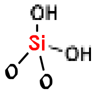
-101	Q_3	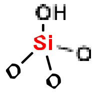
-110	Q_4	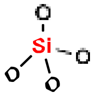

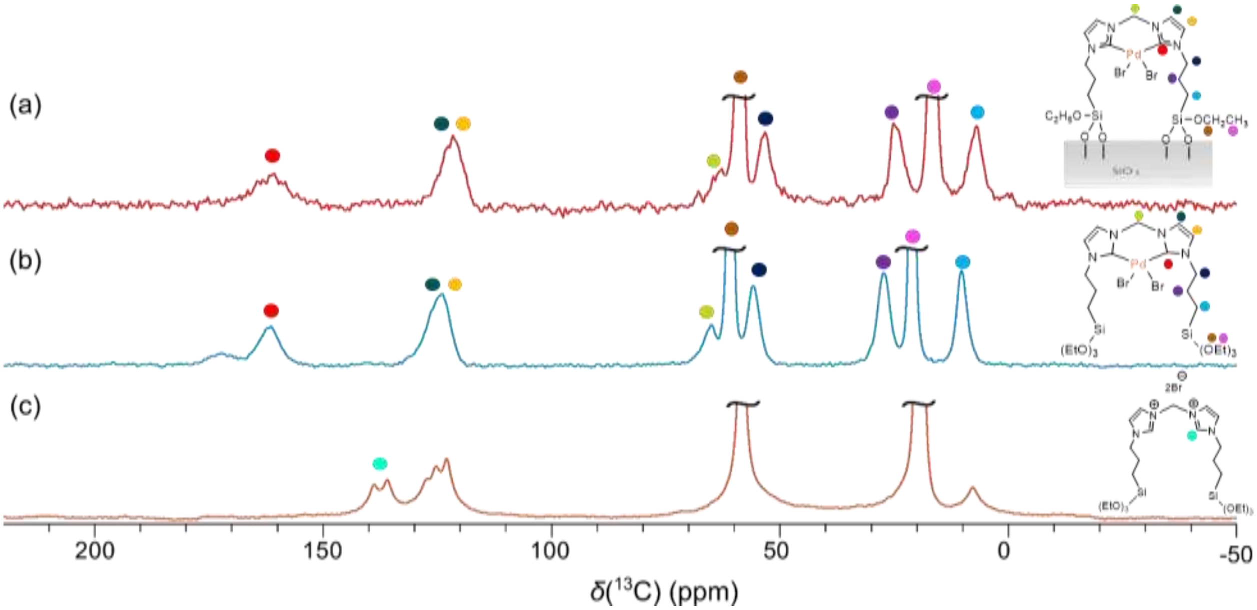

Figure S2. ^{1}H-^{13}C CP-MAS NMR spectra recorded at 11.7 T and 17.0 kHz MAS. a) SiO_2-[Pd-NHC], b) Pd-NHC, c) non-grafted NHC.Br. Peak splittings in c are caused by different chemical environments in the asymmetric unit.

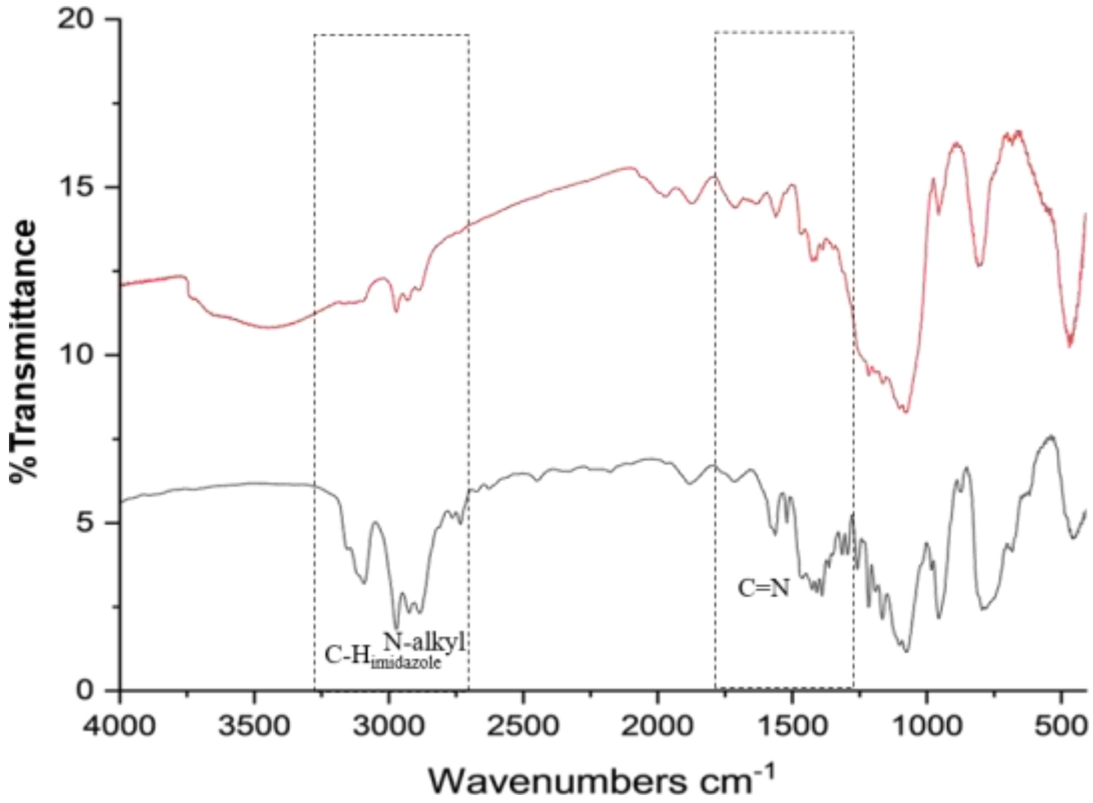

Figure S3. Transmission FT-IR spectra of [Pd-NHC] (black) and Ru@SiO_2-[Pd-NHC] (red).

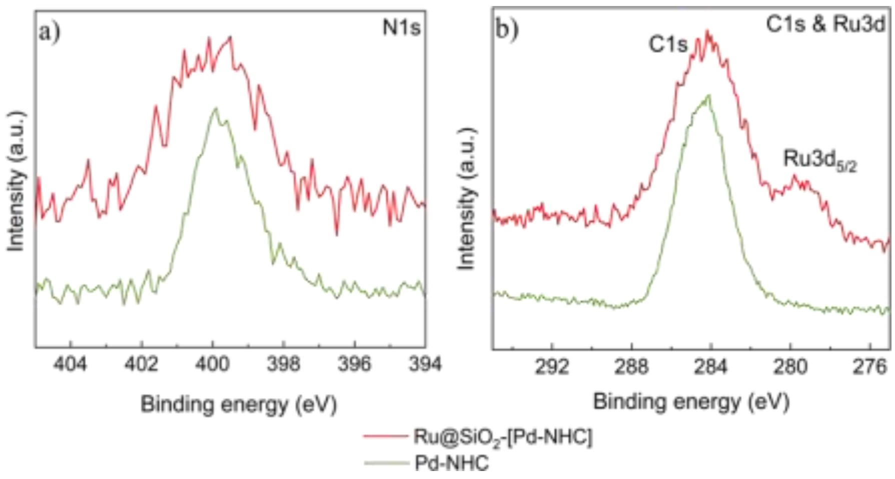

Figure S4: Characterization of Ru@SiO_2-[Pd-NHC] before catalysis by XPS. High resolution XPS spectra of (a) C1s & Ru3d, (b) N1s.

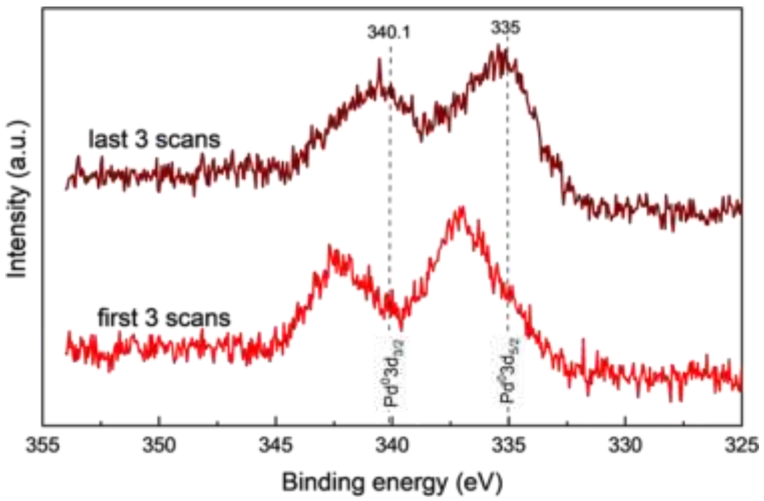

Figure S5. XPS high resolution scans showing the Pd3d region from the first and last 3 scans of Ru@SiO_2-[Pd-NHC] catalyst. The binding energy positions of $3d_{5/2}$ and $3d_{3/2}$ in metallic Pd is marked for reference.

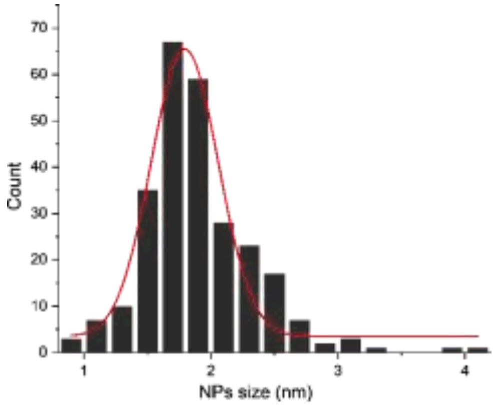

Figure S6. Size distribution of Ru nanoparticles in Ru@SiO_2-[Pd-NHC]. Average diameter = 1.8 ± 0.4 nm.

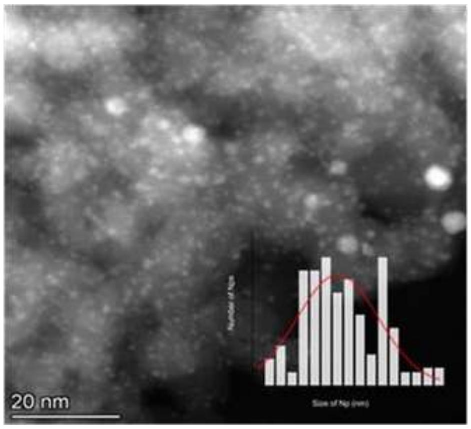

Figure S7. NPs formation via beam damage during the characterization of reference Pd-NHC@SiO_2 by STEM-HAADF. Average diameter = 1.3 ± 0.3 nm.

9.2. Catalytic Study

Testing of catalysts and reaction parameters for carbonylative sonogashira coupling

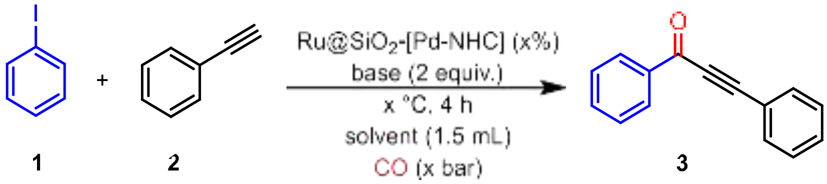

Table S5. Testing of catalysts.

Entry	Catalyst	Conv. (%)[a]	Yield **3** (%)[a]
1	[Pd-NHC]	74	72
2	5% Pd/C	12	11
3	Ru@SiO_2	2	2
4	Ru@SiO_2-[Pd-NHC]	29	27
5	$Pd(acac)_2$	68	66

Reaction conditions: (Catalyst: 0.006 mmol of Pd/Ru), iodobenzene (0.3 mmol; 50 equiv. w.r.t to catalyst), phenylacetylene (0.3 mmol), NEt_3 (0.6 mmol), toluene (1.5 mL), CO (4 bar), 100 °C, 4 h; [a]Conv. and yield were determined through GC-FID using mesitylene as internal standard. Byproducts are 1,2-diphenylethyne, 1,4-diphenylbuta-1,3-diyne and benzene.

Table S6. Testing of bases using Ru@SiO_2-[Pd-NHC].

Entry	Base	Conv. (%)[a]	Yield **3** (%)[a]
1	NEt_3	29	27
2	K_2CO_3	8	7
3	DIPEA	13	12

Reaction conditions: (Ru@SiO_2-[Pd-NHC]: 30 mg; 0.012 mmol total metal loading), iodobenzene (0.3 mmol; 50 equiv. w.r.t to [Pd-NHC]), phenylacetylene (0.3 mmol), Base (0.6 mmol), toluene (1.5 mL), CO (4 bar), 100 °C, 4 h; [a]Conv. and yield were determined through GC-FID using mesitylene as internal standard. Byproducts are 1,2-diphenylethyne, 1,4-diphenylbuta-1,3-diyne and benzene.

Table S7. Testing of solvents using Ru@SiO_2-[Pd-NHC].

Entry	Solvent	Conv. (%)[a]	Yield **3** (%)[a]
1	Toluene	29	27
2	DMC	33	32
3	Propylene carbonate	74	72
4	DMF	86	79
5	1,4-Dioxane	50	49

Reaction conditions: (Ru@SiO_2-[Pd-NHC]: 30 mg; 0.012 mmol total metal loading), iodobenzene (0.3 mmol; 50 equiv. w.r.t to [Pd-NHC]), phenylacetylene (0.3 mmol), NEt_3 (0.6 mmol), solvent (1.5 mL), CO (4 bar), 100 °C, 4 h; [a]Conv. and yield were determined through GC-FID using mesitylene as internal standard. Byproducts are 1,2-diphenylethyne, 1,4-diphenylbuta-1,3-diyne and benzene.

Table S8. Testing of CO pressures using Ru@SiO_2-[Pd-NHC].

Entry	CO Pressure	Conv. (%)[a]	Yield **3** (%)[a]
1	1 bar	79	76
2	2 bar	83	80
3	4 bar	74	72
4	10 bar	98	87

Reaction conditions: (Ru@SiO_2-[Pd-NHC]: 30 mg; 0.012 mmol total metal loading), iodobenzene (0.3 mmol; 50 equiv. w.r.t to [Pd-NHC]), phenylacetylene (0.3 mmol), NEt_3 (0.6 mmol), PC (1.5 mL), 100 °C, 4 h; [a]Conversion and yield were determined through GC-FID using mesitylene as internal standard. Byproducts are 1,2-diphenylethyne, 1,4-diphenylbuta-1,3-diyne and benzene.

Table S9. Testing of catalyst loadings (reflected by Pd loading in mol%) using Ru@SiO_2-[Pd-NHC].

Entry	Catalyst loading	Conv. (%)[a]	Yield **3** (%)[a]
1	0.5 mol%	68	65
2	1 mol%	70	68
3	2 mol%	83	80
4	3 mol%	84	80

Reaction conditions: (Ru@SiO_2-[Pd-NHC]: x mg; x mmol of Pd/Ru), iodobenzene (0.3 mmol; x equiv. w.r.t to [Pd-NHC]), phenylacetylene (0.3 mmol), NEt_3 (0.6 mmol), PC (1.5 mL), CO (2 bar), 100 °C, 4 h; [a]Conversion and yield were determined through GC-FID using mesitylene as internal standard. Byproducts are 1,2-diphenylethyne, 1,4-diphenylbuta-1,3-diyne and benzene.

Table S10. Testing of reaction temperatures using Ru@SiO_2-[Pd-NHC].

Entry	Temperature	Conv. (%)[a]	Yield **3** (%)[a]
1	80 °C	50	47
2	100 °C	83	80
3	120 °C	87	81

Reaction conditions: (Ru@SiO_2-[Pd-NHC]: 30 mg; 0.012 mmol total metal loading) iodobenzene (0.3 mmol; 50 equiv. w.r.t to [Pd-NHC]), phenylacetylene (0.3 mmol), NEt_3 (0.6 mmol), PC (1.5 mL), CO (2 bar), 4 h; [a]Conv. and yield were determined through GC-FID using mesitylene as internal standard. Byproducts are 1,2-diphenylethyne, 1,4-diphenylbuta-1,3-diyne and benzene.

Table S11. Testing of reaction scale and substrate concentrations using Ru@SiO_2-[Pd-NHC].

Entry	[**1**] (mmol)	Time (h)	**1**:Pd molar ratio	Conv. (%)[a]	Yield **3** (%)[a]
1	0.3	1	50	51	49
2	0.3	4	50	75	73
3	1	1	50	71	69
4	1	4	50	92	89
5	1	1	333	52	50
6	1	1	500	58	56
7	1	4	500	83	81

Reaction conditions: (Ru@SiO_2-[Pd-NHC], iodobenzene (x mmol), phenylacetylene (1 equiv.), NEt_3 (2 equiv.), PC (1.5 mL), CO (2 bar); [a]Conversion and yield were determined through GC-FID using mesitylene as internal standard. Byproducts are 1,2-diphenylethyne, 1,4-diphenylbuta-1,3-diyne and benzene.

Table S12. Hot-filtration experiment using Ru@SiO_2-[Pd-NHC].

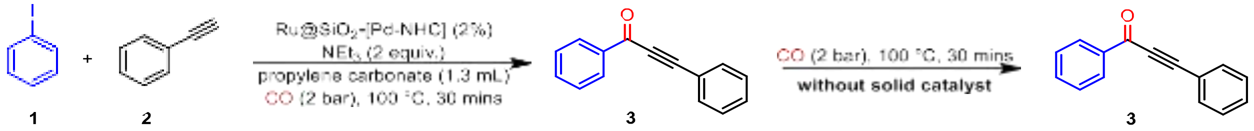

Entry	Time (mins)	Conversion (%)[a]	Yield (%)[a]
1	30	29	27
2	30 (after hot filtration)	32	30

Reaction conditions: Ru@SiO_2-[Pd-NHC] (100 mg; 0.04 mmol total metal loading) Iodobenzene (1 mmol; 50 equiv. w.r.t to Pd-NHC), Phenylacetylene (1 mmol), NEt_3 (2 mmol), PC (1.3 mL), CO (2 bar), 100 °C, 30 mins; After 30 mins, the hot solution was filtered through a pad of celite and analysed. The filtered crude solution was then performed with CO (2 bar) at 100 °C for 30 mins without solid catalyst. [a]Conversion and Yield was determined through GC-FID using mesitylene as internal standard.

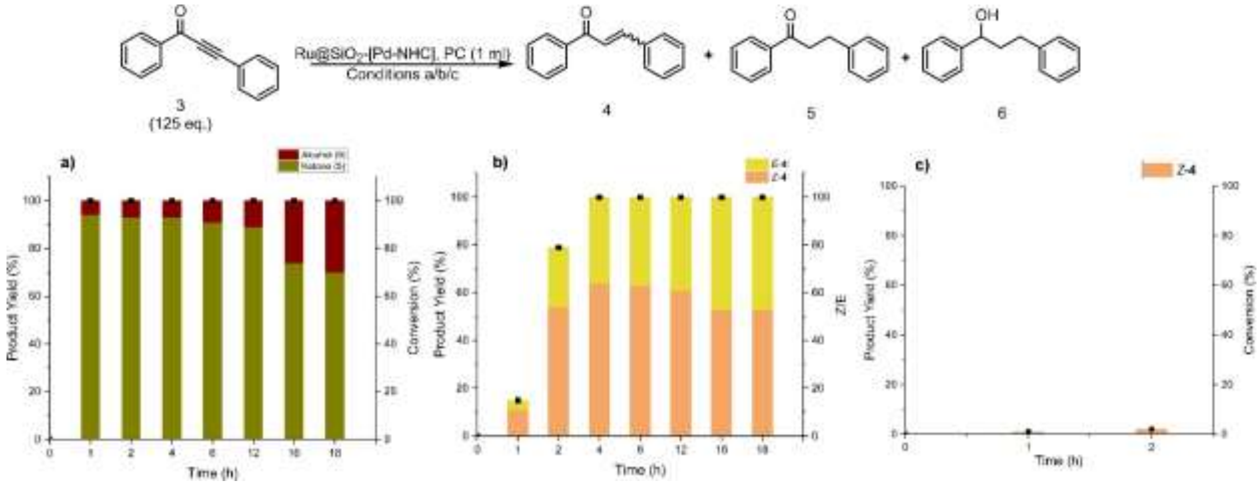

Figure S8. Time profile of the hydrogenation of **3** using a) pristine Ru@SiO_2-[Pd-NHC] under H_2; b) pre-poisoned Ru@SiO_2-[Pd-NHC] under H_2; c) pristine Ru@SiO_2-[Pd-NHC] under H_2/CO. Reaction conditions: **a)** Catalyst (10 mg), substrate **3** (103 mg, 0.5 mmol, 125 eq. w.r.t total metal loading), PC (1 ml), H_2 (2 bar), 60 °C. **b)** Catalyst (10 mg), substrate **3** (103 mg, 0.5 mmol, 125 eq. w.r.t total metal loading), PC (1 ml). The bottle was pressurized with CO (2 bar) and heated at 100 °C for 2 h. CO pressure was released and pressurized with H_2 (2 bar), 60 °C. **c)** Catalyst (10 mg), substrate **3** (103 mg, 0.5 mmol, 125 eq. w.r.t total metal loading), PC (1 ml), CO (0.5 bar), H_2 (2 bar), 60 °C.

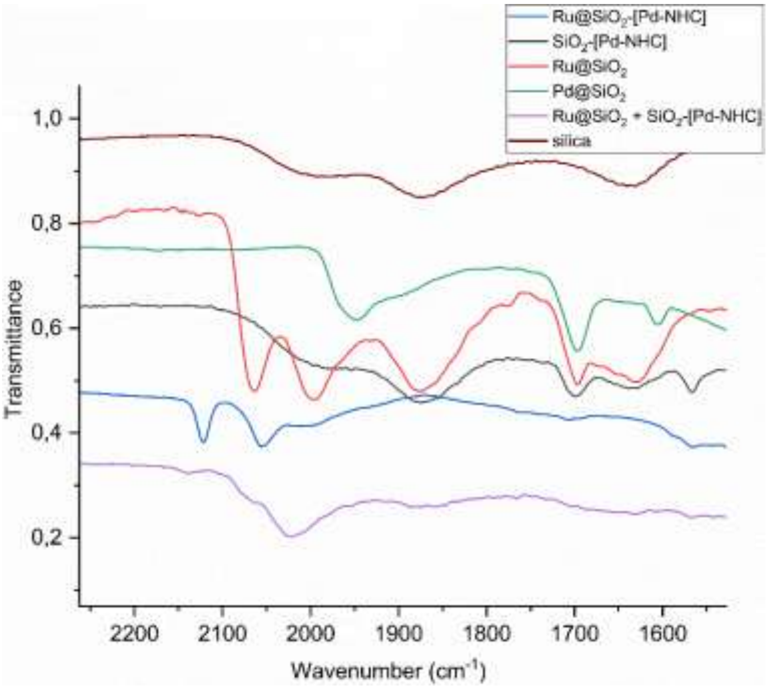

Figure S9. Transmission FT-IR spectra of CO-adsorbed Ru@SiO_2-[Pd-NHC] and reference materials.

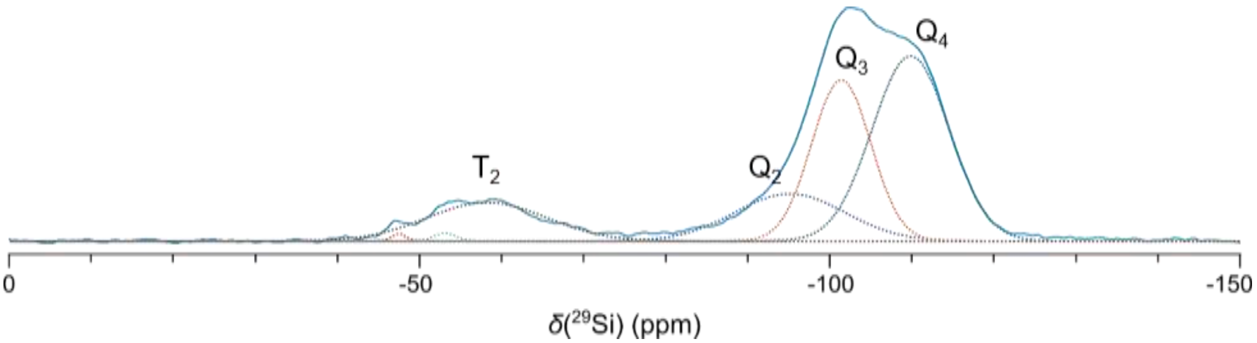

Figure S10. ^{1}H- ^{29}Si CP-MAS NMR spectrum of Ru@SiO_2-[Pd-NHC] after catalysis, recorded at 11.7 T and 17.0 kHz MAS. Dashed lines show the decomposition of the spectrum to individual resonances.

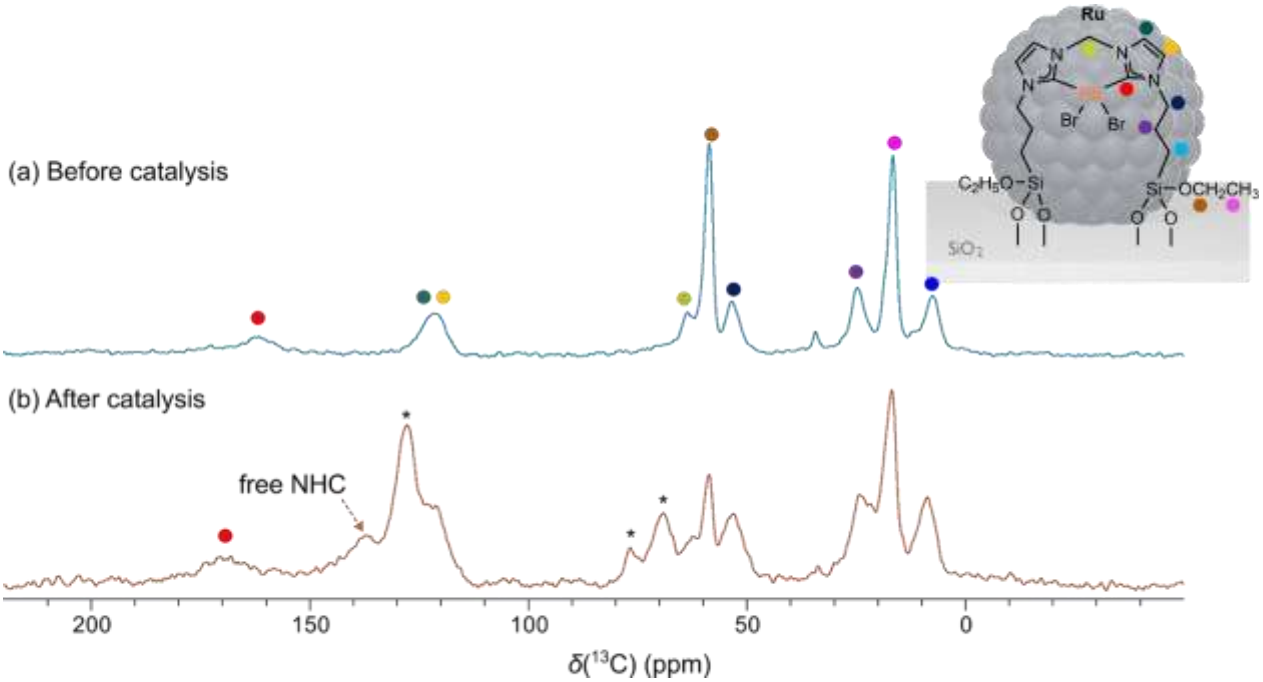

Figure S11. Solid state ^1H-^{13}C CPMAS NMR spectra of Ru@SiO_2-[Pd-NHC] (a) before and (b) after catalysis recorded at 11.7 T and 17.0 kHz MAS. * labels signal from left-over product and solvent.

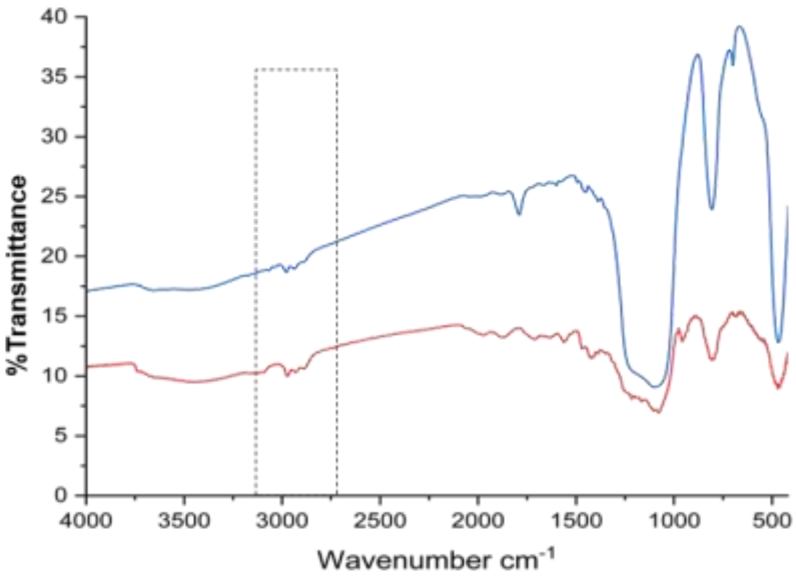

Figure S12. Transmission FT-IR spectra of Ru@SiO_2-[Pd-NHC] before (red) and after catalysis (blue).

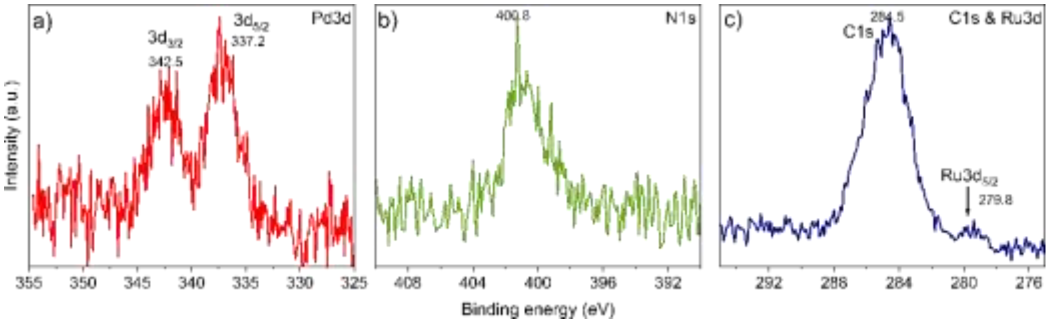

Figure S13: Characterization of Ru@SiO_2-[Pd-NHC] after catalysis by XPS. High resolution XPS spectra of (a) Pd3d, (b) N1s, and (c) C1s & Ru3d.

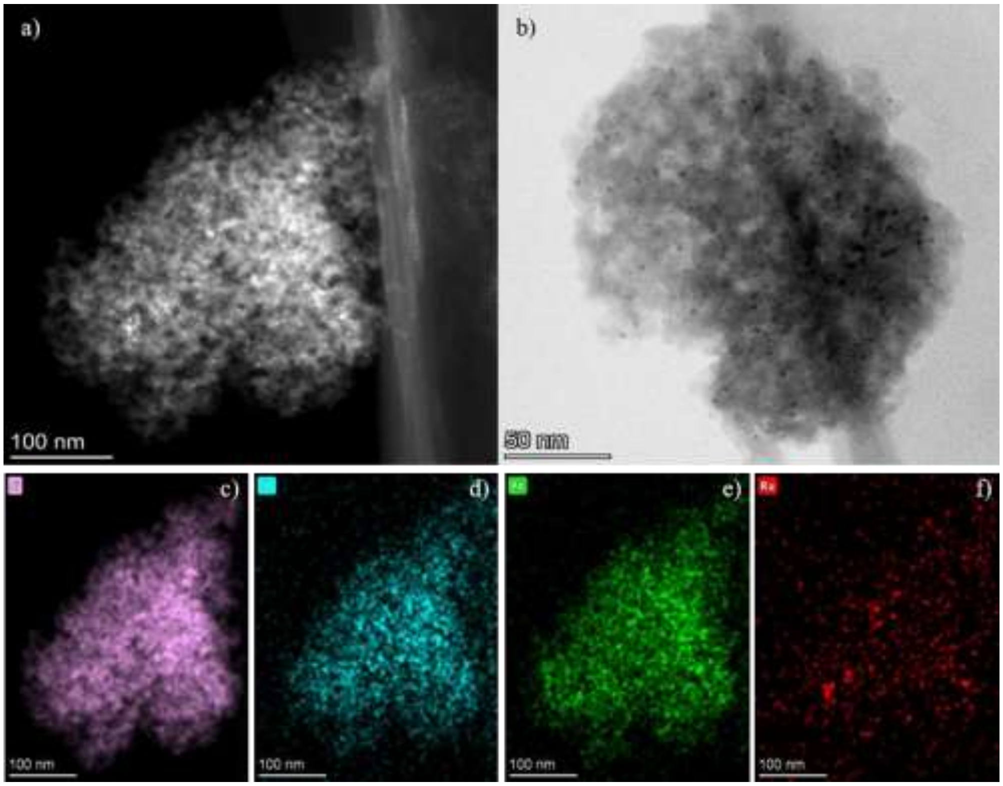

Figure S14. Characterization of the spent Ru@SiO_2-[Pd-NHC] catalyst after four catalytic cycles by electron microscopy a) STEM-HAADF b) STEM-BF images; c-f) EDX elemental mapping of Si-Kα, I-Lα, Pd-Lα, Ru-Kα.

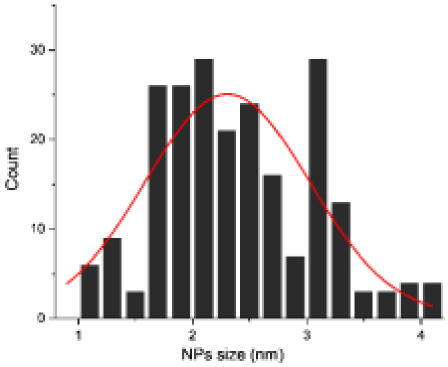

Figure S15. Size distribution of Ru-nanoparticles in Ru@SiO_2-[Pd-NHC] after catalysis (4 runs) of average diameter = 2.4 ± 0.7 nm.

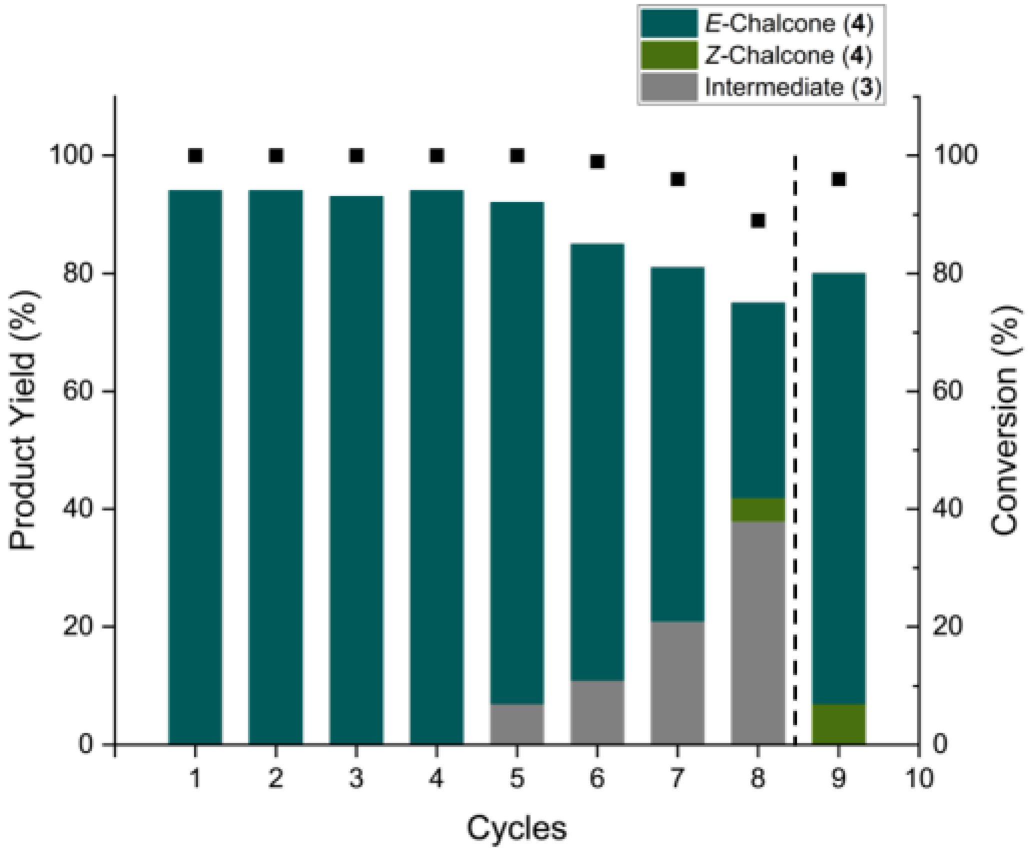

Figure S16. Recycling experiments under optimized conditions. Reaction conditions: Step 1: Ru@SiO_2-[Pd-NHC] (100 mg, 0.04 mmol total metal loading), iodobenzene (1 mmol, 50 equiv. w.r.t. Pd-NHC), phenylacetylene (1 mmol), NEt_3 (2 mmol), PC (1.5 mL), CO (2 bar), 100 ˚C, 4 h. Excess CO pressure was released. Step 2: H_2 (3 bar), 100 ˚C, 2 h. Conversion and yield were determined through GC-FID using mesitylene as internal standard. Catalyst was washed with propylene carbonate (2 ml) between each cycle. Catalyst re-activation after cycle 8 (see procedure). Byproducts are 1,2-diphenylethyne, 1,4-diphenylbuta-1,3-diyne and benzene. Data for the 9th cycle were obtained after regeneration (washing + drying) of the catalyst.

Table S13. Carbonylative Sonogashira coupling with different coupling partners.

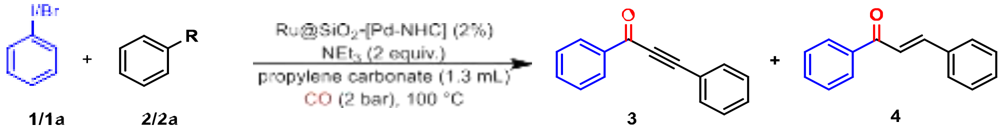

Entry	Aryl halide	Coupling partner	Time (h)	Conversion (%)[a]	Yield (%)[a]	
					**3**	**4**
1	PhBr (**1a**)	phenylacetylene (**2**)	4	2	0	-
2	PhI (**1**)	styrene (**2a**)	18	17	-	10

Reaction conditions: Ru@SiO_2-[Pd-NHC] (100 mg; 0.04 mmol total metal loading) **1** (1 mmol; 50 equiv. w.r.t to Pd-NHC), **2/2a** (1 mmol), NEt_3 (2 mmol), PC (1.3 mL), CO (2 bar), 100 °C; [a]Conversion and Yield was determined through GC-FID using mesitylene as internal standard. Byproducts are 1,2-diphenylethyne, 1,4-diphenylbuta-1,3-diyne and benzene.

10. Synthetic Approach Evaluation

(*E*)-3-Phenyl-1-(thien-2-yl)prop-2-en-1-one (**4o**) was chosen as the target product for this evaluation considering the anti-cancer properties of its derivatives. Our proposed one-pot tandem approach was systematically compared to carbonylative heck coupling (Figure S17). The detailed synthetic routes are illustrated in Figures S18 and S19. In both synthetic approaches, since the starting material is a commercially available compound, its preparation was not included in the evaluation. Five parameters based on green chemistry principles were selected to rank the pathways: the number of steps (Steps), atom economy (AE), overall reaction yield (Y), hazardous nature of the reagents (Safety), and the economic aspect (Eco).

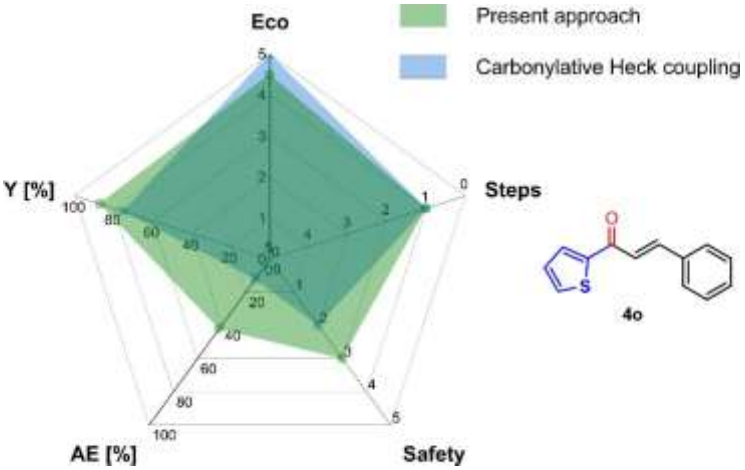

Figure S17. Evaluation of the synthesis of (*E*)-3-phenyl-1-(thien-2-yl)prop-2-en-1-one (**4o**) by a conventional route (carbonylative Heck coupling), and by the presently reported one-pot carbonylative Sonogashira coupling + selective hydrogenation approach. Area proportional to the green chemistry metrics (large area = good metrics).

The AE was determined using the following formula:

$$AE = \frac{\text{total molecular weight of desired product}}{\text{total molecular weight of all reactants}} \times 100$$

The Safety parameter was evaluated qualitatively by ranking the different chemicals on a scale from one (= most hazardous) to five (= least hazardous) based on their hazardous nature (Table S14). The parameter Eco is based on the difference in price between the starting materials and the desired product. The other approach provides a better difference and the Eco parameter is set arbitrarily to 5. For our approach, the addition of value is slightly high, and Eco was thus set to 4.5.

Product yield and number of steps are shown in Figures S20 and S21.

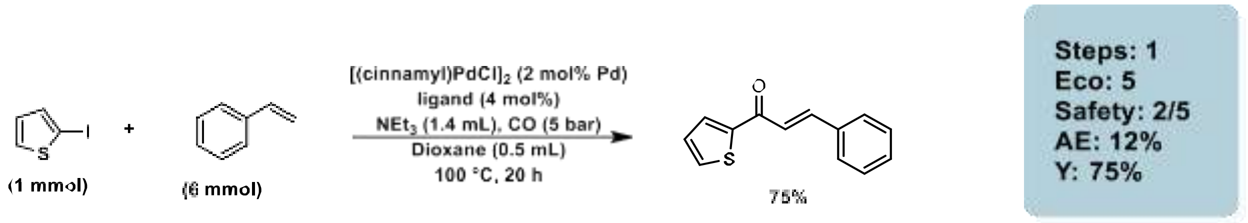

Figure S18. Carbonylative Heck coupling pathway for the preparation of (*E*)-3-Phenyl-1-(thien-2-yl)prop-2-en-1-one.[7]

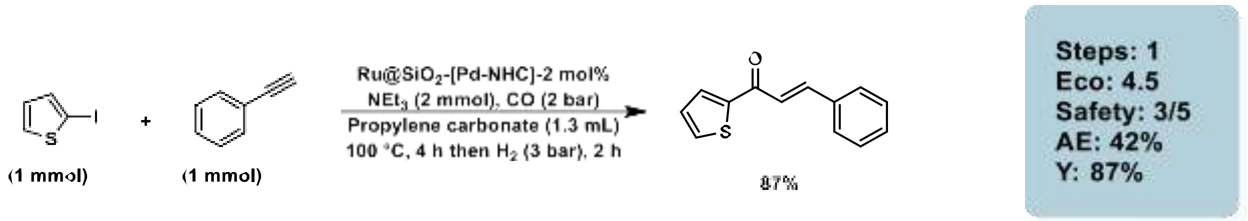

Figure S19. Our synthetic pathway for the preparation of (*E*)-3-Phenyl-1-(thien-2-yl)prop-2-en-1-one.

Table S14. GHS ranking of chemicals.

GHS ranking	
1	explosive, oxidizing, toxic, health hazard
2	harmful, flammable, environmental, corrosive (combination of 3 hazards)
3	harmful, flammable, environmental, corrosive (combination of 2 hazards)
4	harmful, flammable, environmental, corrosive (1 hazard)
5	-

Chemical	CAS	MW (g/mol)	Price (€/g)	GHS hazard
2-iodothiophene	3437-95-4	210.03	3.9	Harmful + corrosive
Styrene	100-42-5	104.15	0.4	Toxic + Flammable
Phenylacetylene	536-74-3	102.13	1.6	Toxic + Flammable
Dioxane	123-91-1	88.11	0.15	Toxic + Flammable + Harmful
Triethylamine	121-44-8	101.19	0.4	Toxic + Corrosive
Propylene carbonate	108-32-7	102.09	0.199	Harmful
(*E*)-3-Phenyl-1-(thien-2-yl)prop-2-en-1-one	3988-77-0	214.29	70.80	product

11. Analytical Data for Isolated Chalcones

(*E*)-1-(4-methoxyphenyl)-3-phenylprop-2-en-1-one (4b) was obtained by following the general

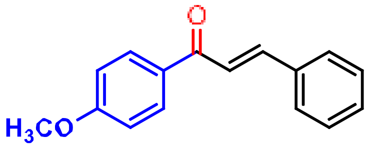

procedure on a 1 mmol scale and isolated by flash column chromatography (EtOAc:pentane = 10:90) as an off-white solid. Yield: 205 mg, 86%. NMR data of the compound is in good agreement with the literature data.[7]

^{1}H NMR (400 MHz, Chloroform-*d*) δ (ppm) = 8.05 (d, *J* = 8.9 Hz, 2H), 7.81 (d, *J* = 15.7 Hz, 1H), 7.66 – 7.64 (m, 2H), 7.55 (d, *J* = 15.7 Hz, 1H), 7.42 (dd, *J* = 5.2, 1.9 Hz, 3H), 6.99 (d, *J* = 8.9 Hz, 2H), 3.89 (s, 3H).

^{13}C NMR (101 MHz, Chloroform-*d*) δ (ppm) = 188.86, 163.57, 144.11, 135.22, 131.23, 130.96, 130.47, 129.06, 128.50, 122.01, 113.98, 55.64.

XRF analysis: I (20 ppm), Ru (0.2 ppm), Pd (0.6 ppm).

(*E*)-1-(3,5-dimethylphenyl)-3-phenylprop-2-en-1-one (4f) was obtained by following the general procedure on a 1 mmol scale and isolated by flash column chromatography (EtOAc:pentane = 30:70) as an yellow oil. Yield: 208 mg, 88%. NMR data of the compound is in good agreement with the literature data.[7]

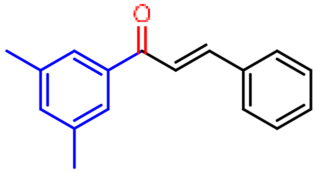

^{1}H NMR (400 MHz, Chloroform-*d*) δ (ppm) = 7.81 (d, *J* = 15.7 Hz, 1H), 7.68 – 7.64 (m, 4H), 7.53 (d, *J* = 15.7 Hz, 1H), 7.45 – 7.39 (m, 3H), 7.22 (s, 1H), 2.41 (s, 6H).

^{13}C NMR (101 MHz, Chloroform-*d*) δ (ppm) = 190.89, 144.50, 138.38, 138.34, 135.05, 134.57, 130.52, 129.01, 128.52, 126.37, 122.43, 21.37.

(*E*)-1-(4-acetylphenyl)-3-phenylprop-2-en-1-one (4j) was obtained by following the general procedure on a 1 mmol scale and isolated by flash column chromatography (EtOAc:pentane = 20:80) as a white solid. Yield: 225 mg, 90%. NMR data of the compound is in good agreement with the literature data.[8]

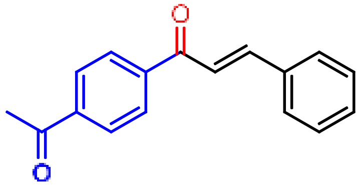

^{1}H NMR (400 MHz, Chloroform-*d*) δ (ppm) = 8.07 (s, 4H), 7.82 (d, *J* = 15.7 Hz, 1H), 7.67 – 7.64 (m, 2H), 7.51 (d, *J* = 15.7 Hz, 1H), 7.44 – 7.43 (m, 3H), 2.66 (s, 3H).

^{13}C NMR (101 MHz, Chloroform-*d*) δ (ppm) = 197.67, 190.16, 146.00, 141.74, 139.96, 134.69, 131.04, 129.17, 128.79, 128.72, 128.65, 121.91, 27.04.

(*E*)-1-(4-Fluorophenyl)-3-phenylprop-2-en-1-one (4k) was obtained by following the general procedure on a 1 mmol scale and isolated by flash column chromatography (EtOAc:pentane = 10:90) as a white solid. Yield: 183 mg, 81%. NMR data of the compound is in good agreement with the literature data.[7]

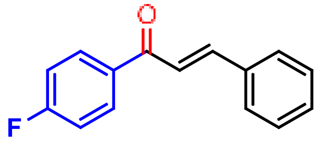

^{1}H NMR (400 MHz, Chloroform-*d*) δ (ppm) = 8.08 – 8.05 (m, 2H), 7.82 (d, *J* = 15.7 Hz, 1H), 7.67-7.63 (m, 2H), 7.51 (d, *J* = 15.7 Hz, 1H), 7.43 (t, *J* = 3.3 Hz, 3H), 7.18 (t, *J* = 8.6 Hz, 2H).

^{13}C NMR (101 MHz, Chloroform-*d*) δ (ppm) = 188.99, 165.75 (d, *J* = 254.4 Hz), 145.21, 134.90, 134.67 (d, *J* = 3.0 Hz), 131.24 (d, *J* = 9.2 Hz), 130.81, 129.14, 128.62, 121.71, 115.90 (d, *J* = 21.8 Hz).

^{19}F NMR (376 MHz, Chloroform-*d*) δ (ppm) = -105.51.

XRF analysis: I (16 ppm), Ru (0.1 ppm), Pd (1.5 ppm).

(E)-3-phenyl-1-(4-(trifluoromethyl)phenyl)prop-2-en-1-one (4m) was obtained by following the general procedure on a 1 mmol scale and isolated by flash column chromatography (EtOAc:pentane = 10:90) as a white solid. Yield: 218 mg, 79%. NMR data of the compound is in good agreement with the literature data.[7]

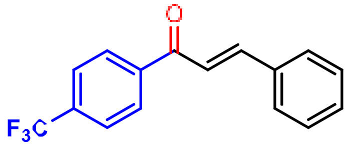

^{1}H NMR (400 MHz, Chloroform-*d*) δ (ppm) = 8.10 (d, *J* = 8.0 Hz, 2H), 7.84 (d, *J* = 15.7 Hz, 1H), 7.77 (d, *J* = 8.1 Hz, 2H), 7.66 (dd, *J* = 6.6, 2.9 Hz, 2H), 7.49 (d, *J* = 15.7 Hz, 1H), 7.47 – 7.44 (m, 3H).

^{13}C NMR (101 MHz, Chloroform-*d*) δ (ppm) = 189.83, 146.28, 141.18, 134.63, 134.33, 134.00, 131.13, 129.20, 128.91, 128.75, 125.87, 125.84, 125.80, 125.76, 121.70.

^{19}F NMR (376 MHz, Chloroform-*d*) δ (ppm) = -63.21.

(E)-1-(4-morpholinophenyl)-3-phenylprop-2-en-1-one (4n) was obtained by following the general procedure on a 1 mmol scale and isolated by flash column chromatography (EtOAc:pentane =20:80) as an off-white solid. Yield: 202 mg, 69%. NMR data of the compound is in good agreement with the literature data.[9]

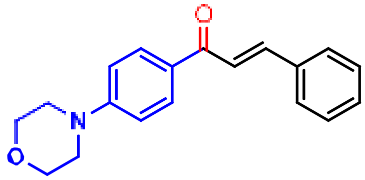

^{1}H NMR (400 MHz, Chloroform-*d*) δ 8.02 (d, *J* = 8.6 Hz, 2H), 7.80 (d, *J* = 15.6 Hz, 1H), 7.65 (dd, *J* = 7.3, 2.3 Hz, 2H), 7.56 (d, *J* = 15.6 Hz, 1H), 7.43 – 7.40 (m, 3H), 6.92 (d, *J* = 8.7 Hz, 2H), 3.87 (t, *J* = 4.8 Hz, 4H), 3.34 (t, *J* = 4.9 Hz, 4H).

^{13}C NMR (101 MHz, Chloroform-*d*) δ (ppm) = 188.32, 154.33, 143.49, 135.42, 130.79, 130.31, 129.04, 128.97, 128.45, 122.09, 113.57, 66.73, 47.65.

methyl 4-cinnamoylbenzoate (4o) was obtained by following the general procedure on a 1 mmol scale and isolated by flash column chromatography (EtOAc:pentane =10:90) as an off-white solid. Yield: 210 mg, 79%. NMR data of the compound is in good agreement with the literature data.[7]

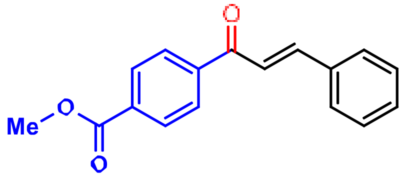

^{1}H NMR (400 MHz, Chloroform-*d*) δ (ppm) = 8.17 (d, *J* = 8.5 Hz, 2H), 8.06 (d, *J* = 8.5 Hz, 2H), 7.83 (d, *J* = 15.7 Hz, 1H), 7.67 – 7.65 (m, 2H), 7.51 (d, *J* = 15.7 Hz, 1H), 7. 45 – 7.42 (m, 3H), 3.97 (s, 3H).

^{13}C NMR (101 MHz, Chloroform-*d*) δ (ppm) = 190.29, 166.47, 145.98, 141.80, 134.75, 133.68, 131.03, 130.01, 129.19, 128.74, 128.53, 121.97, 52.63.

(E)-3-phenyl-1-(thiophen-2-yl)prop-2-en-1-one (4r) was obtained by following the general

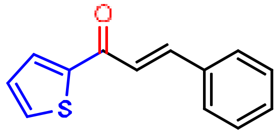

procedure on a 1 mmol scale and isolated by flash column chromatography (EtOAc:pentane = 10:90) as an off-white solid. Yield: 186 mg, 87%. NMR data of the compound is in good agreement with the literature data.[7]

^{1}H NMR (400 MHz, Chloroform-*d*) δ (ppm) = 7.88 (s, 1H), 7.86 (d, *J* = 12.1 Hz, 1H), 7.69 (d, *J* = 4.9 Hz, 1H), 7.65 (dd, *J* = 6.5, 2.9 Hz, 2H), 7.45 – 7.41 (m, 4H), 7.19 (t, *J* = 4.4 Hz, 1H).

^{13}C NMR (101 MHz, Chloroform-*d*) δ (ppm) = 182.19, 145.67, 144.24, 134.85, 134.05, 131.95, 130.75, 129.12, 128.64, 128.40, 121.76.

(E)-1-([1,1'-biphenyl]-4-yl)-3-phenylprop-2-en-1-one (4u) was obtained by following the

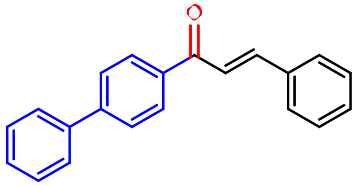

general procedure on a 1 mmol scale and isolated by flash column chromatography (EtOAc:pentane = 10:90) as an pale yellow solid. Yield: 236 mg, 83%. NMR data of the compound is in good agreement with the literature data.[10]

^{1}H NMR (400 MHz, Chloroform-*d*) δ (ppm) = 8.13 – 8.11 (m, 2H), 7.86 (d, *J* = 15.7 Hz, 1H), 7.75 – 7.73 (m, 2H), 7.69 – 7.65 (m, 4H), 7.59 (d, *J* = 15.7 Hz, 1H), 7.51 – 7.47 (m, 2H), 7.46 – 7.39 (m, 4H).

^{13}C NMR (101 MHz, Chloroform-*d*) δ (ppm) = 190.13, 145.70, 144.93, 140.10, 137.05, 135.08, 130.71, 129.28, 129.13, 129.12, 128.63, 128.37, 127.45, 122.16.

(*E*)-3-(4-(tert-butyl)phenyl)-1-phenylprop-2-en-1-one (**4y**) was obtained by following the

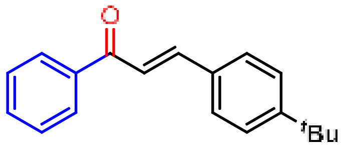

general procedure on a 1 mmol scale and isolated by flash column chromatography (EtOAc:pentane = 10:90) as a off-white solid. Yield: 219 mg, 83%. NMR data of the compound is in good agreement with the literature data.[7]

^{1}H NMR (400 MHz, Chloroform-*d*) δ (ppm) = 8.08 – 8.02 (m, 2H), 7.82 (d, *J* = 15.7 Hz, 1H), 7.61 – 7.44 (m, 8H), 1.35 (s, 9H).

^{13}C NMR (101 MHz, Chloroform-*d*) δ (ppm) = 190.48, 154.01, 144.65, 138.15, 132.47, 131.92, 128.39, 128.28, 128.14, 125.74, 121.08, 34.74, 30.95.

(*E*)-3-(Naphthalen-2-yl)-1-phenylprop-2-en-1-one (**4ag**) was obtained by following the general procedure on a 1 mmol scale and isolated by flash column chromatography (EtOAc:pentane = 10:90) as a yellow solid. Yield: 207 mg, 80%. NMR data of the compound is in good agreement with the literature data.[11]

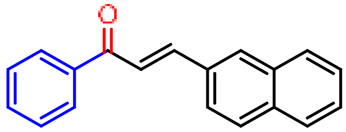

^{1}H NMR (400 MHz, Chloroform-*d*) δ (ppm) = 8.08 – 8.04 (m, 3H), 7.99 (d, *J* = 15.8 Hz, 1H), 7.90 – 7.80 (m, 4H), 7.66 (d, *J* = 15.7 Hz, 1H), 7.60 (d, *J* = 7.3 Hz, 1H), 7.55 – 7.51 (m, 4H).

^{13}C NMR (101 MHz, Chloroform-*d*) δ (ppm) = 190.68, 145.09, 138.42, 134.52, 133.49, 132.94, 132.51, 130.82, 128.88, 128.79, 128.67, 127.94, 127.53, 126.92, 123.79, 122.32.

XRF analysis: I (16 ppm), Ru (0.2 ppm), Pd (1.7 ppm).

(*E*)-3-([1,1'-biphenyl]-4-yl)-1-phenylprop-2-en-1-one (4ah) was obtained by following the general procedure on a 1 mmol scale and isolated by flash column chromatography (EtOAc:pentane = 10:90) as a yellow solid. Yield: 202 mg, 71%. NMR data of the compound is in good agreement with the literature data.[11]

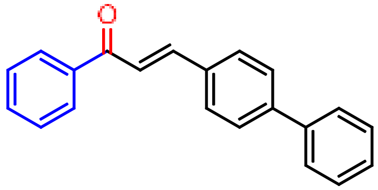

^{1}H NMR (400 MHz, Chloroform-*d*) δ (ppm) = 8.05 (d, *J* = 7.4 Hz, 2H), 7.87 (d, *J* = 15.7 Hz, 1H), 7.73 (d, *J* = 8.0 Hz, 2H), 7.68 – 7.58 (m, 6H), 7.51 (dt, *J* = 28.0, 7.6 Hz, 4H), 7.39 (t, *J* = 7.3 Hz, 1H).

^{13}C NMR (101 MHz, Chloroform-*d*) δ (ppm) = 190.66, 144.56, 143.46, 140.26, 138.40, 133.97, 132.94, 129.13, 129.07, 128.79, 128.65, 128.05, 127.75, 127.20, 122.03.

XRF analysis: I (13 ppm), Ru (0.2 ppm), Pd (0.2 ppm).

(*E*)-3-(2-chlorophenyl)-1-(4-methoxyphenyl)prop-2-en-1-one (**4ai**) was obtained by following

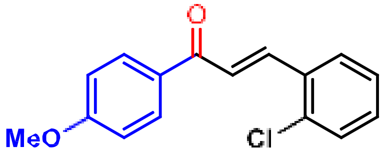

the general procedure on a 1 mmol scale and isolated by flash column chromatography (EtOAc:pentane = 30:70) as a white solid. Yield: 218 mg, 80%. NMR data of the compound is in good agreement with the literature data.[12]

^{1}H NMR (400 MHz, Chloroform-*d*) δ (ppm) = 8.16 (d, *J* = 15.7 Hz, 1H), 8.05 – 8.02 (m, 2H), 7.75 – 7.73 (m, 1H), 7.49 (d, *J* = 15.7 Hz, 1H), 7.45 – 7.42 (m, 1H), 7.34 – 7.30 (m, 2H), 7.00 – 6.97 (m, 2H), 3.91 (s, 3H).

^{13}C NMR (101 MHz, Chloroform-*d*) δ (ppm) = 188.72, 163.65, 139.88, 135.49, 133.56, 131.09, 131.07, 130.92, 130.38, 127.87, 127.16, 124.80, 114.00, 55.62.

(E)-3-(4-(dimethylamino)phenyl)-1-phenylprop-2-en-1-one (4ak) was obtained by following

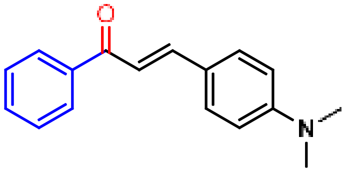

the general procedure on a 1 mmol scale and isolated by flash column chromatography (EtOAc:pentane = 1:10) as a reddish solid. Yield: 176 mg, 70%. NMR data of the compound is in good agreement with the literature data.[13]

^{1}H NMR (400 MHz, Chloroform-*d*) δ (ppm) = 8.01 (d, *J* = 7.5 Hz, 2H), 7.80 (d, *J* = 15.4 Hz, 1H), 7.61 – 7.42 (m, 5H), 7.34 (d, *J* = 15.4 Hz, 1H), 6.69 (d, *J* = 8.4 Hz, 2H), 3.03 (s, 6H).

^{13}C NMR (101 MHz, Chloroform-*d*) δ (ppm) = 190.74, 152.10, 145.95, 139.15, 132.23, 130.51, 128.53, 128.39, 122.68, 116.94, 111.90, 40.20.

12. ^1H and ^{13}C NMR Spectra of Isolated Products

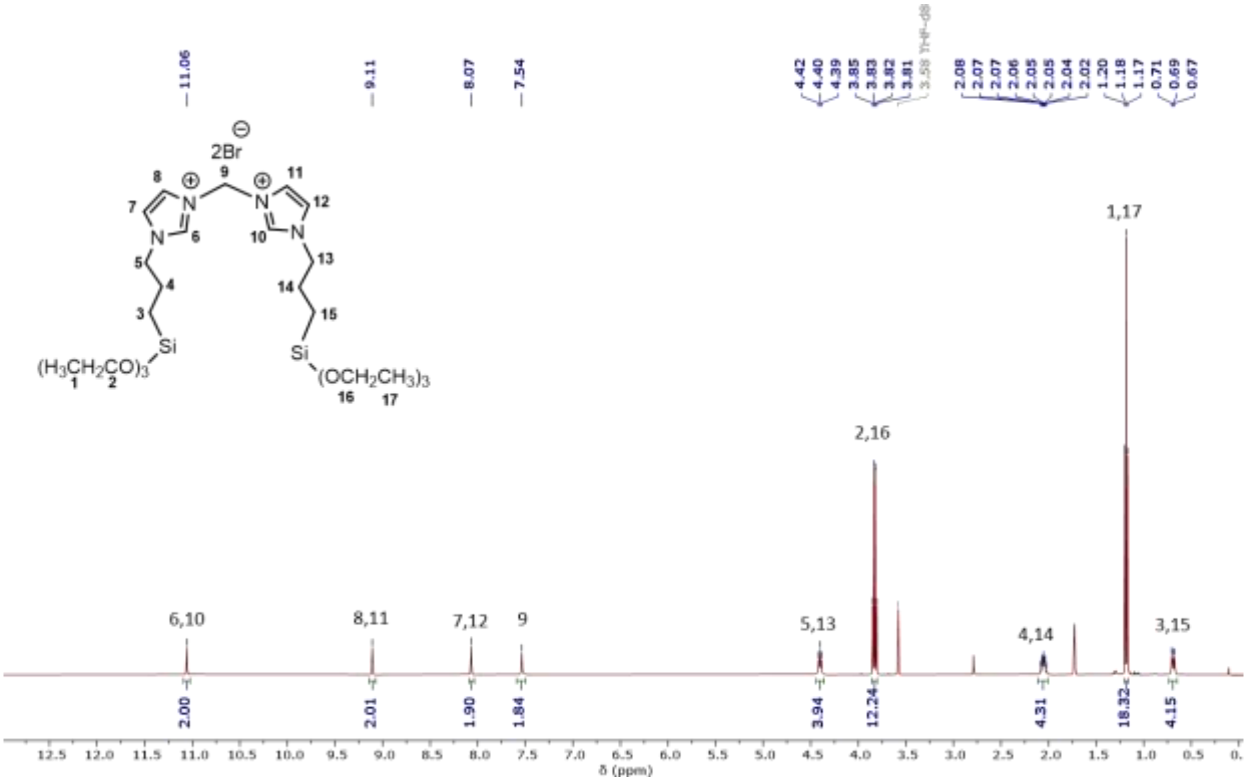

Figure S20. ^1H NMR (500 MHz, THF-d8) spectrum of ligand **9.**

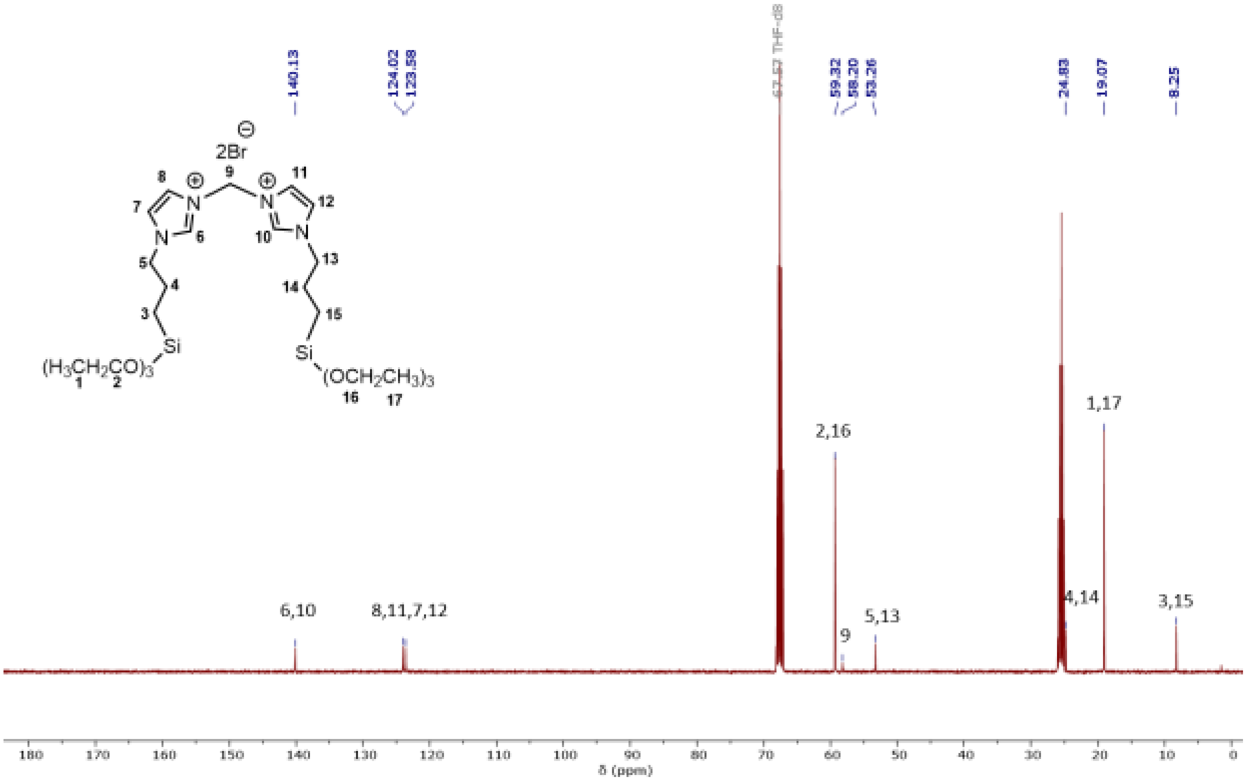

Figure S21. ^{13}C NMR (126 MHz, THF-d_8) spectrum of ligand **9.**

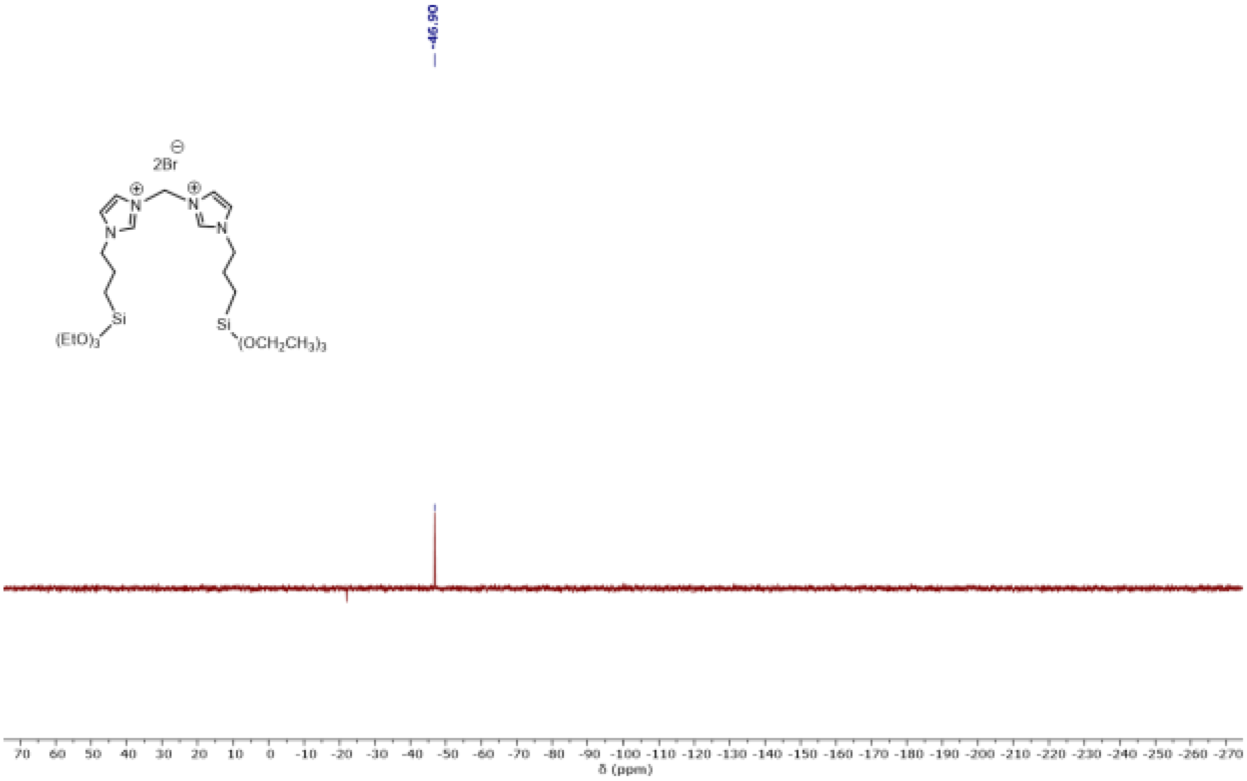

Figure S22. ^{29}Si NMR (79 MHz, THF-d_8) spectrum of ligand **9.**

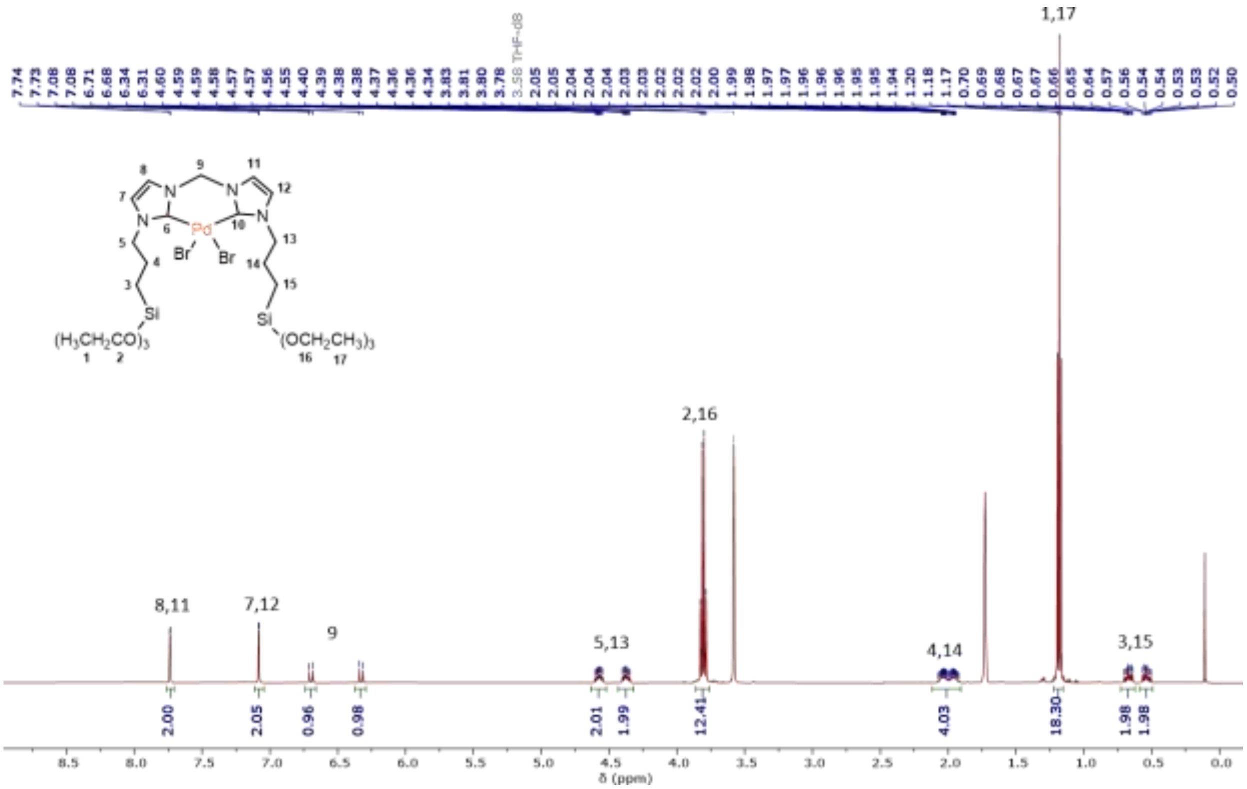

Figure S23. ^{1}H NMR (500 MHz, THF-d8) spectrum of complex **10.**

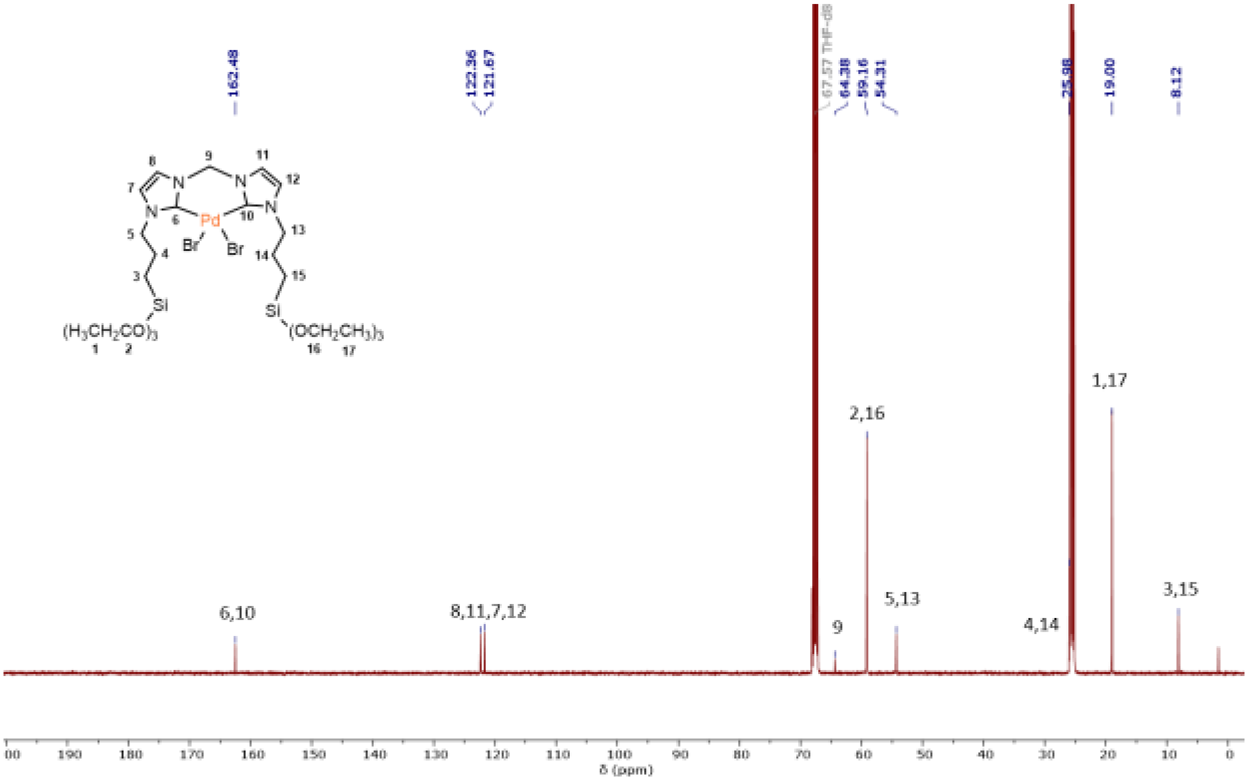

Figure S24. ^{13}C NMR (126 MHz, THF-d_8) spectrum of complex **10.**

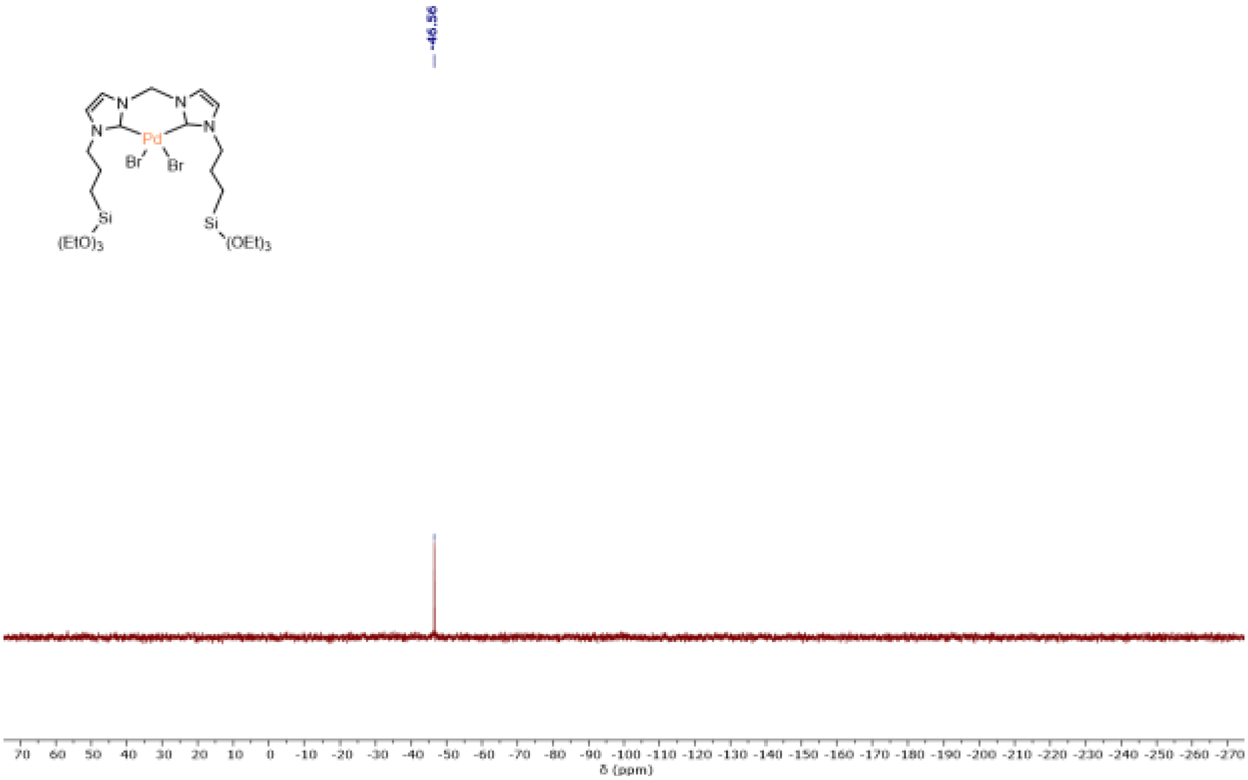

Figure S25. ^{29}Si NMR (79 MHz, THF-d_8) spectrum of complex **10.**

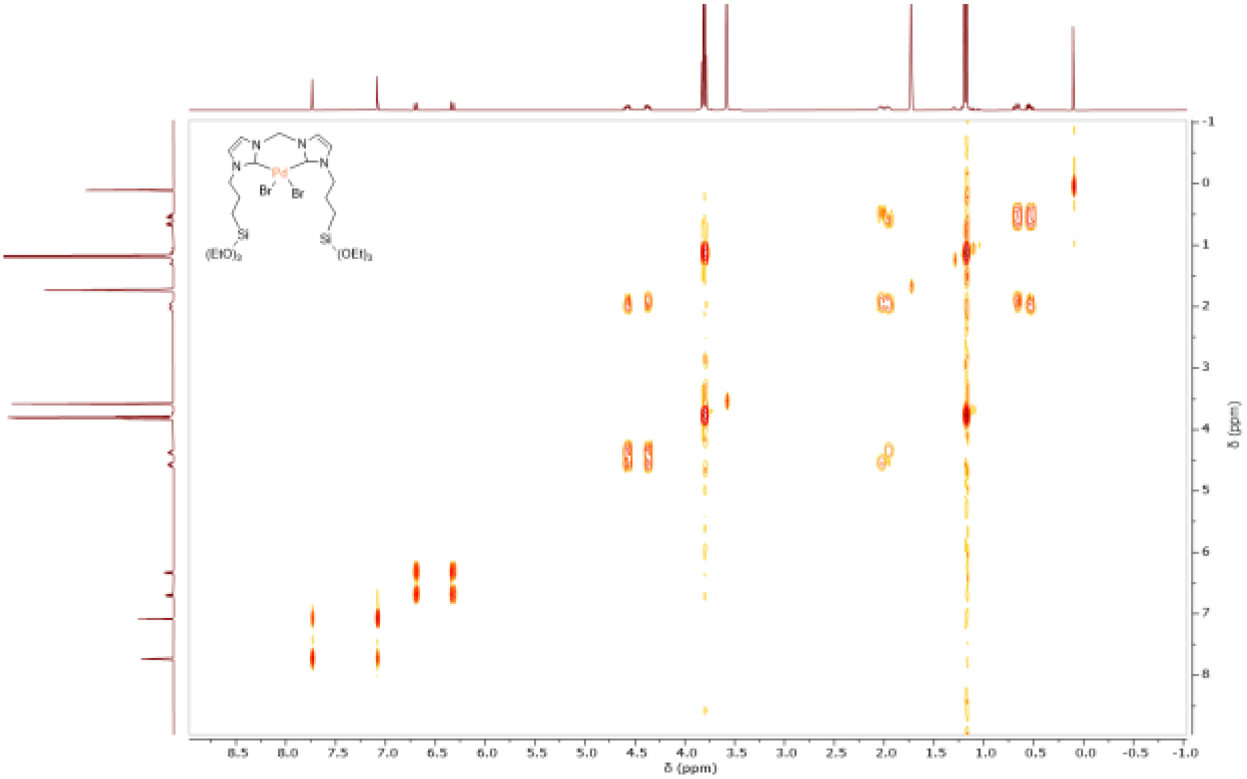

Figure S26. ^{1}H,^{1}H-COSY NMR (500 MHz, THF-d_8) spectrum of complex **10.**

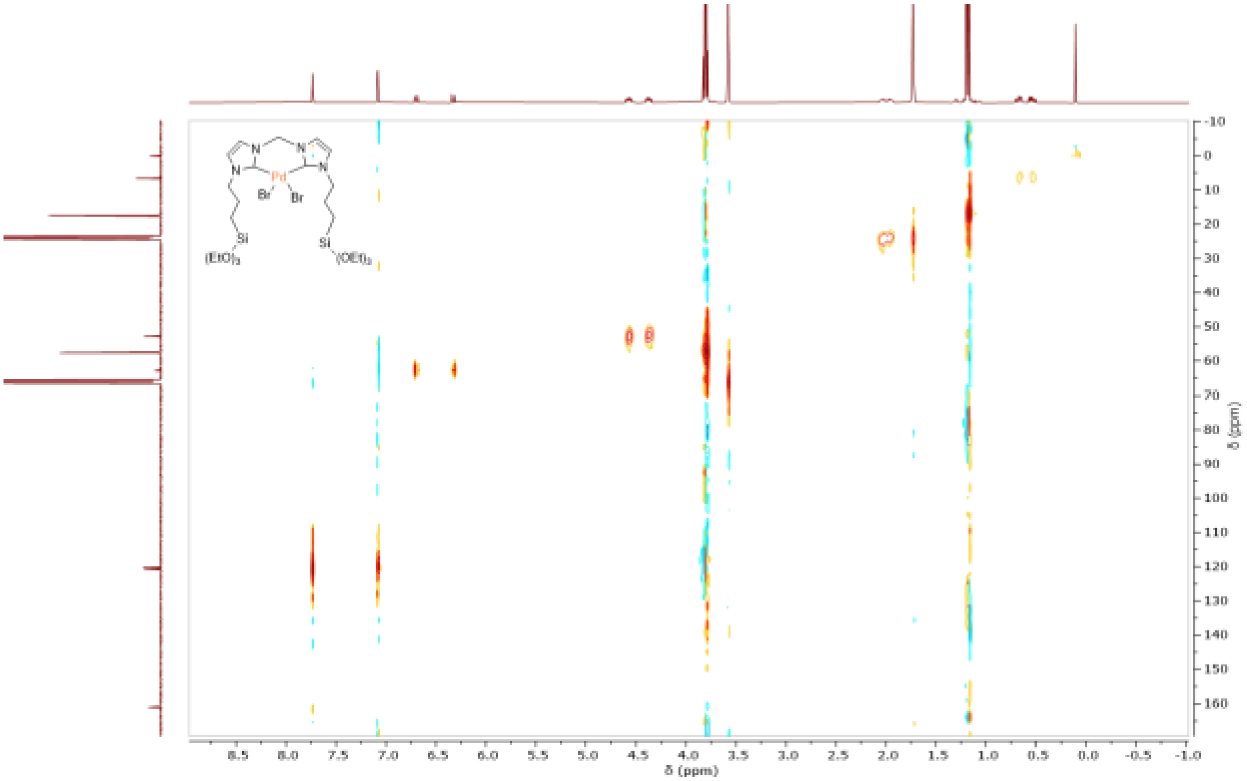

Figure S27. ^{1}H,^{13}C-HSQC NMR (500, 126 MHz, THF-d_8) spectrum of complex **10.**

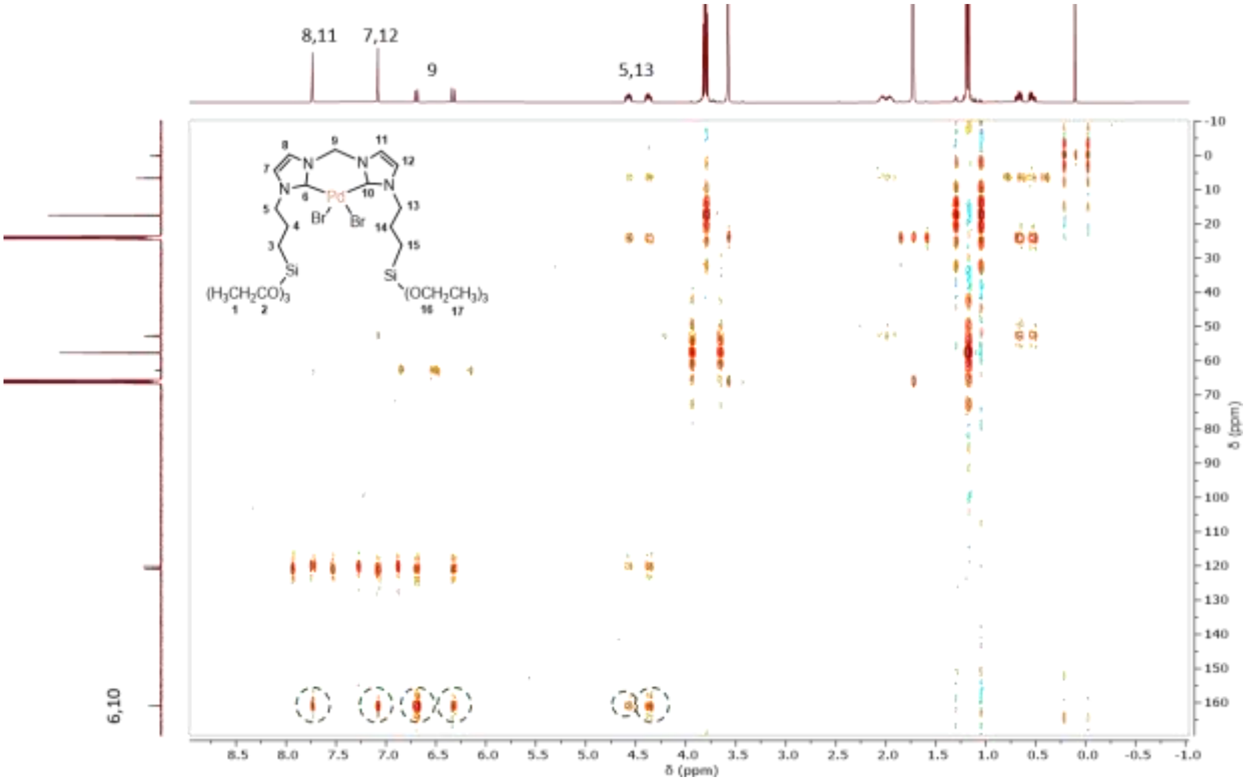

Figure S28. ^{1}H,^{13}C-HMBC NMR (500, 126 MHz, THF-d_8) spectrum of complex **10.**

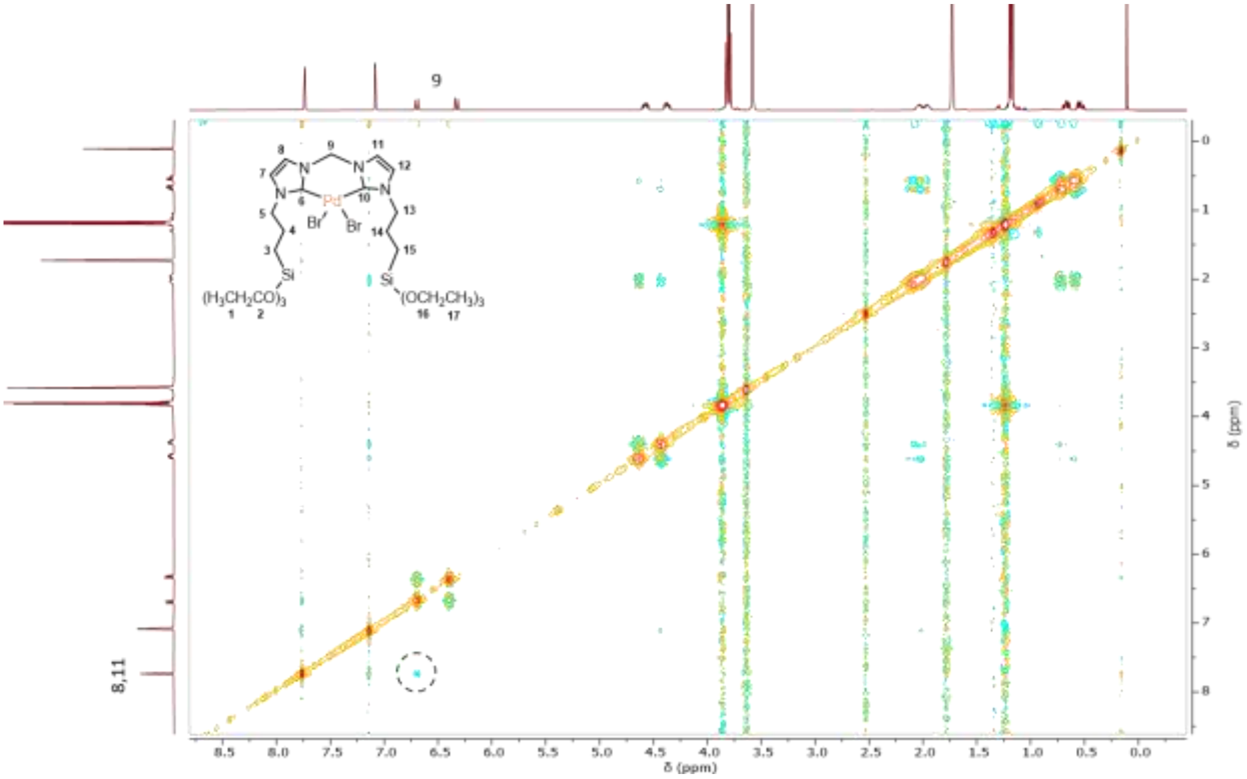

Figure S29. $^{1}H,^{1}H$-NOESY NMR (500 MHz, THF-d_8) spectrum of complex **10.**

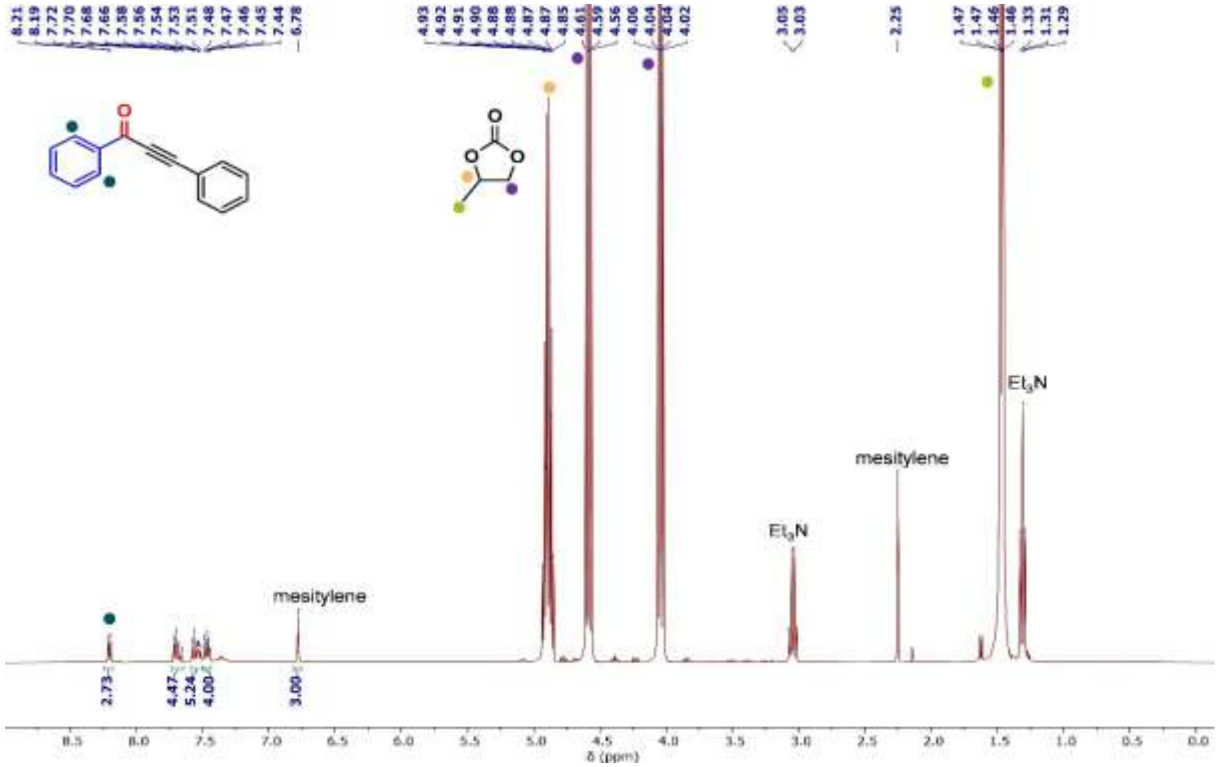

Figure S30. ^{1}H NMR (400 MHz, $CDCl_3$) spectrum of crude reaction (0.3 mmol scale) to yield intermediate **3** with mesitylene as internal standard (0.2 mmol)**.**

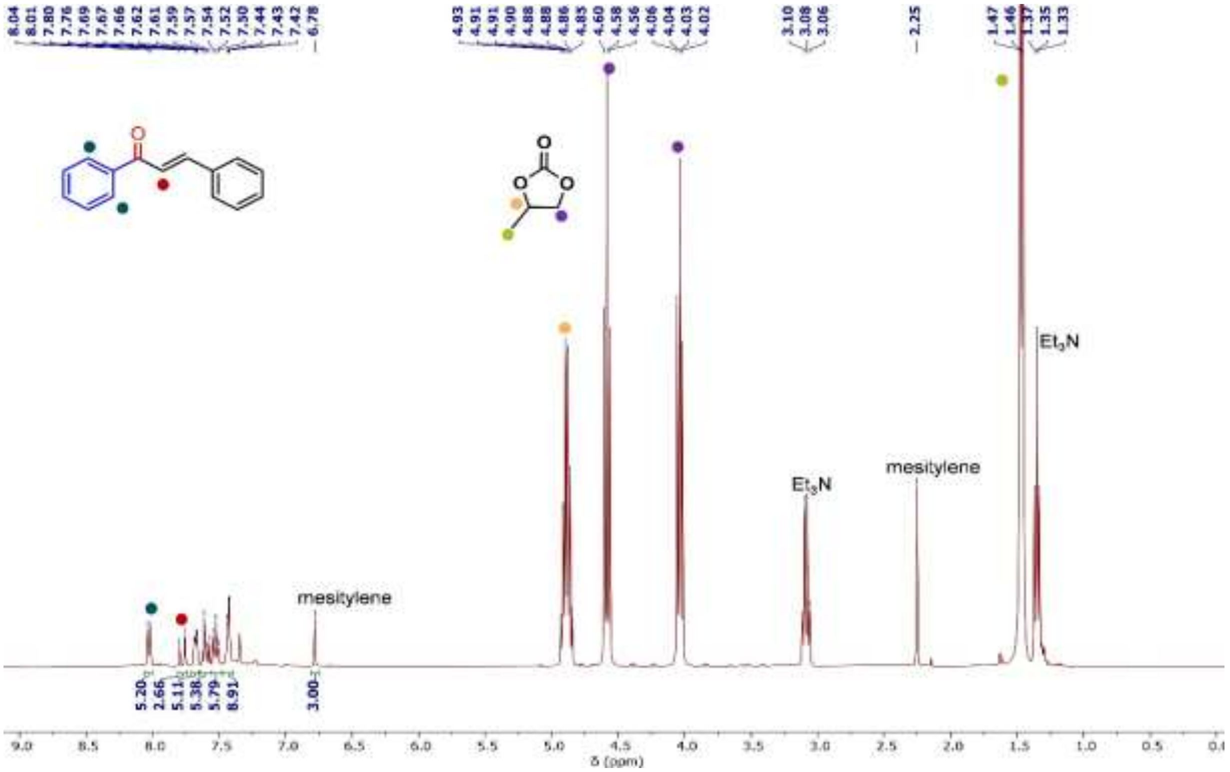

Figure S31. ^{1}H NMR (400 MHz, $CDCl_3$) spectrum of crude reaction (1 mmol scale) to yield product **4** with mesitylene as internal standard (0.35 mmol)**.**

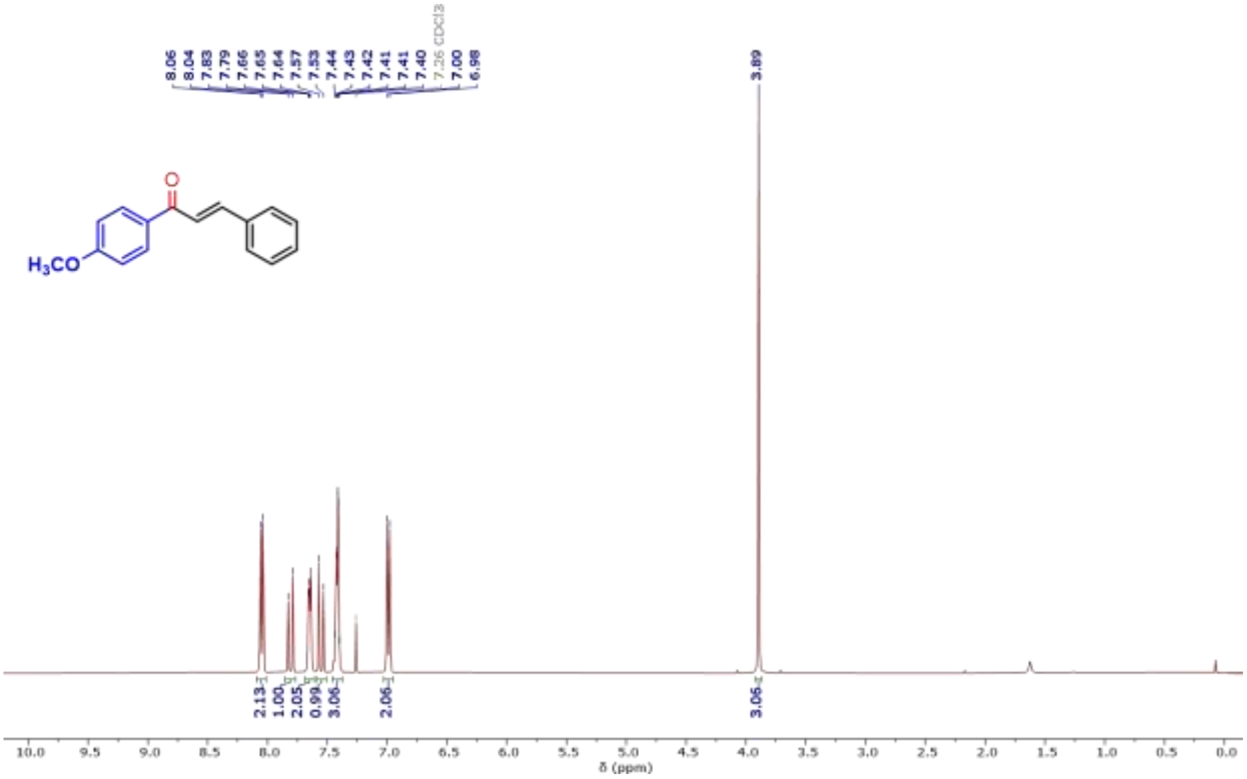

Figure S32. ^{1}H NMR (400 MHz, $CDCl_3$) spectrum of product **4b.**

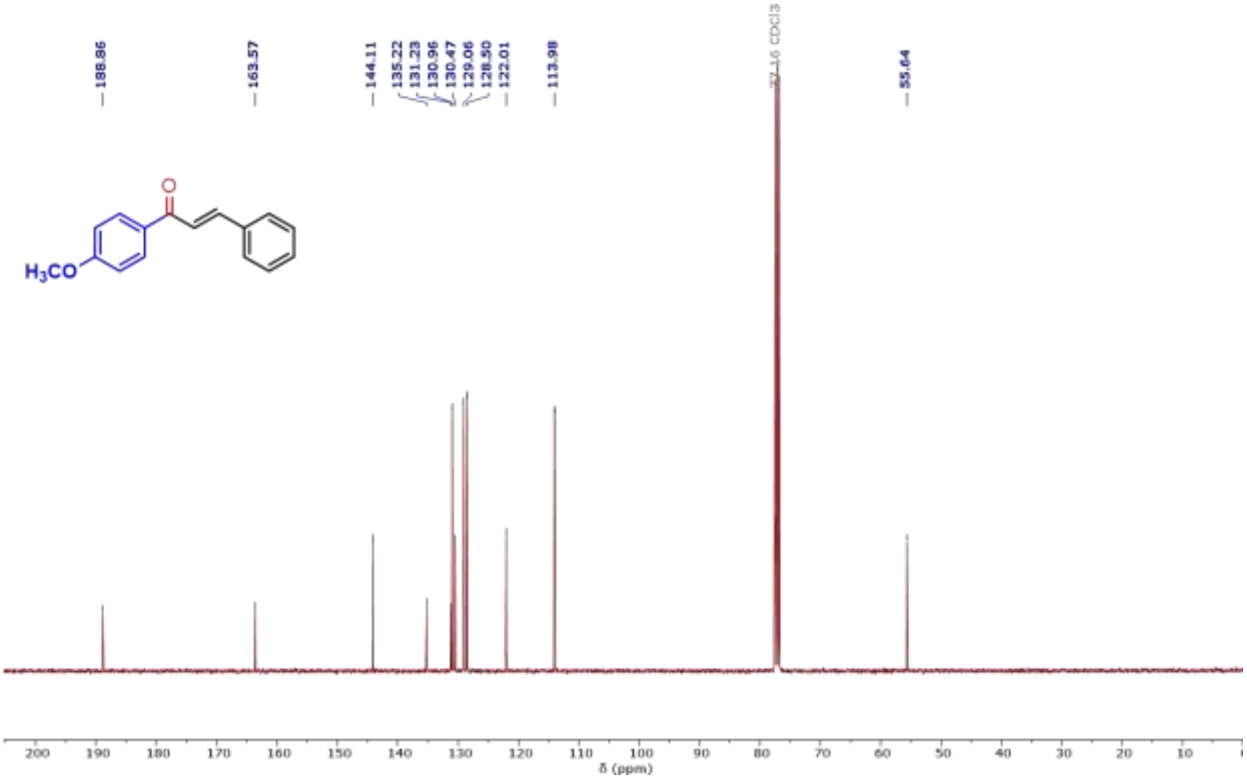

Figure S33. ^{13}C NMR (101 MHz, $CDCl_3$) spectrum of product **4b.**

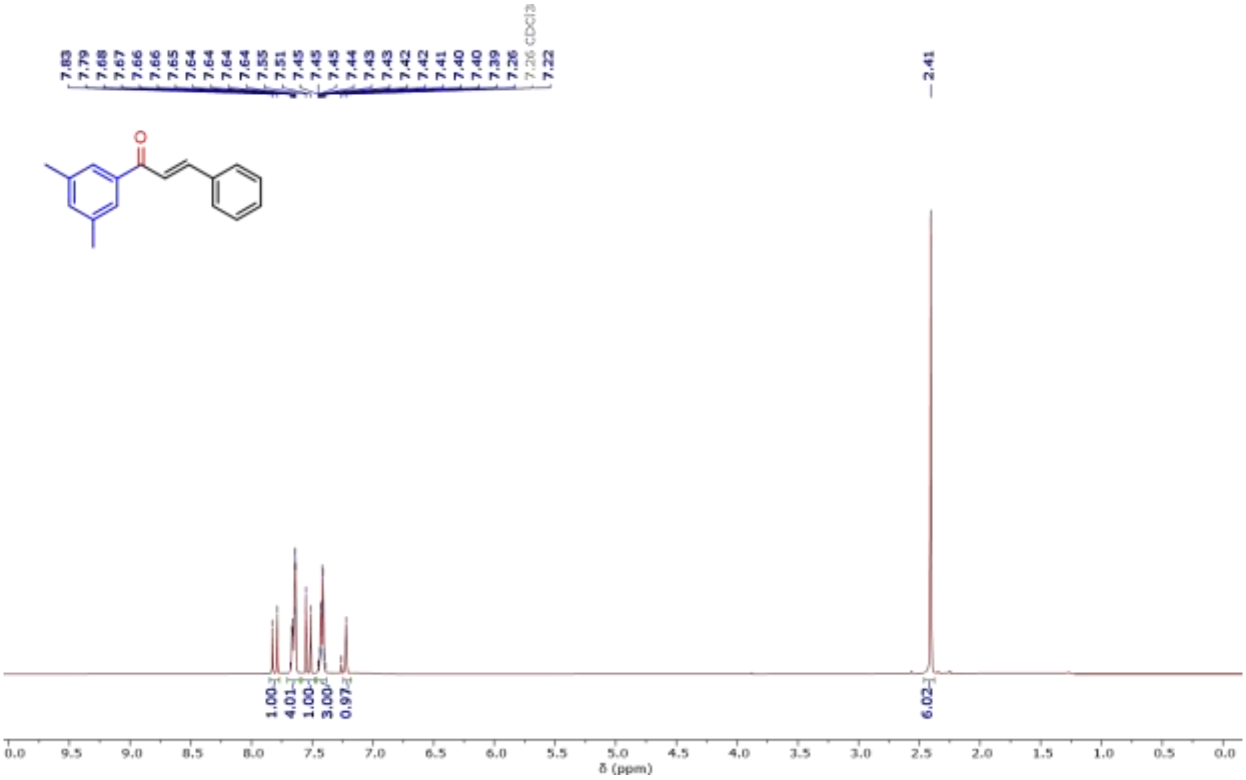

Figure S34. ^{1}H NMR (400 MHz, $CDCl_3$) spectrum of product **4f.**

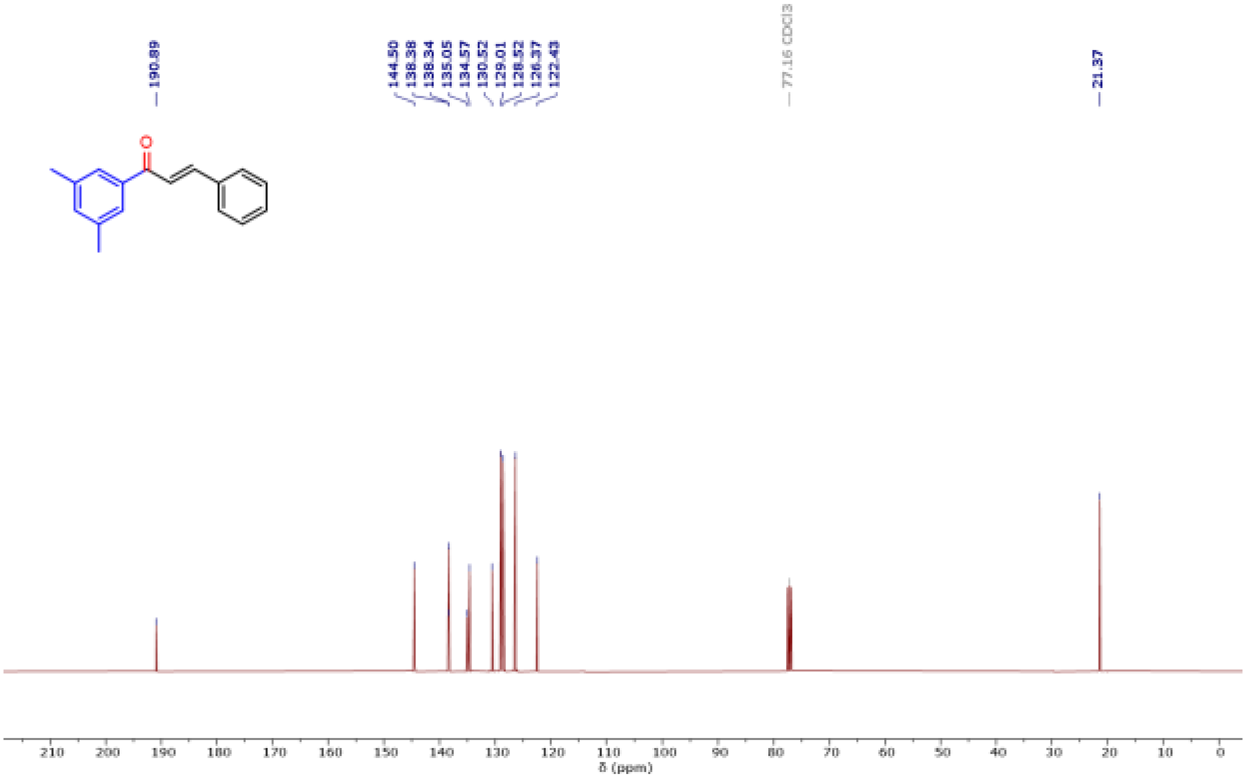

Figure S35. ^{13}C NMR (101 MHz, $CDCl_3$) spectrum of product **4f.**

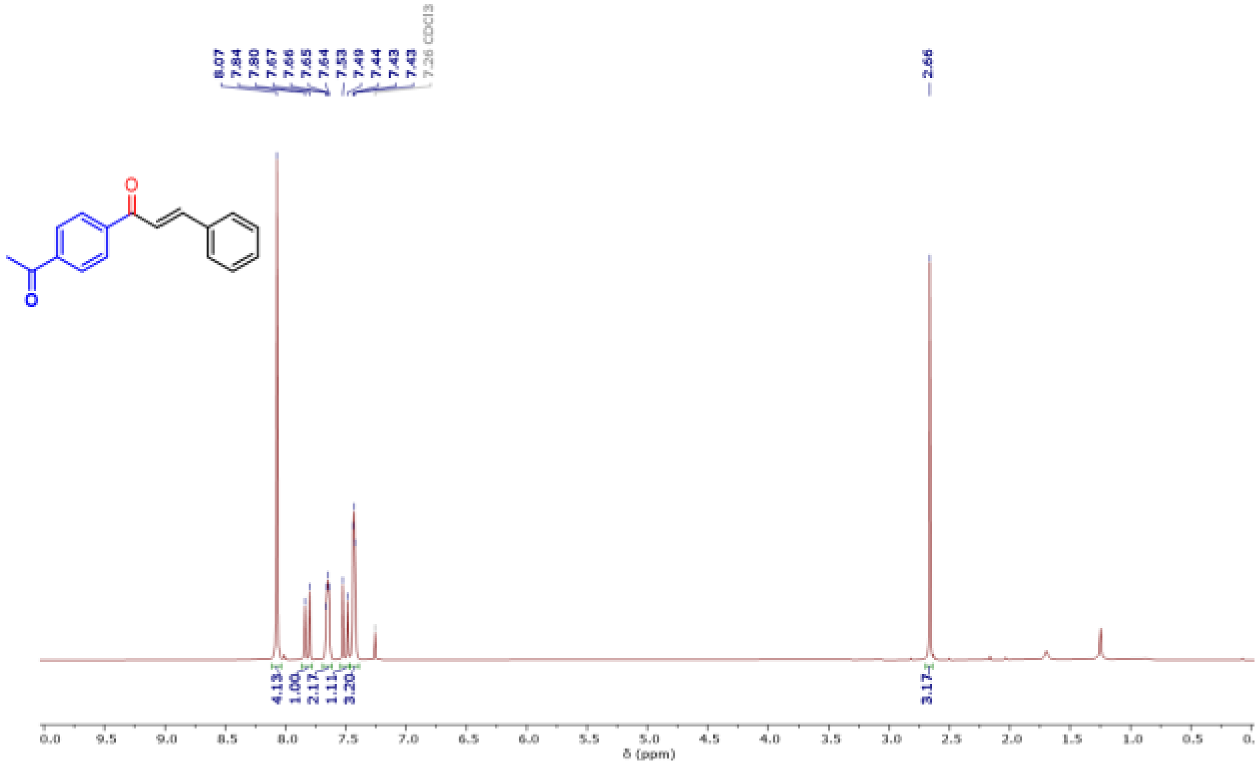

Figure S36. ^{1}H NMR (400 MHz, $CDCl_3$) spectrum of product **4j.**

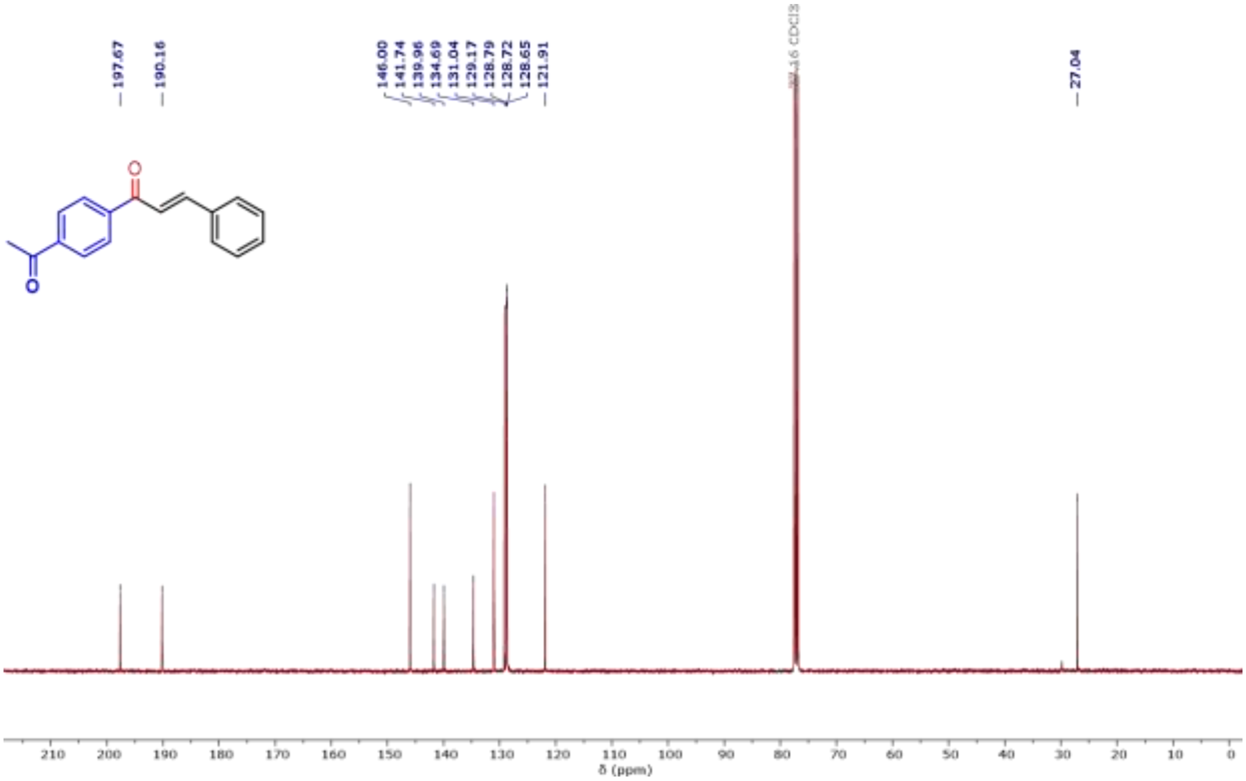

Figure S37. ^{13}C NMR (101 MHz, $CDCl_3$) spectrum of product **4j.**

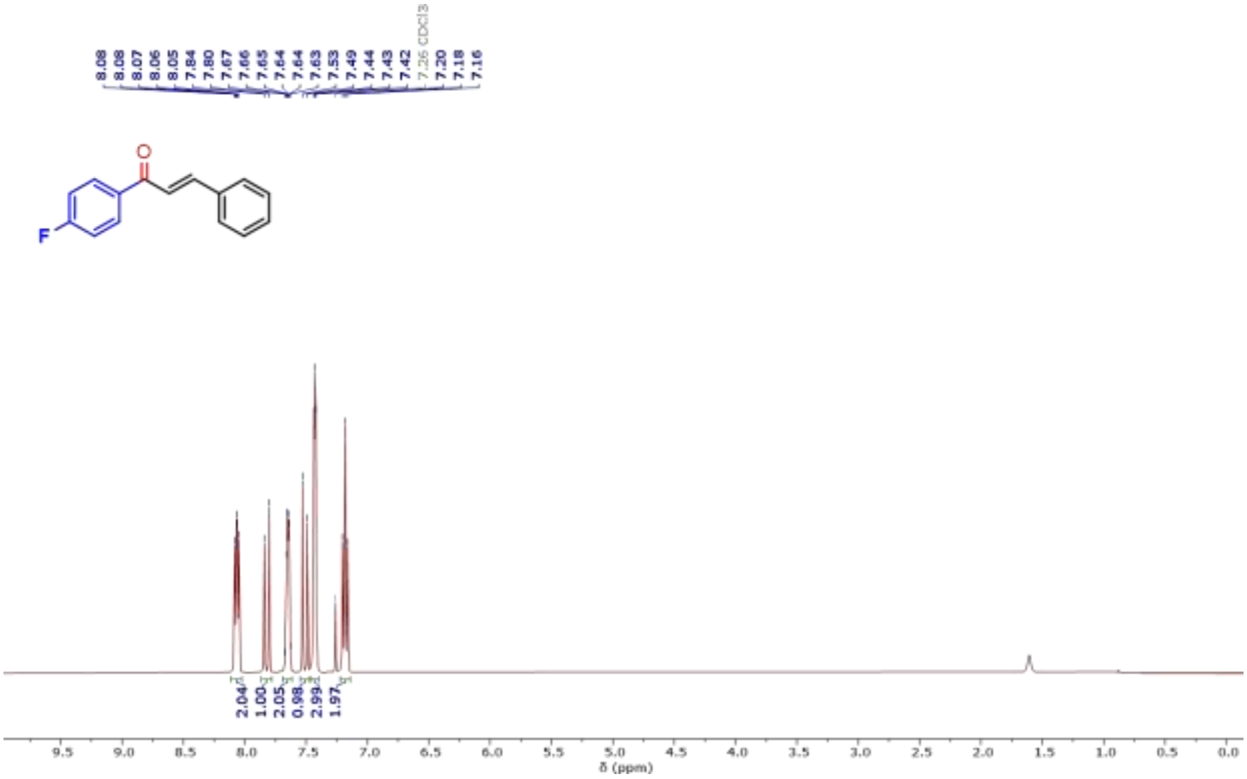

Figure S38. ^{1}H NMR (400 MHz, $CDCl_3$) spectrum of product **4k.**

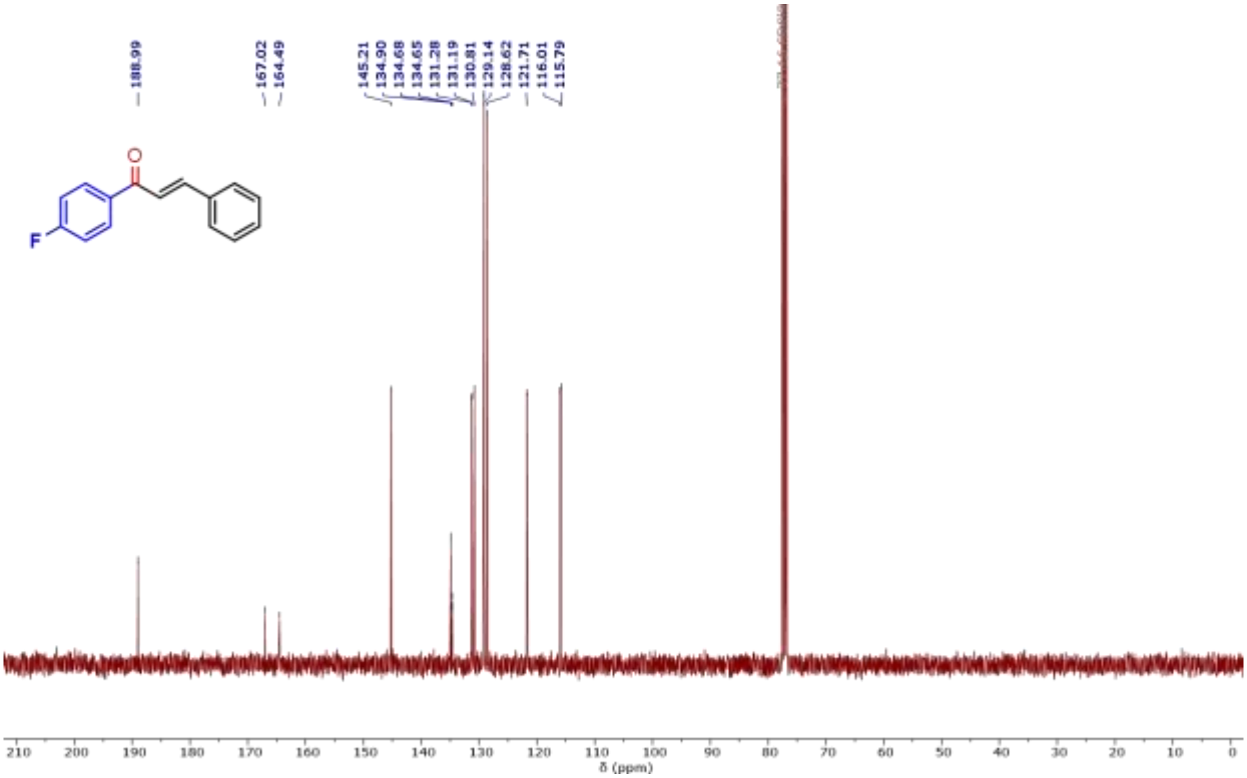

Figure S39. ^{13}C NMR (101 MHz, $CDCl_3$) spectrum of product **4k.**

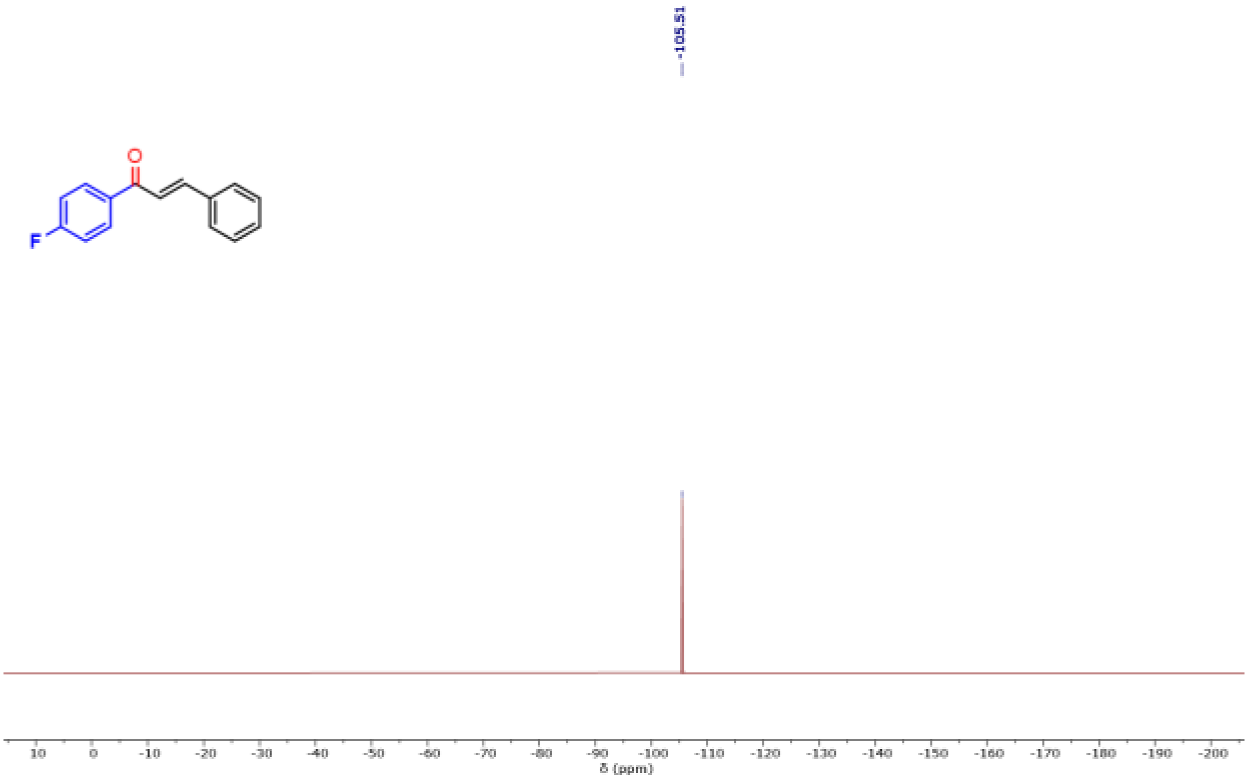

Figure S40. ^{19}F NMR (376 MHz, $CDCl_3$) spectrum of product **4k.**

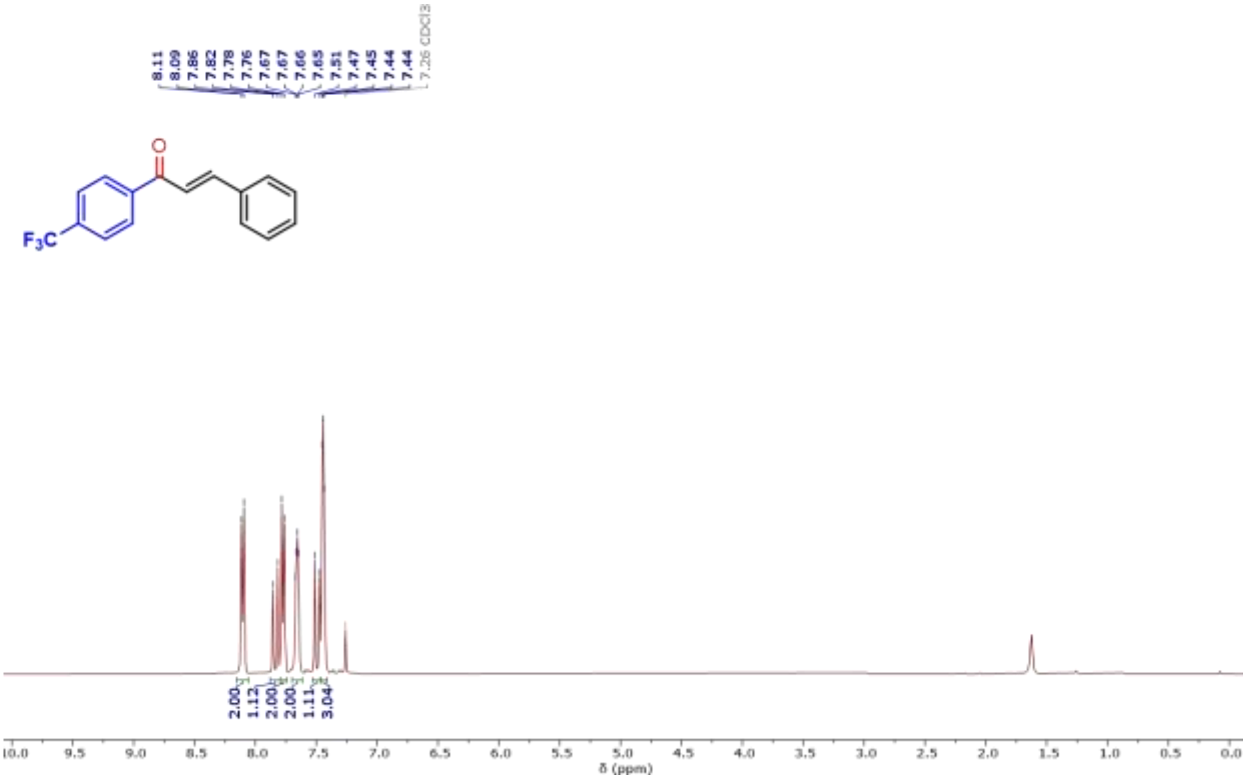

Figure S41. ^{1}H NMR (400 MHz, $CDCl_3$) spectrum of product **4m.**

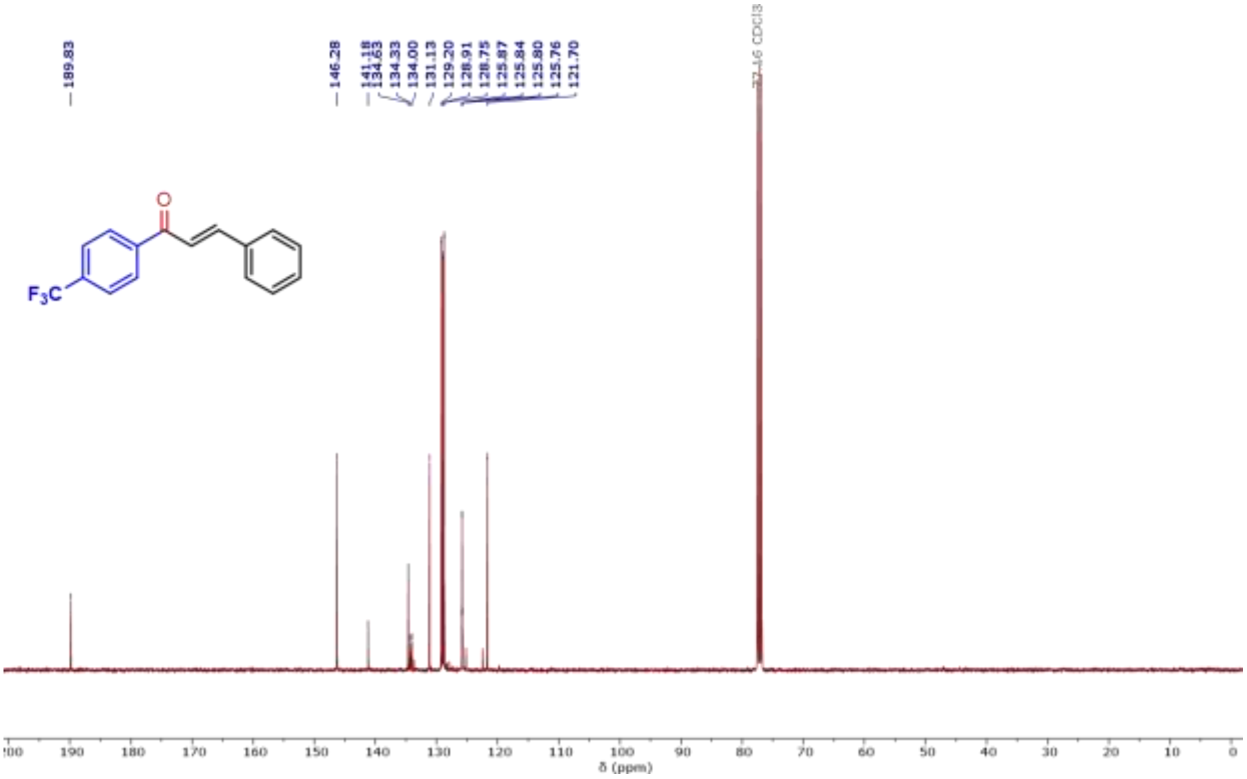

Figure S42. ^{13}C NMR (101 MHz, $CDCl_3$) spectrum of product **4m.**

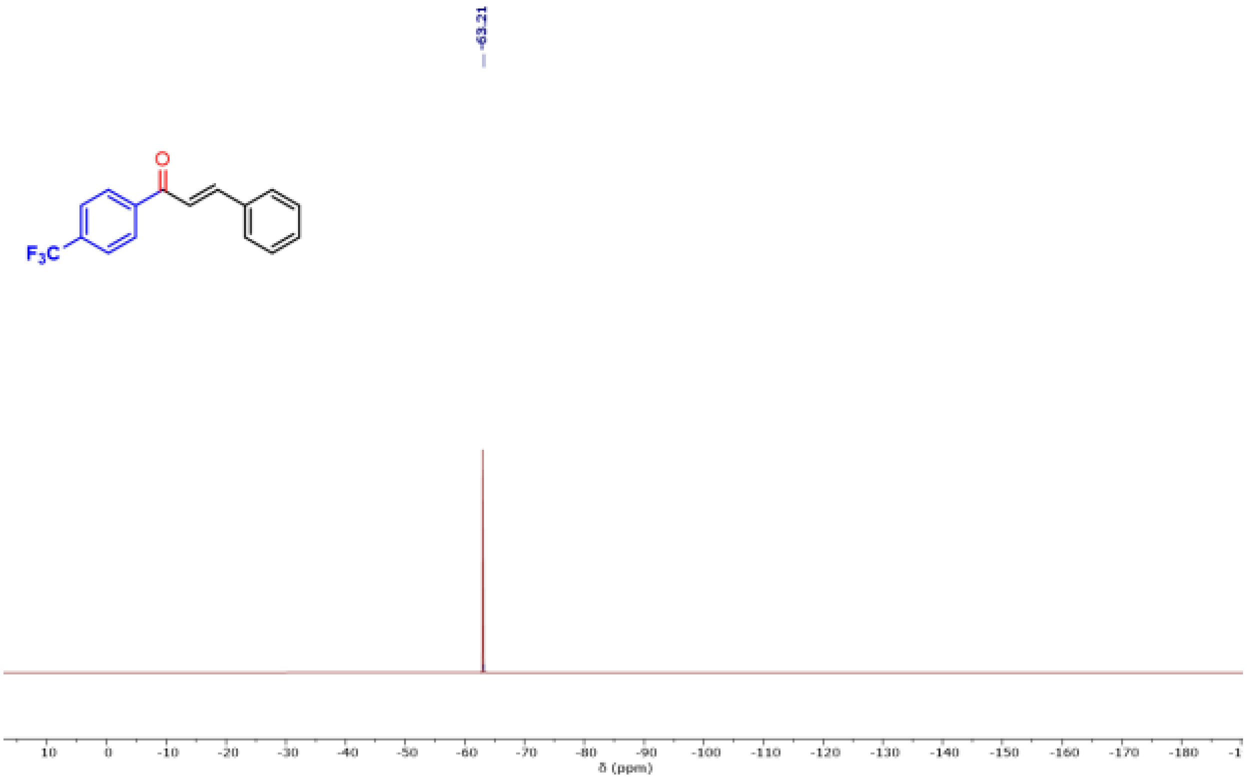

Figure S43. ^{19}F NMR (376 MHz, $CDCl_3$) spectrum of product **4m.**

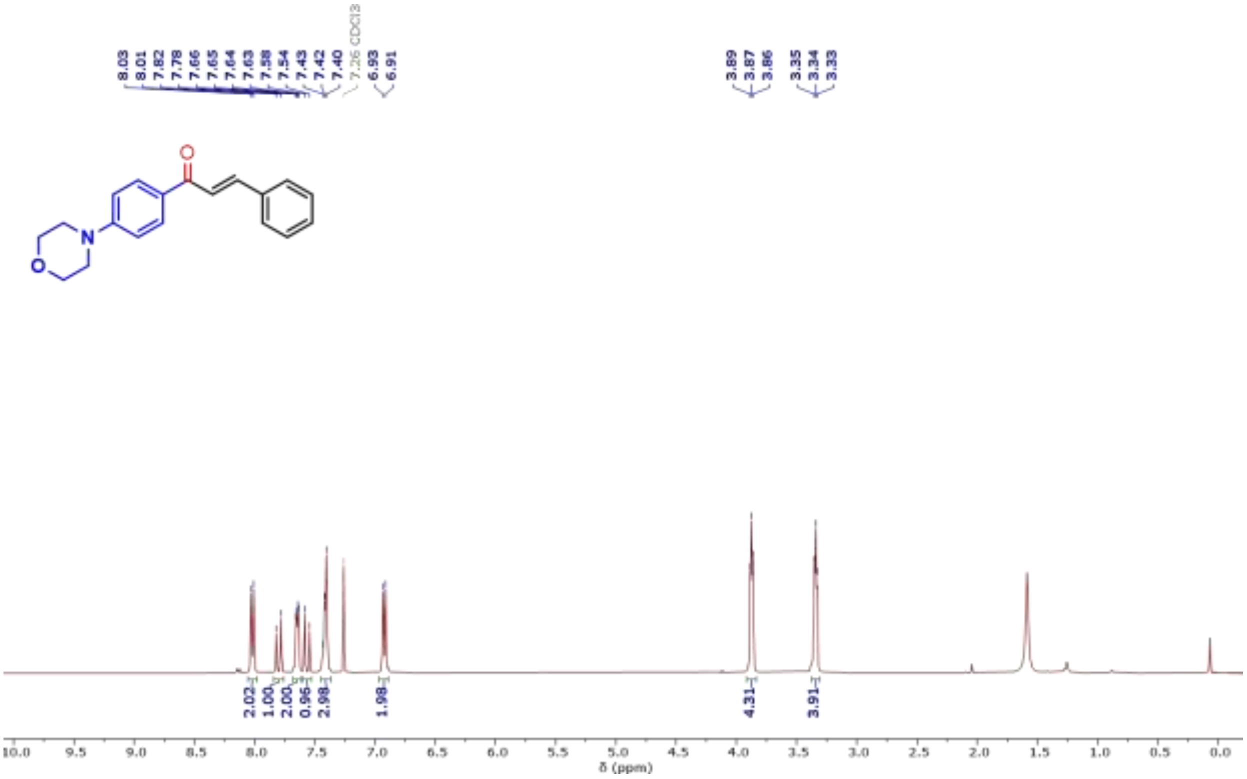

Figure S44. ^{1}H NMR (400 MHz, $CDCl_3$) spectrum of product **4n.**

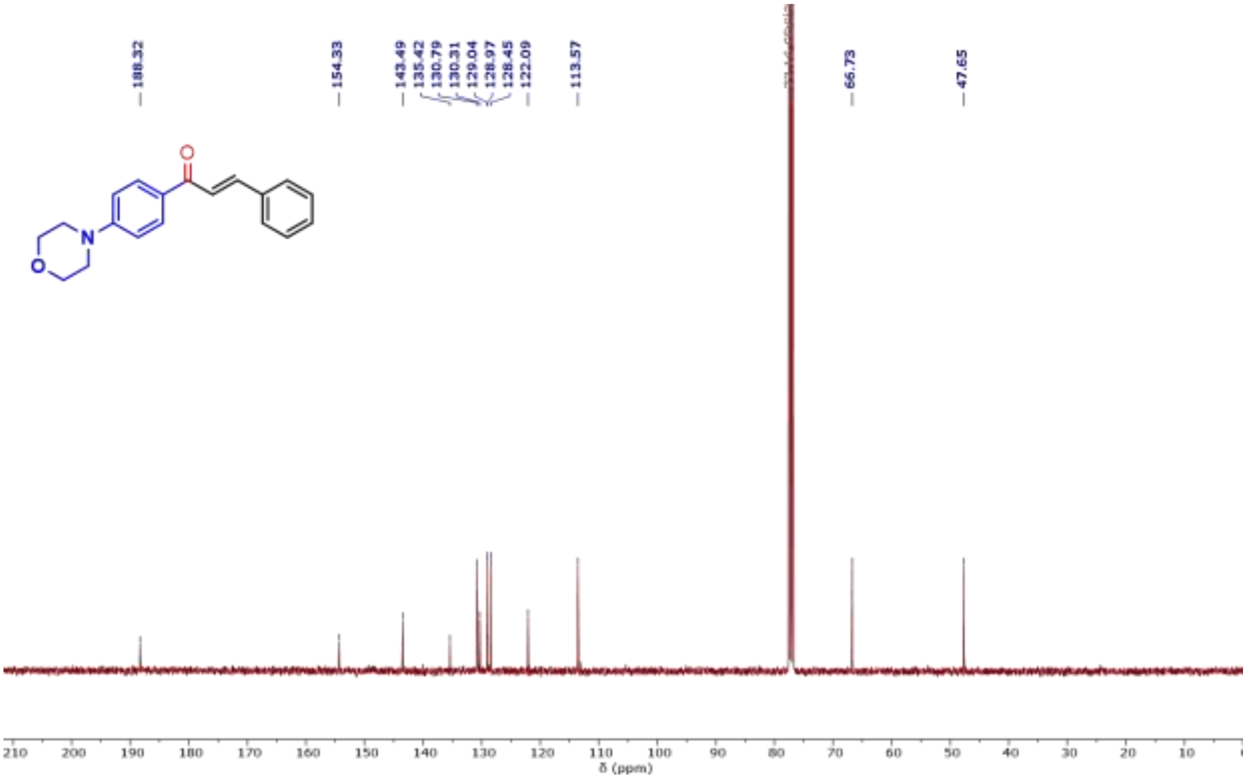

Figure S45. ^{13}C NMR (101 MHz, $CDCl_3$) spectrum of product **4n.**

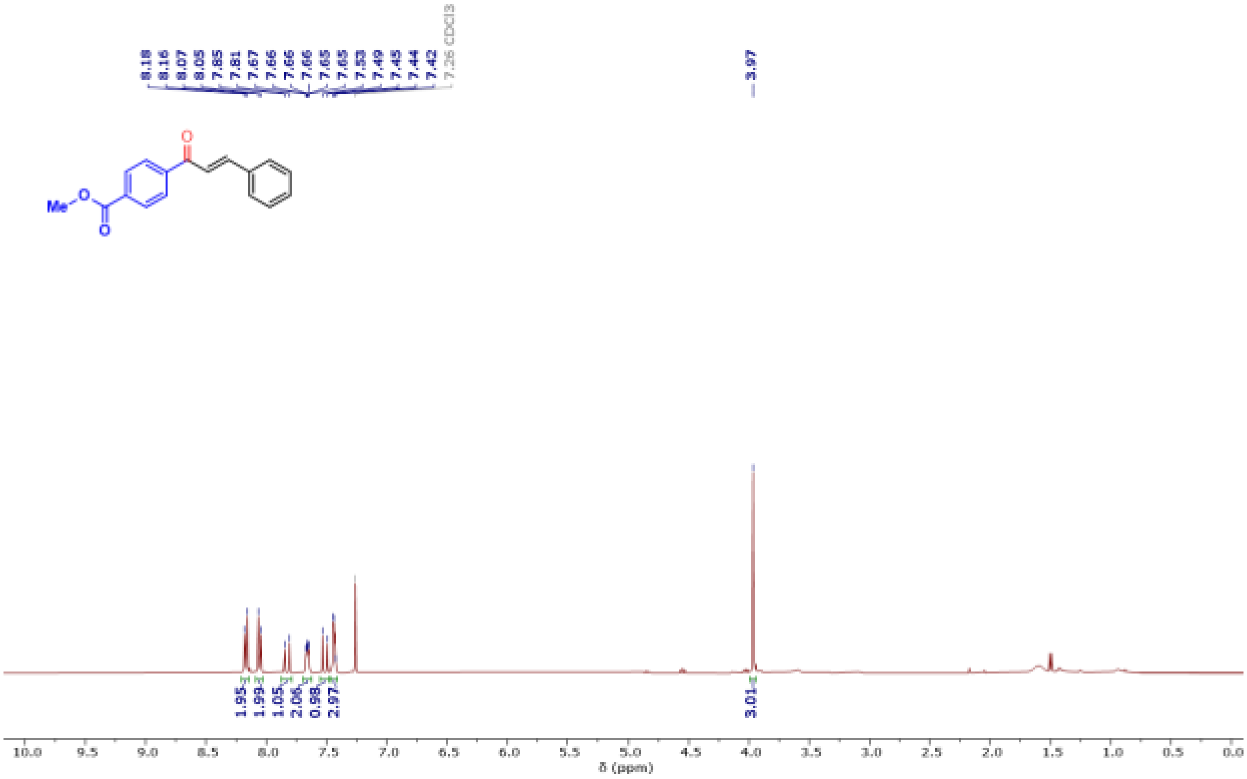

Figure S46. ^{1}H NMR (400 MHz, $CDCl_3$) spectrum of product **4o.**

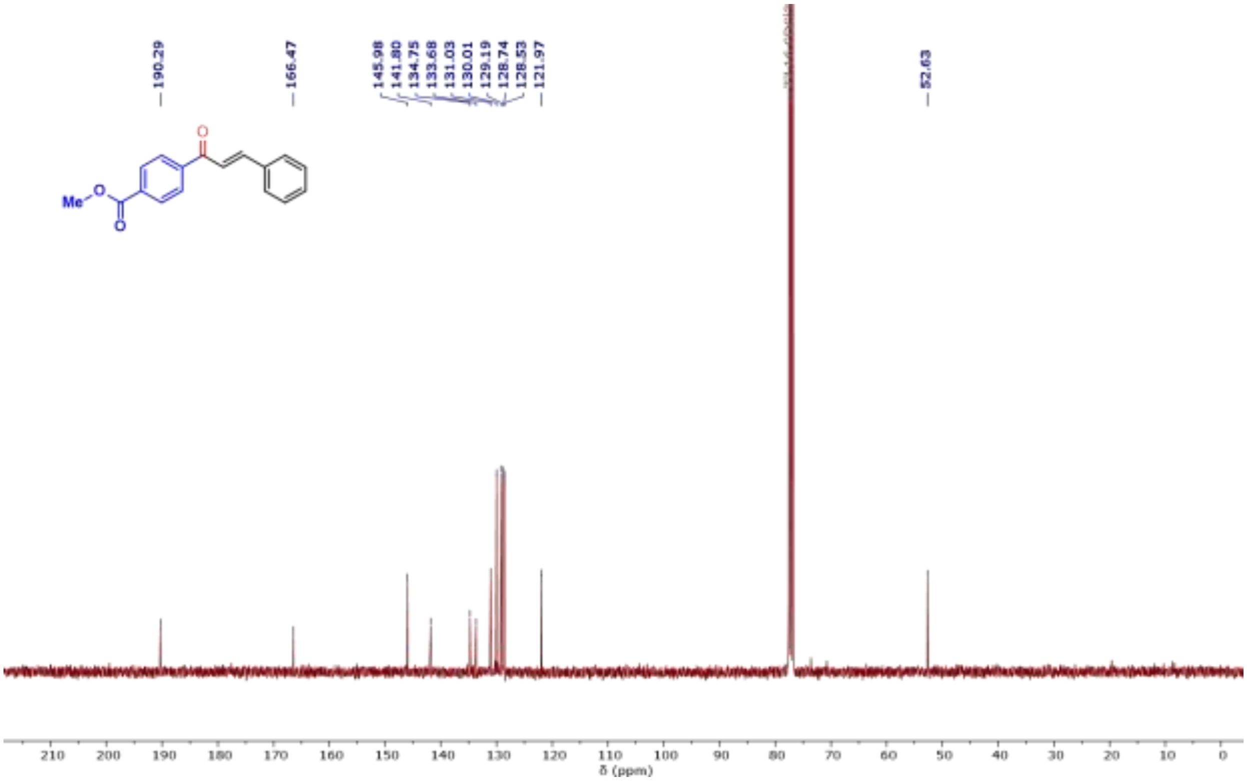

Figure S47. ^{13}C NMR (101 MHz, $CDCl_3$) spectrum of product **4o.**

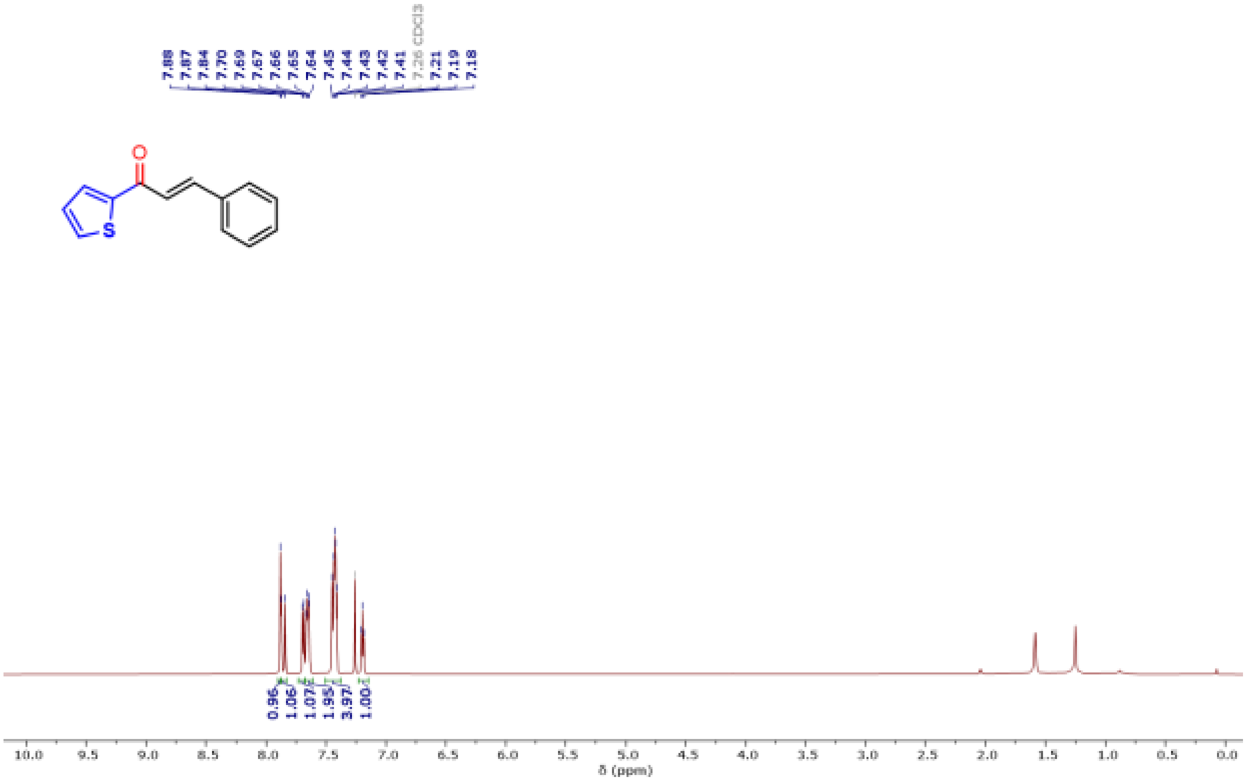

Figure S48. ^{1}H NMR (400 MHz, $CDCl_3$) spectrum of product **4r.**

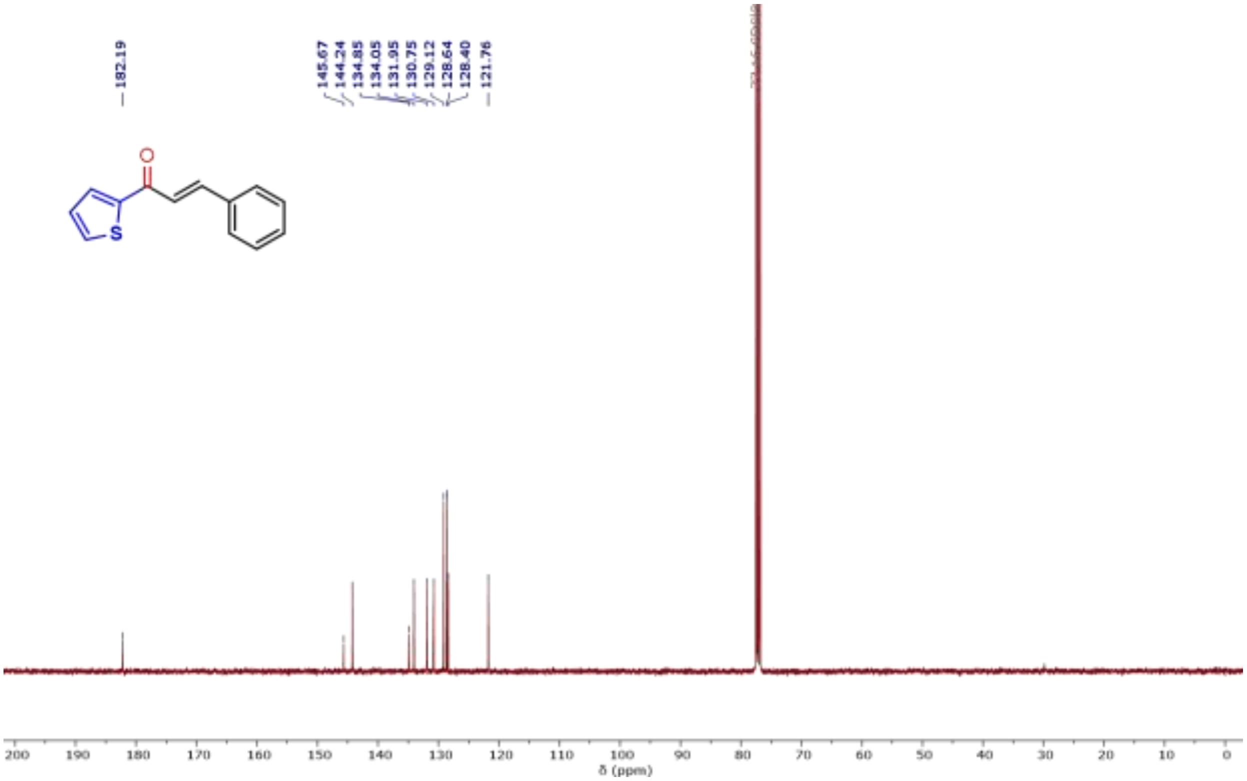

Figure S49. ^{13}C NMR (101 MHz, $CDCl_3$) spectrum of product **4r.**

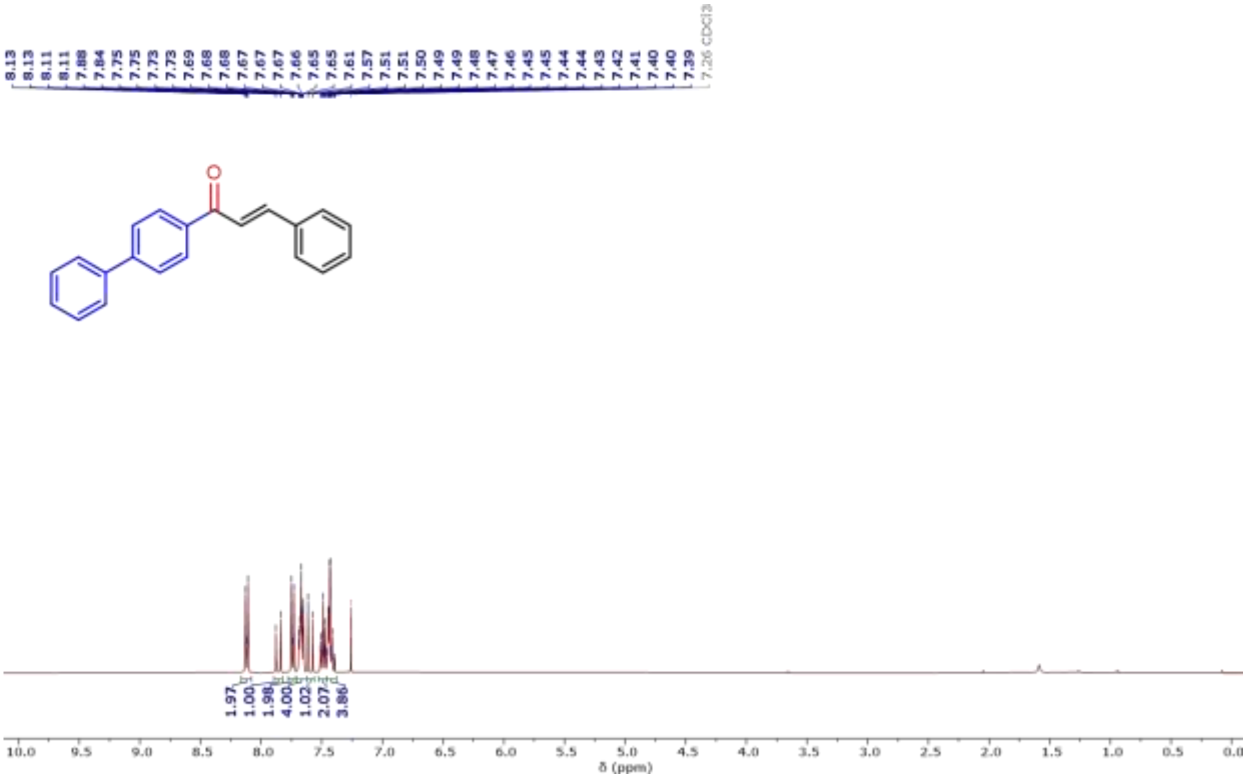

Figure S50. ^{1}H NMR (400 MHz, $CDCl_3$) spectrum of product **4u.**

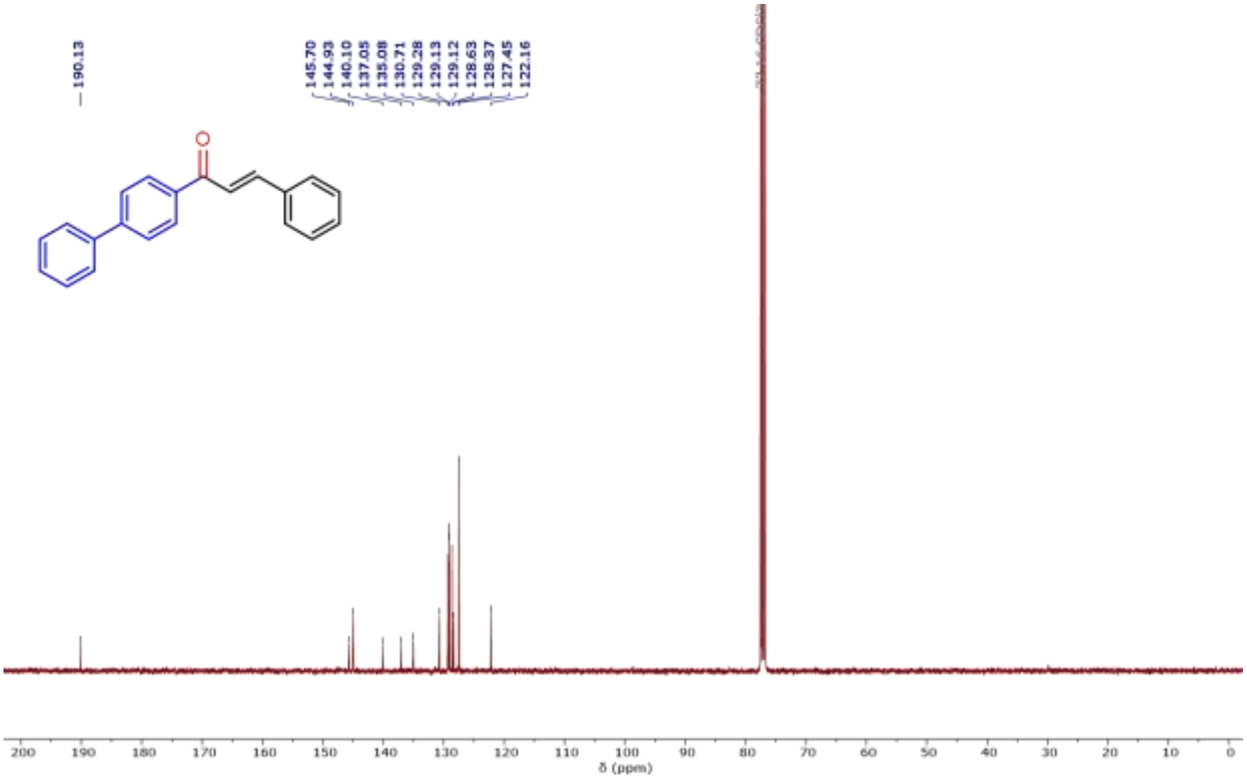

Figure S51. ^{13}C NMR (101 MHz, $CDCl_3$) spectrum of product **4u.**

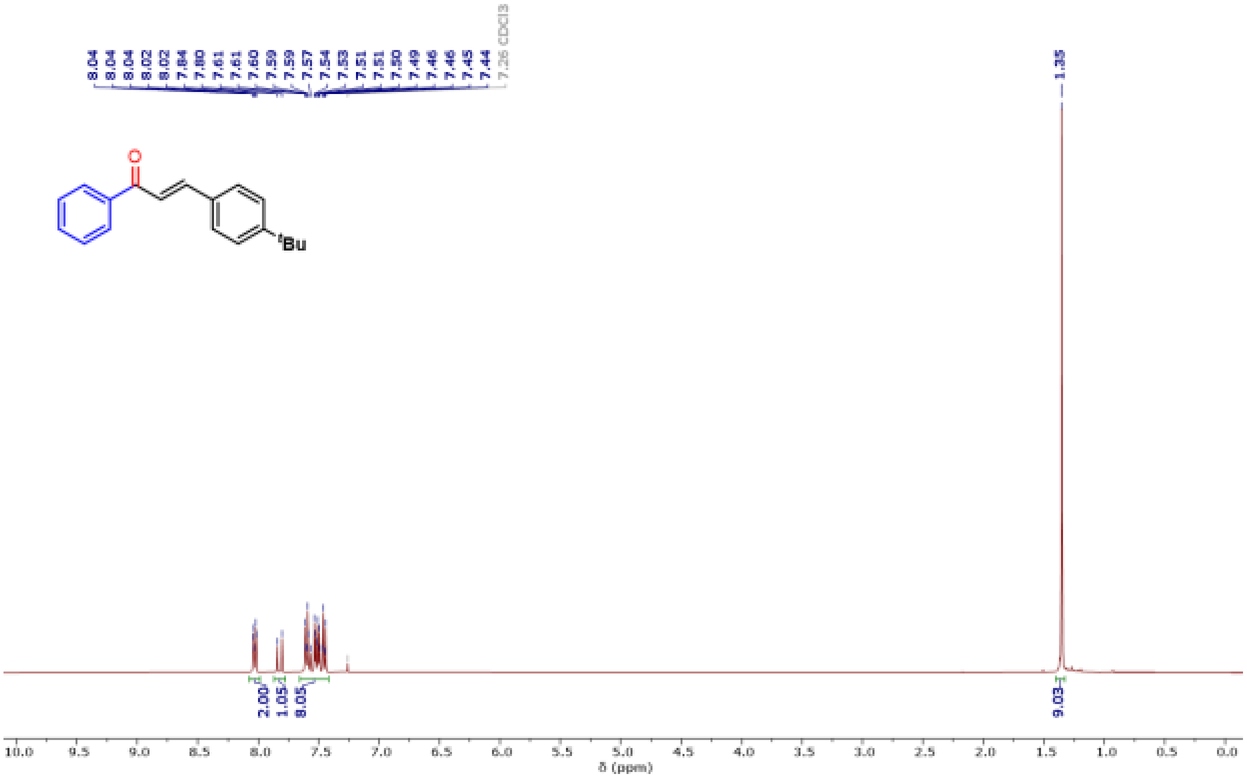

Figure S52. ^1H NMR (400 MHz, $CDCl_3$) spectrum of product **4y.**

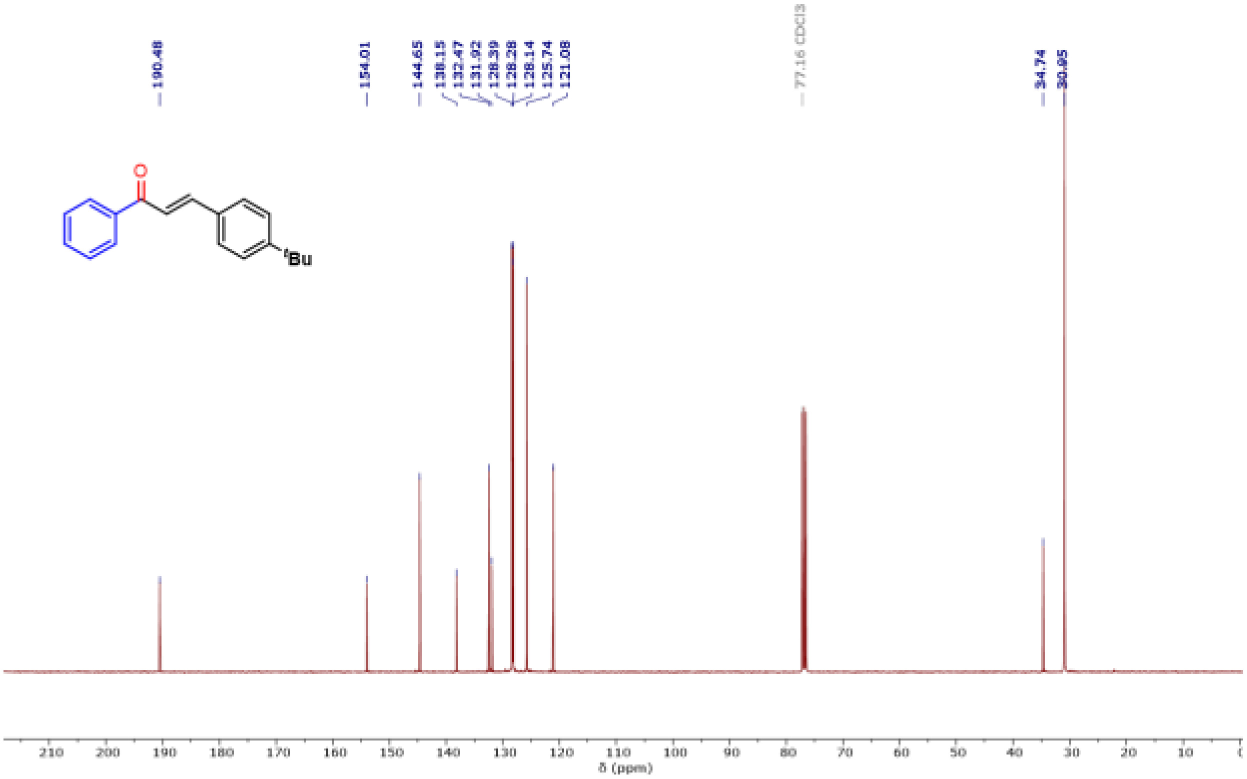

Figure S53. ^{13}C NMR (101 MHz, $CDCl_3$) spectrum of product **4y.**

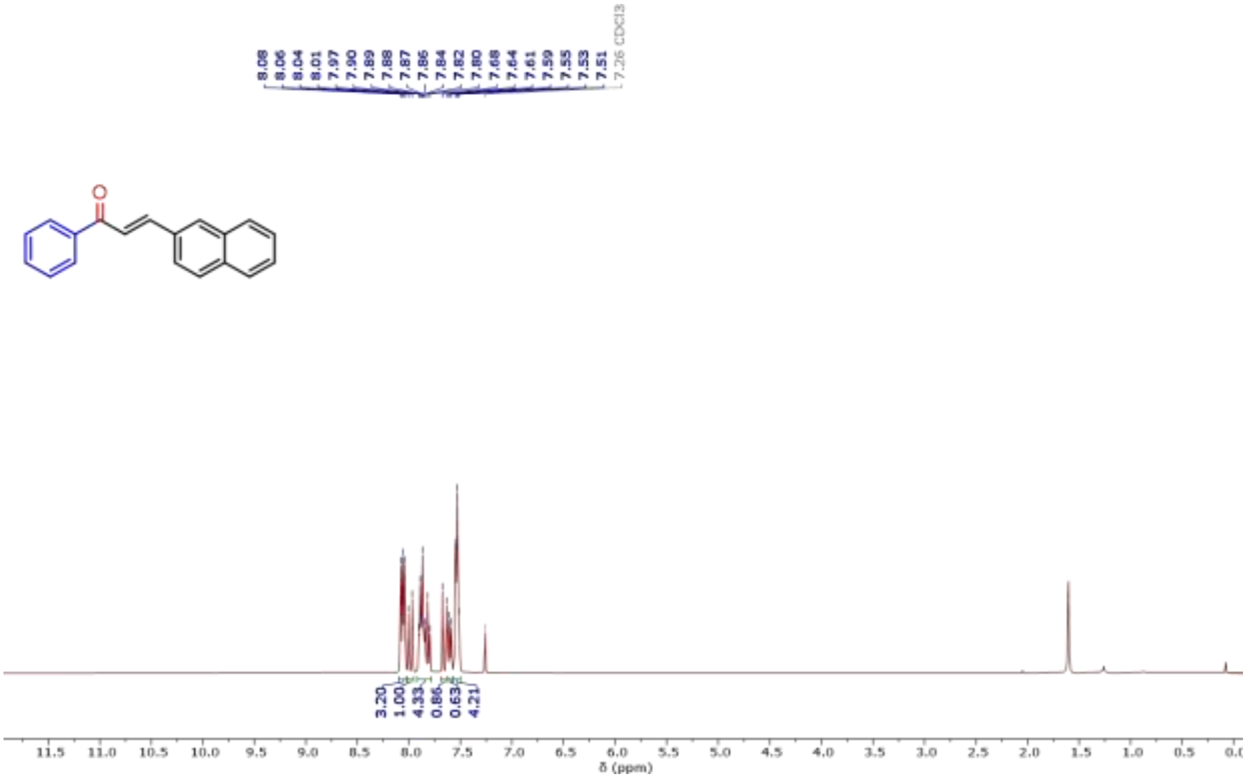

Figure S54. ^{1}H NMR (400 MHz, $CDCl_3$) spectrum of product **4ag.**

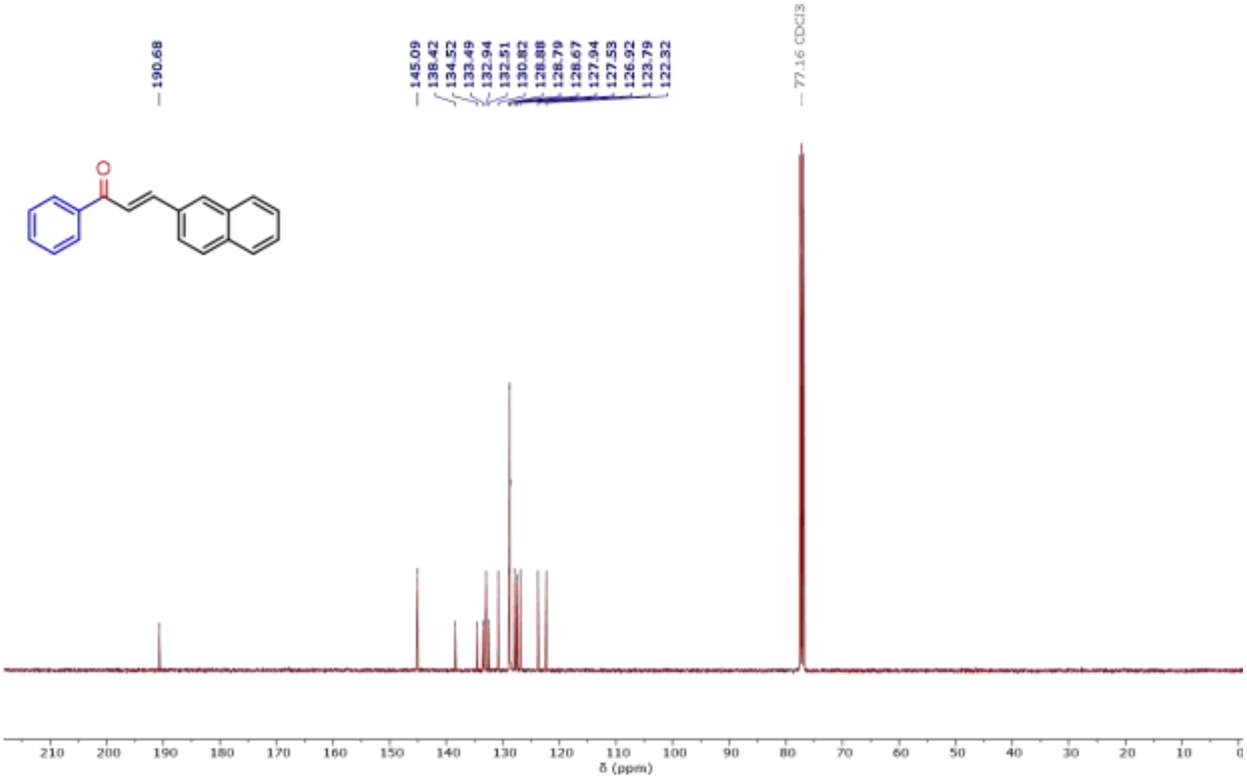

Figure S55. ^{13}C NMR (101 MHz, $CDCl_3$) spectrum of product **4ag.**

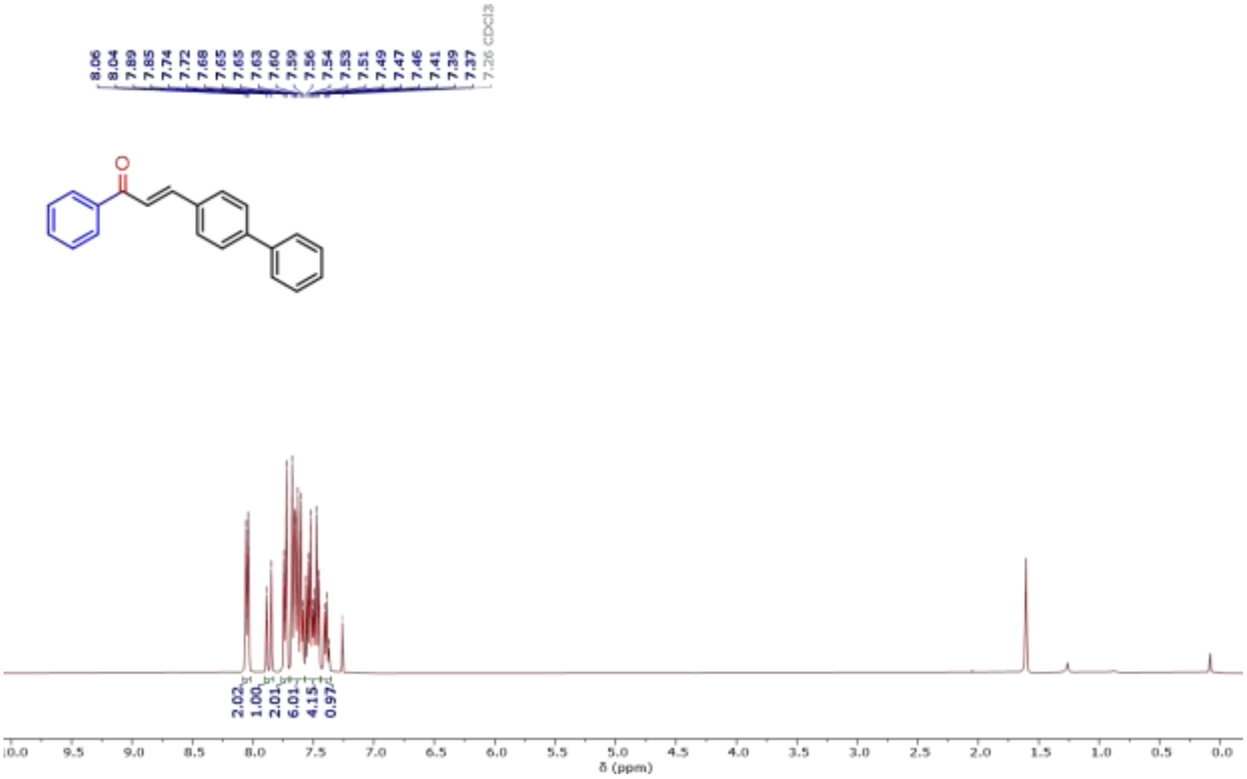

Figure S56. ^{1}H NMR (400 MHz, $CDCl_3$) spectrum of product **4ah.**

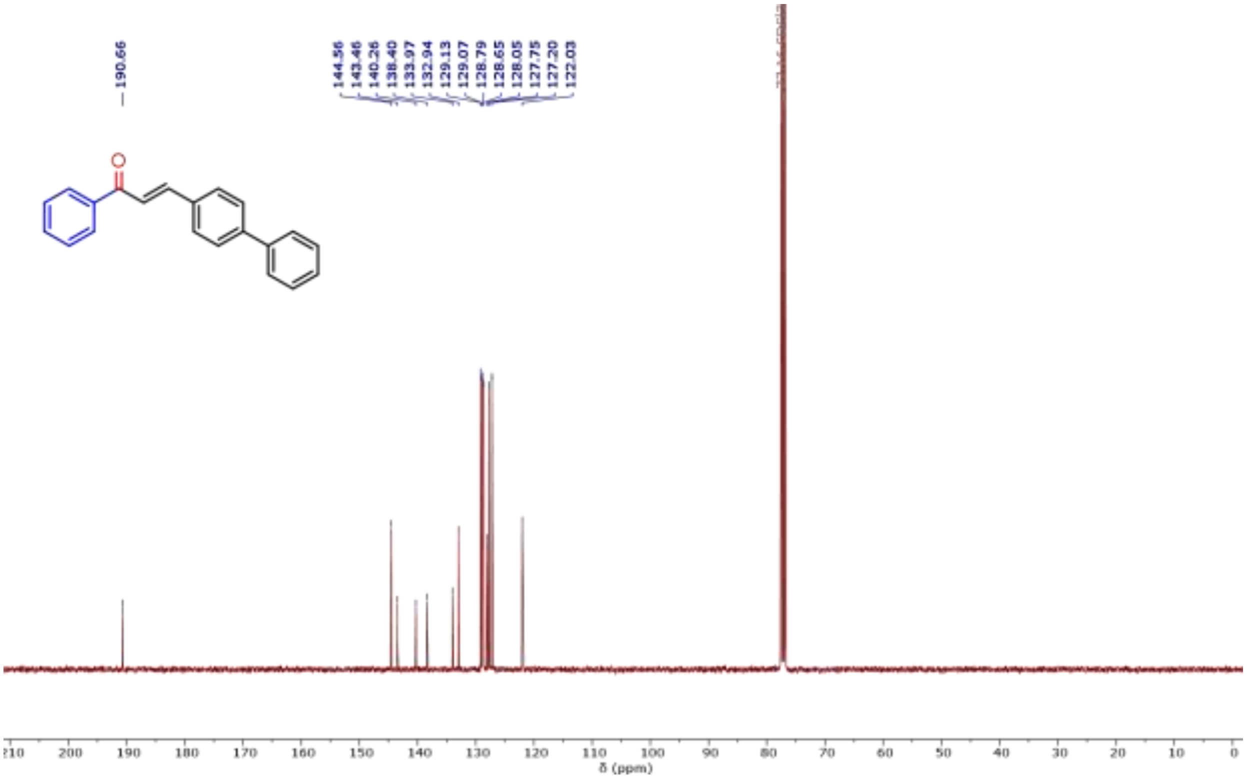

Figure S57. ^{13}C NMR (101 MHz, $CDCl_3$) spectrum of product **4ah.**

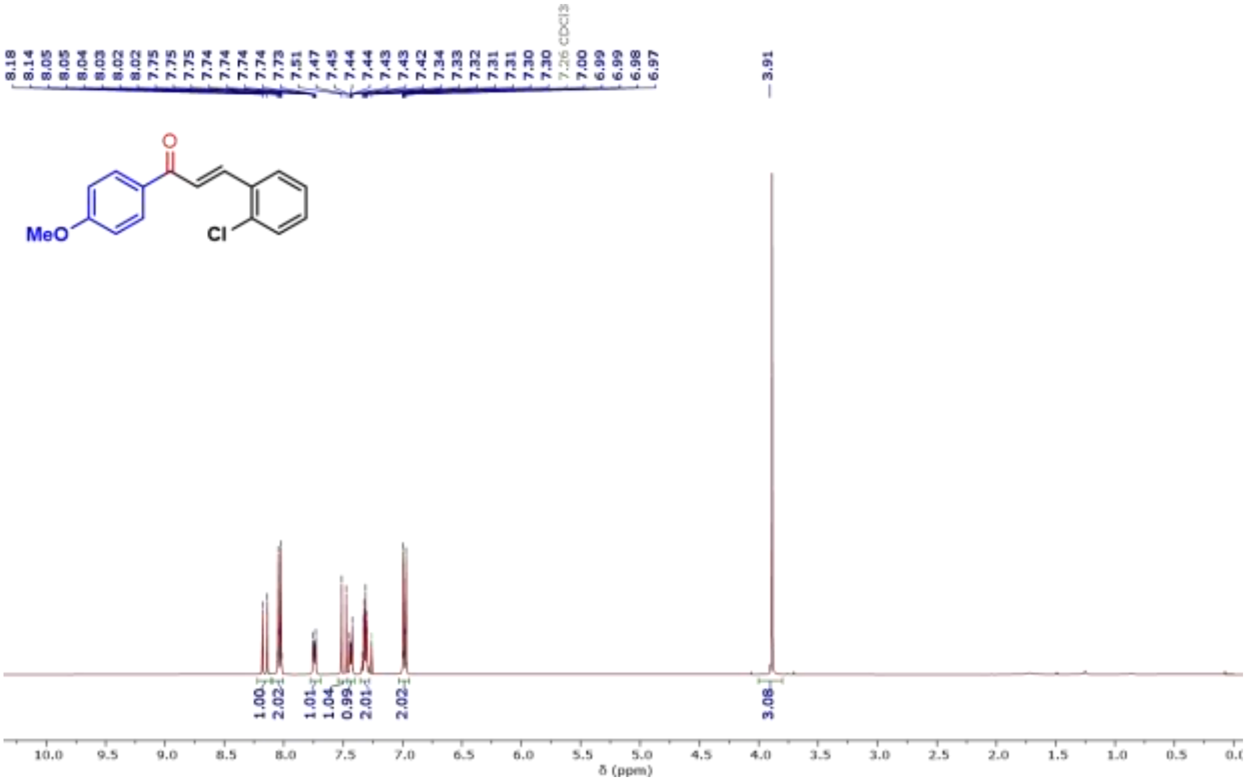

Figure S58. ^{1}H NMR (400 MHz, $CDCl_3$) spectrum of product **4ai.**

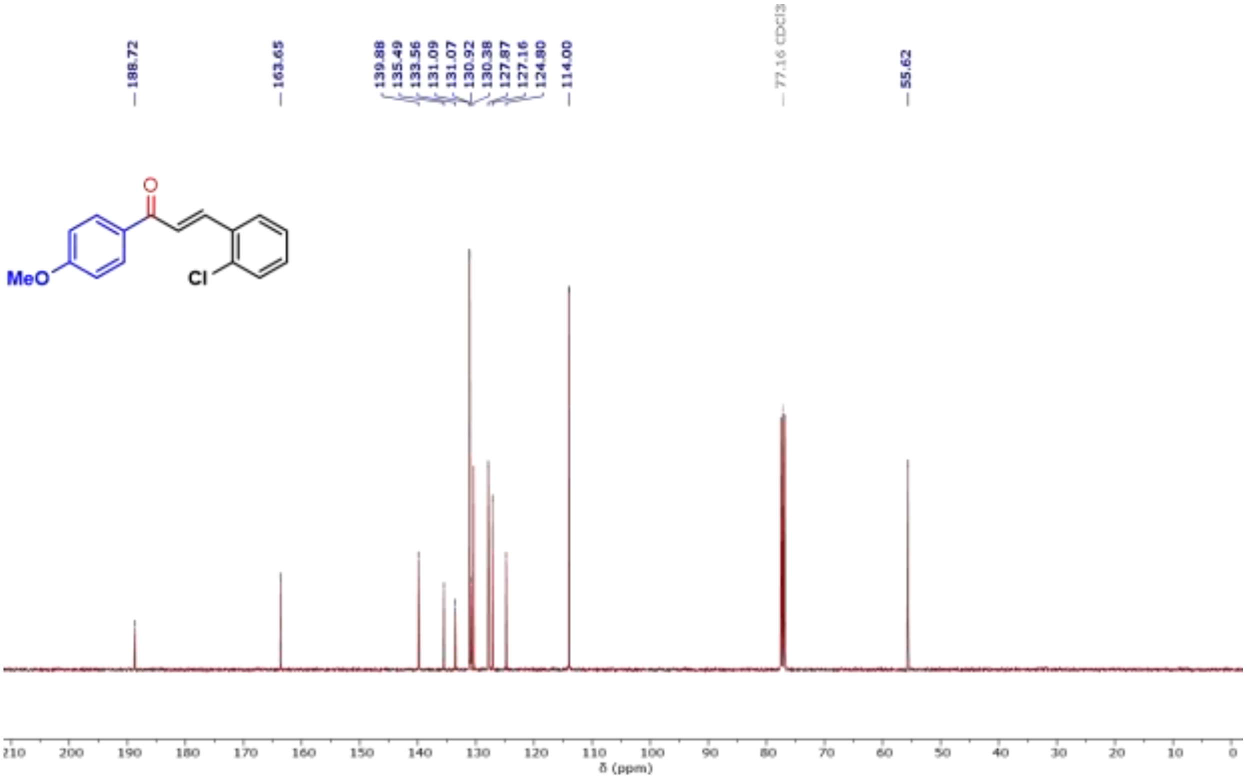

Figure S59. ^{13}C NMR (101 MHz, $CDCl_3$) spectrum of product **4ai.**

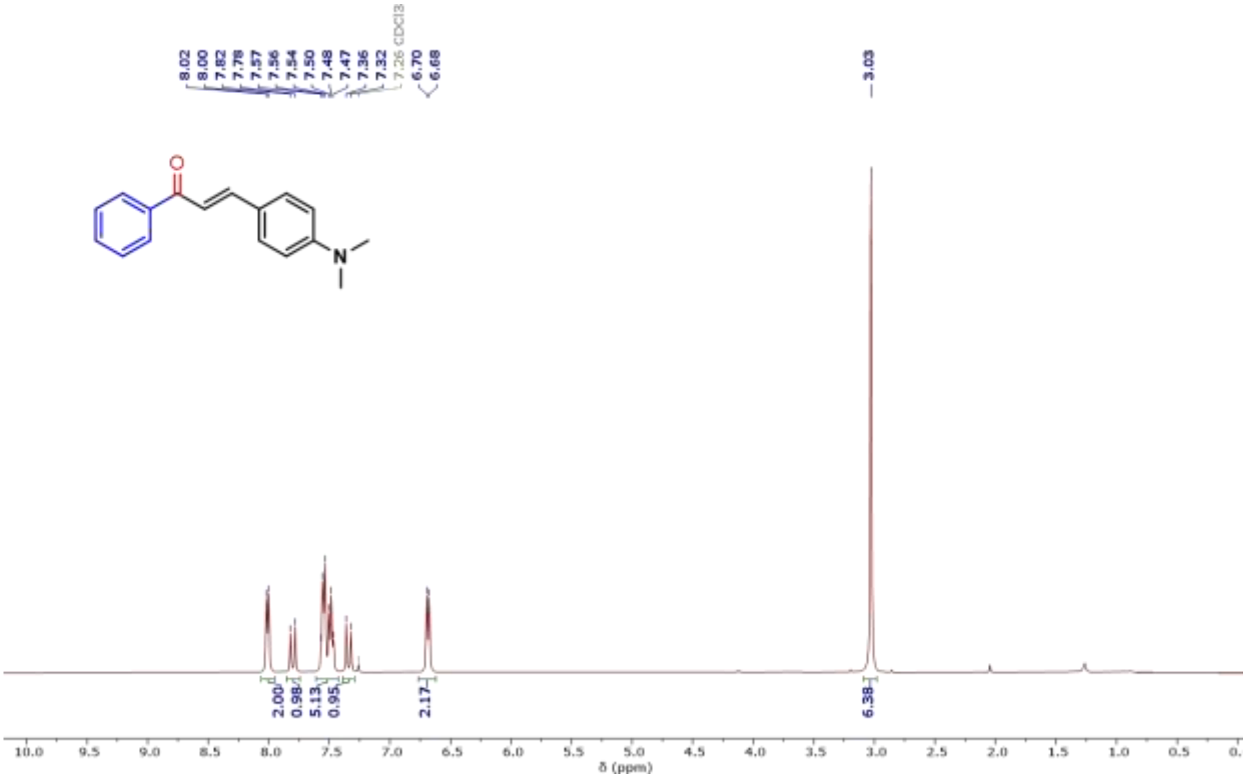

Figure S60. ^{1}H NMR (400 MHz, $CDCl_3$) spectrum of product **4ak.**

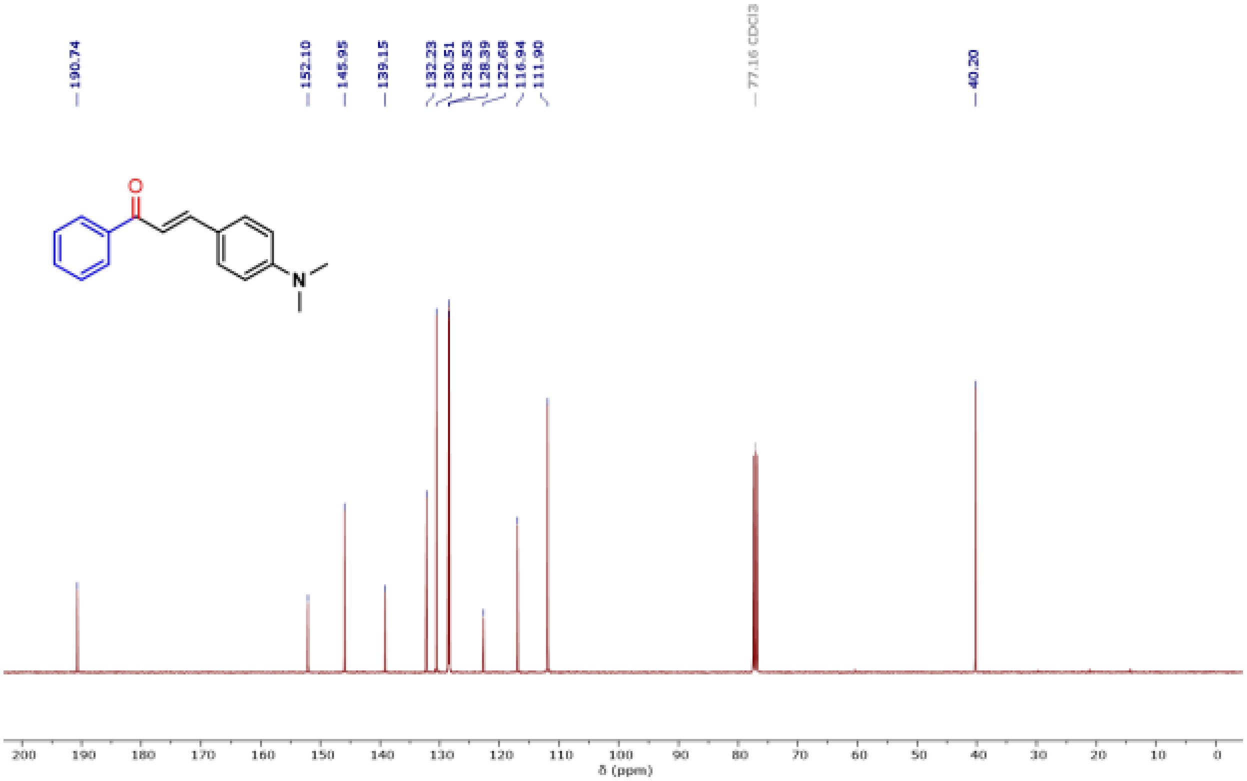

Figure S61. ^{13}C NMR (101 MHz, $CDCl_3$) spectrum of product **4ak.**

References

1. a) Hediger, S.; Meier, B. H.; Ernst, R. R., Adiabatic passage Hartmann-Hahn cross polarization in NMR under magic angle sample spinning. *Chem. Phys. Lett.* **1995,** *240*, 449-456. b) Hediger, S.; Meier, B. H.; Kurur, N. D.; Bodenhausen, G.; Ernst, R. R., NMR cross polarization by adiabatic passage through the Hartmann—Hahn condition (APHH). *Chem. Phys. Lett.* **1994,** *223*, 283-288.
2. Fung, B. M.; Khitrin, A. K.; Ermolaev, K., An Improved Broadband Decoupling Sequence for Liquid Crystals and Solids. *J. Magn. Reson.* **2000,** *142*, 97-101.
3. Massiot, D.; Fayon, F.; Capron, M.; King, I.; Le Calvé, S.; Alonso, B.; Durand, J. O.; Bujoli, B.; Gan, Z. H.; Hoatson, G. Modelling one- and two-dimensional solid-state NMR spectra. *Magn. Reson. Chem.*, **2002**, *40* (1), 70-76.
4. Luska, K. L.; Julis, J.; Stavitski, E.; Zakharov, D. N.; Adams, A.; Leitner, W. Bifunctional nanoparticle–SILP catalysts (NPs@SILP) for the selective deoxygenation of biomass substrates. *Chem. Sci.* **2014**, *5*, 4895–4905.
5. a) Bordet, A.; Moos, G.; Welsh, C.; Licence, P.; Luska, K. L.; Leitner, W., Molecular Control of the Catalytic Properties of Rhodium Nanoparticles in Supported Ionic Liquid Phase (SILP) Systems. *ACS Catal.* **2020,** *10*, 13904-13912. b) Louis Anandaraj, S. J.; Kang, L.; DeBeer, S.; Bordet, A.; Leitner, W., Catalytic Hydrogenation of CO2 to Formate Using Ruthenium Nanoparticles Immobilized on Supported Ionic Liquid Phases. *Small* **2023,** *19*, e2206806.
6. Gardiner, M. G.; Herrmann, W. A.; Reisinger, C.-P.; Schwarz, J.; Spiegler, M., Dicationic chelating N-heterocyclic carbene complexes of palladium: new catalysts for the copolymerisation of C_2H_4 and CO. *J. Organomet. Chem.* **1999,** *572*, 239-247.
7. Wu, X. F.; Neumann, H.; Spannenberg, A.; Schulz, T.; Jiao, H.; Beller, M. Development of a general palladium-catalyzed carbonylative Heck reaction of aryl halides. *J. Am. Chem. Soc.* **2010**, *132* (41), 14596-14602.
8. Albaladejo, M. J.; Alonso, F.; González-Soria, M. J. Synthetic and Mechanistic Studies on the Solvent-Dependent Copper-Catalyzed Formation of Indolizines and Chalcones. *ACS Catal.* **2015**, *5* (6), 3446-3456.
9. Gopalakrishnan, M.; Thanusu, J.; Kanagarajan, V.; Govindaraju, R. Synthesis, antibacterial, and antifungal activities of biolabile (E)-1-(4-morpholinophenyl)-3-aryl-prop-2-en-1-ones. *Med Chem Res* **2009**, *18* (5), 341-350.

10. Shaykhutdinova, P.; Oestreich, M. Achieving Enantioselectivity in Difficult Cyclohexa-1,3-diene Diels-Alder Reactions with Sulfur-Stabilized Silicon Cations as Lewis Acid Catalysts. *Org. Lett.* **2018**, *20* (22), 7029-7033.
11. Thomson, C. J.; Barber, D. M.; Dixon, D. J. One-Pot Catalytic Enantioselective Synthesis of 2-Pyrazolines. *Angew. Chem. Int. Ed.* **2019**, *58* (8), 2469-2473.
12. Wu, J.; Li, J.; Cai, Y.; Pan, Y.; Ye, F.; Zhang, Y.; Zhao, Y.; Yang, S.; Li, X.; Liang, G. Evaluation and discovery of novel synthetic chalcone derivatives as anti-inflammatory agents. *J. Med. Chem.* **2011**, *54* (23), 8110-8123.
13. Zhou, B.; Jiang, P.; Lu, J.; Xing, C. Characterization of the Fluorescence Properties of 4-Dialkylaminochalcones and Investigation of the Cytotoxic Mechanism of Chalcones. *Arch. Pharm.* **2016**, *349* (7), 539-552.
